# Technology roadmap from fiber materials to fiber electronics

**DOI:** 10.1093/nsr/nwag368

**Published:** 2026-06-12

**Authors:** Songlin Zhang, Lin Chen, Peining Chen, Wenguo Cui, Bin Ding, Lingyan Ding, Chao Gao, Ya Huang, Ting Lei, Meng Liao, Qingwen Li, Yingdong Li, Fangbing Lin, Jiajun Qin, Yajie Qin, Li Shen, Xuemei Sun, Kai Tang, Guangming Tao, Bingjie Wang, Songlin Xie, Kaiwen Zeng, Wanxin Zhang, Yingying Zhang, Chen Zhao, Zijian Zheng, Zhengfeng Zhu, Huisheng Peng

**Affiliations:** State Key Laboratory of Molecular Engineering of Polymers, Department of Macromolecular Science, Institute of Fiber Materials and Devices, Laboratory of Advanced Materials, Fudan University, Shanghai 200438, China; College of Integrated Circuits and Micro-Nano Electronics, School of Microelectronics, State Key Laboratory of Integrated Chip and System, Fudan University, Shanghai 200433, China; State Key Laboratory of Molecular Engineering of Polymers, Department of Macromolecular Science, Institute of Fiber Materials and Devices, Laboratory of Advanced Materials, Fudan University, Shanghai 200438, China; Department of Orthopaedics, Shanghai Key Laboratory for Prevention and Treatment of Bone and Joint Diseases, Shanghai Institute of Traumatology and Orthopaedics, Ruijin Hospital, Shanghai Jiao Tong University School of Medicine, Shanghai 200025, China; School of Energy and Materials, Shanghai Polytechnic University, Shanghai 201209, China; Innovation Center for Textile Science and Technology, Donghua University, Shanghai 200051, China; National Key Laboratory of Human Factors Engineering, China Astronaut Research and Training Center, Beijing 100094, China; MOE Key Laboratory of Macromolecular Synthesis and Functionalization, Department of Polymer Science and Engineering, Research Center for Advanced Fibers, Zhejiang University, Hangzhou 310058, China; State Key Laboratory of Molecular Engineering of Polymers, Department of Macromolecular Science, Institute of Fiber Materials and Devices, Laboratory of Advanced Materials, Fudan University, Shanghai 200438, China; National Key Laboratory of Advanced Micro and Nano Manufacture Technology, Key Laboratory of Polymer Chemistry and Physics of Ministry of Education, School of Materials Science and Engineering, Peking University, Beijing 100871, China; State Key Laboratory of Molecular Engineering of Polymers, Department of Macromolecular Science, Institute of Fiber Materials and Devices, Laboratory of Advanced Materials, Fudan University, Shanghai 200438, China; Key Laboratory of Multifunctional Nanomaterials and Smart Systems, Suzhou Institute of Nano-Tech and Nano-Bionics, Chinese Academy of Sciences, Suzhou 215123, China; School of Nano-Tech and Nano-Bionics, University of Science and Technology of China, Hefei 230026, China; Division of Nanomaterials and Jiangxi Key Lab of Carbonene Materials, Jiangxi Institute of Nanotechnology, Nanchang 330200, China; Tayho Advanced Materials Group Co., Ltd., Yantai 264006, China; Tayho Advanced Materials Group Co., Ltd., Yantai 264006, China; State Key Laboratory of Molecular Engineering of Polymers, Department of Macromolecular Science, Institute of Fiber Materials and Devices, Laboratory of Advanced Materials, Fudan University, Shanghai 200438, China; College of Biomedical Engineering, Fudan University, Shanghai 200438, China; Department of Cardiology, National Clinical Research Center for Interventional Medicine, State Key Laboratory of Cardiovascular Diseases, Zhongshan Hospital, Fudan University, Shanghai 200433, China; State Key Laboratory of Molecular Engineering of Polymers, Department of Macromolecular Science, Institute of Fiber Materials and Devices, Laboratory of Advanced Materials, Fudan University, Shanghai 200438, China; Tayho Advanced Materials Group Co., Ltd., Yantai 264006, China; Research Center for Intelligent Fiber Devices and Equipment, Wuhan National Laboratory for Optoelectronics, School of Materials Science and Engineering, and School of Physical Education, Huazhong University of Science and Technology, Wuhan 430074, China; State Key Laboratory of Molecular Engineering of Polymers, Department of Macromolecular Science, Institute of Fiber Materials and Devices, Laboratory of Advanced Materials, Fudan University, Shanghai 200438, China; Huawei Device Co., Ltd., Shenzhen 518129, China; State Key Laboratory of Molecular Engineering of Polymers, Department of Macromolecular Science, Institute of Fiber Materials and Devices, Laboratory of Advanced Materials, Fudan University, Shanghai 200438, China; National Key Laboratory of Human Factors Engineering, China Astronaut Research and Training Center, Beijing 100094, China; Key Laboratory of Organic Optoelectronics and Molecular Engineering of the Ministry of Education, Department of Chemistry, Tsinghua University, Beijing 100084, China; Division of Nanomaterials and Jiangxi Key Lab of Carbonene Materials, Jiangxi Institute of Nanotechnology, Nanchang 330200, China; Research Institute for Intelligent Wearable Systems (RI-IWEAR), Department of Applied Biology and Chemical Technology, Faculty of Science, Research Institute for Smart Energy (RISE), The Hong Kong Polytechnic University, Hong Kong 999077, China; State Key Laboratory of Molecular Engineering of Polymers, Department of Macromolecular Science, Institute of Fiber Materials and Devices, Laboratory of Advanced Materials, Fudan University, Shanghai 200438, China; State Key Laboratory of Molecular Engineering of Polymers, Department of Macromolecular Science, Institute of Fiber Materials and Devices, Laboratory of Advanced Materials, Fudan University, Shanghai 200438, China

**Keywords:** fiber materials, fiber electronics, textile intelligence, wearable electronics, implantable electronics

## Abstract

Fiber electronics, one-dimensional structures with active electronic functions while maintaining the flexibility of traditional fibers or textiles, represent a paradigm shift in electronic technologies, transforming passive fiber or textile substrates into active, intelligent systems that integrate powering, sensing, computation, and communication capabilities. This field has rapidly evolved from the development of simple conductive threads to the creation of sophisticated, multifunctional fiber devices. Significant progress is underpinned by the design of functional fiber materials with exceptional electrical, mechanical, and thermal properties for diverse fiber devices, such as batteries, solar cells, light-emitting displays, and neuromorphic computing devices, and these materials can be further woven into everyday fabrics. However, the transition from laboratory demonstrations to real-world applications faces significant interdisciplinary challenges, including reproducible large-scale manufacturing, the creation of robust, washable interconnects, and the establishment of community standards for reliability and qualification. To address these challenges, this review synthesizes recent progress and outlines a forward-looking roadmap. We emphasize the need for convergent research focusing on adaptive materials, fiber-native device architectures, intelligent manufacturing processes, and holistic system-level integrations. Overcoming these hurdles will unlock the full potential of fiber electronics, paving the way for transformative applications in personalized healthcare, human–machine interfaces, and extreme-environment systems, ultimately weaving the textile intelligence seamlessly into the fabric of daily life.

## Teaser

By bridging the chasm between single-fiber demonstrations and scalable engineering, fiber electronics—spanning sensing, energy, display and circuit—are poised to redefine human-material interaction through fully integrated, system-level textile intelligence.

## INTRODUCTION

**Figure 1.**
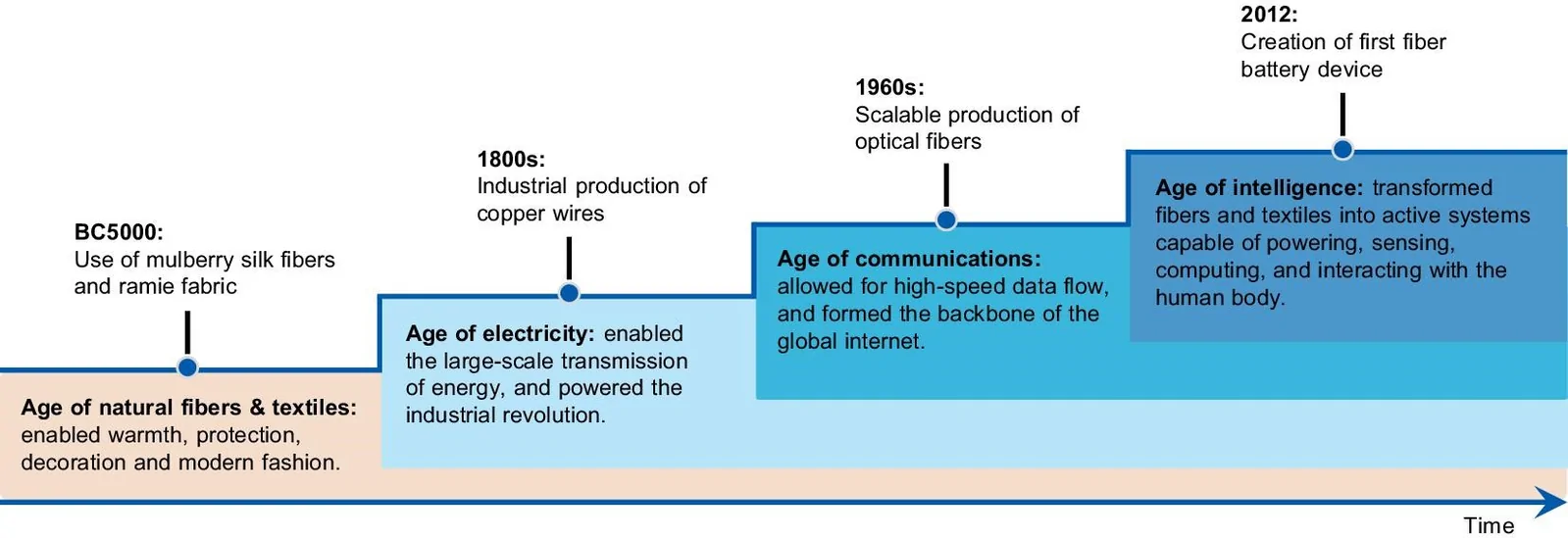
Historical evolution and technological milestones of fiber materials toward integrated-fiber electronic systems. The timeline illustrates the transformative trajectory of fibers across four distinct eras, highlighting the shift from passive structures to active, intelligent systems, including the age of natural fibers and textiles (starting 5000 BC), the age of electricity (1800s), the age of communications (1960s), and the age of intelligence (2012–Present). This progression culminates in the pursuit of the system-in-a-fiber paradigm, where complex electronic functions are natively and seamlessly embedded within a single filamentary substrate to interact directly with the human body.

The history of fiber materials is a testament to the expansion of functional dimensions within one-dimensional (1D) architectures, repeatedly redefining the boundaries of human civilization and industrial capability. This evolutionary trajectory spans from the ancient mastery of natural silk weaving in China over 5000 years ago to the 20th-century breakthroughs in synthetic polymers and metallic wires. In particular, the industrial-scale production of metal wires underpins the electrical revolution, expanding the functional scope of fibers beyond structure and textiles into electrical power transmission (Fig. 1). These milestones decouple fiber properties from natural constraints, enabling precise control over mechanical strength

and electrical performance. The subsequent advent of optical fibers further transformed the 1D geometry from a mere carrier of physical loads or currents into a sophisticated medium for global information networks. Together, these historical achievements illustrate how the fiber form factor has repeatedly served as both a material platform and a manufacturing paradigm that drives major technological revolutions.

In the 21st century, we stand at a new inflection point in the transition from passive fibers as substrates to active fibers as intelligent, multifunctional systems with capabilities of powering, sensing, processing, and interaction [1–5]. In this context, we prioritize fiber materials that contribute to active electronic functions rather than passive structural support. Thus, we define fiber electronics (or fiber devices) as filamentary structures that integrate one or more active functions (e.g. energy harvesting or storage, light emission or pixel display, physical or chemical sensing, data processing or signal transmission) while retaining the mechanical compliance, flexibility, and textile compatibility intrinsic to fibers. Despite the maturity of planar and flexible electronics, the fiber form factor of fiber electronics offers a unique set of physicochemical and mechanical advantages that are qualitatively distinct. Unlike thin-film devices, fiber electronics possess a high surface-area-to-volume ratio and intrinsic mechanical anisotropy, allowing them to undergo complex deformations (e.g. twisting, stretching, and 3D interlacing) without compromising functional integrity. This 1D geometry acts as a bridge between the microscopic world of active nanomaterials and the macroscopic world of wearable electronic textiles. By mimicking the hierarchical structure of traditional yarns, fiber devices can be seamlessly integrated into breathable, conformal, and biocompatible matrices that conform to bodies and environments, transforming everyday objects into interactive interfaces without sacrificing the comfort or aesthetics of conventional fabrics.

However, the field is currently transitioning from an era of isolated proof-of-concept fiber devices to integrated-fiber electronic systems (IFES) with multiple functions. Indeed, remarkable performance in individual fiber devices has been reported over the past decade. For instance, in information interfaces, luminescent fiber devices and pixelated textile displays suggest new modes of human–machine interaction where garments serve directly as communication and display surfaces [1]. In energy systems, combining energy-harvesting and energy-storage fibers within structural components supports a structure as a power source paradigm, increasing functional and energy density in portable electronics, robotics, and vehicles [6,7]. In healthcare, soft wearable and implantable sensing fiber devices enable minimally invasive, continuous monitoring of physiological and biochemical signals, offering new routes for long-term, personalized diagnostics [8,9]. In aerospace and extreme environments, multifunctional fiber architectures, through integrating sensing, thermal protection, electromagnetic shielding, or actuation, provide capabilities that rigid electronics cannot, supporting conformal life-support garments, adaptive shielding, and deployable structures. Despite these advances in shifting fiber electronics from passive substrates to foundational, versatile platforms, the synergistic integration of diverse fiber devices into a cohesive, autonomous system remains a formidable challenge. The scientific community faces a critical bottleneck in which optimizing a single material property (e.g. high electrical conductivity) often conflicts with system-level requirements (e.g. long-term functional stability after industrial-standard washing). Consequently, the realization of a truly fiber-native electronic system demands a fundamental shift in design philosophy: from focusing on material performance in isolation to engineering the material‒device‒system continuum.

In this review, we provide a technology roadmap to bridge the gap between fundamental fiber device science and scalable integrated-fiber electronic systems. We first summarize the major material systems, spanning classic carbon-based materials to emerging metal-backboned polymers, and the structure–property relationships that govern their processability and performance. We then examine the fabrication strategies and architectures developed for versatile fiber devices, together with discussions on interfacial engineering principles and structural assembly strategies that enable multifunctional integration. By critically evaluating representative applications, ranging from personalized healthcare to human‒machine interactions, we identify the cross-cutting scientific bottlenecks that currently impede large-scale deployment. In each section, we also propose specific forward-looking research directions that may link fundamental material and device innovation with scale-up production technologies. Finally, we propose a strategic translation roadmap that aligns material synthesis and device fabrication with intelligent manufacturing and standardized evaluation protocols. The goal is to provide a coherent, system-level framework that supports both the scientific discovery and the scalable deployment of next-generation integrated-fiber electronic systems.

## FUNCTIONAL FIBER MATERIALS

The realization of advanced fiber electronics hinges on the development of functional materials that transcend the passive structural roles of conventional fibers. This chapter establishes the foundational framework for fiber devices by systematically reviewing the principal classes of functional fibers, spanning carbon-based, metallic, polymeric, and inorganic systems. We focus on advanced fabrication paradigms, ranging from gas-phase growth and array drawing to diverse spinning and thermal drawing techniques, which transform raw precursors into functional fibers or hybrid threads. In particular, emphasis is placed on how multiscale assembly and composite strategies enable precise deterministic control over electrical, thermal, and mechanical properties across the hierarchical fiber-yarn-fabric continuum. For each material platform, we provide a critical analysis that not only catalogs representative functional demonstrations and performance metrics but also assesses their manufacturability and scaling potential. By identifying the key technical bottlenecks that currently hinder translating laboratory prototypes into mass-producible, industry-ready products, we aim to provide a basis for the rational design of next-generation fiber materials.

### Carbon nanotube fibers

**Figure 2.**
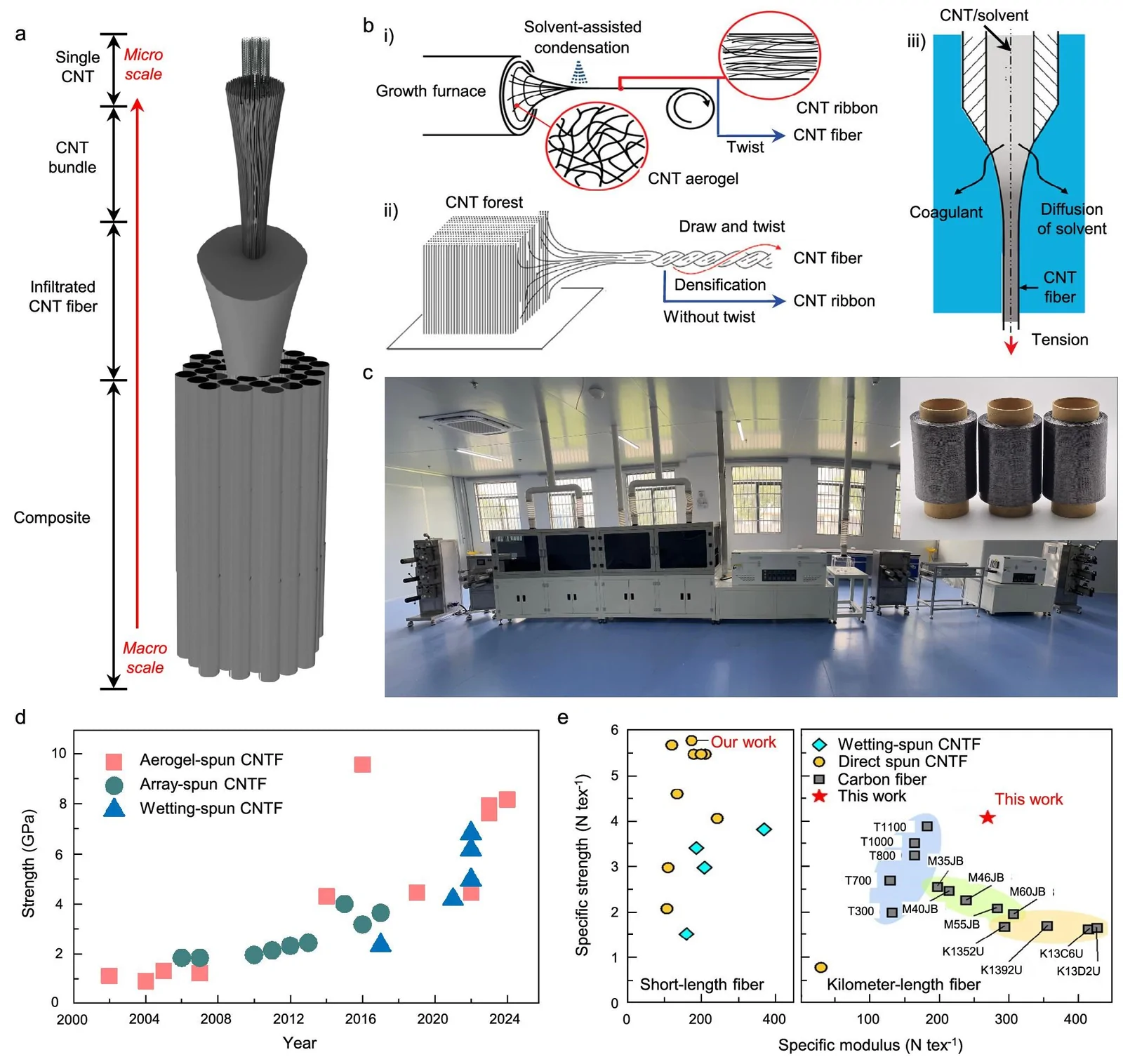
Hierarchical architectures, fabrication methodologies, and mechanical performance evolution of carbon nanotube (CNT) fibers. (a) Schematic representation of the structural evolution of CNTs across multiple length scales from individual nanotubes to CNT bundles, infiltrated fiber architectures, and macroscale fiber composites. (b) Overview of the primary industrial paradigms for the production of continuous CNT fibers: (i) the floating catalyst chemical vapor deposition (FCCVD) method, involving the direct spinning of a CNT aerogel from a growth furnace; (ii) solid-state drawing and twisting from a vertically aligned CNT forest (array-spinning); and (iii) solution-based wet spinning from a liquid-crystalline CNT/solvent dispersion through a coagulant bath (adapted from [13]). (c) Photograph of industrial-scale continuous processing equipment and spools of kilometer-length CNT fibers (adapted from [14]). (d and e) A summary of the tensile strength evolution of CNT fibers fabricated *via* different spinning routes, and an Ashby-style plot comparing the specific strength and specific modulus of current CNT fibers against commercial high-performance carbon fibers (adapted from [15]).

Carbon nanotubes (CNTs), characterized by their sp²-bonded, one-dimensional graphitic structure, combine exceptional mechanical strength, high electrical conductivity, and outstanding thermal transport, resulting in the moniker of the ultimate polymer. The scientific challenge in this domain lies in translating these extraordinary nanoscale properties into macroscopic fibers through controlled assembly (Fig. 2a) [10,11]. At the structural level, high-performance fibers emerge from long, well-aligned nanotubes organized into dense bundles, where the architecture maximizes axial load transfer and charge/phonon transport. Concurrently, the intrinsic intertube sliding and hierarchical bundling confer a unique combination of flexibility and toughness, attributes that effectively overcome the brittleness of conventional carbon fibers while enhancing composite processability and wearability. The manufacturing pathways for continuous CNT fibers have evolved into two principal families: gas-phase chemical vapor deposition (CVD) routes (including floating-catalyst CVD and array drawing) and solution-based wet-spinning [12]. Each route offers distinct advantages and inherent trade-offs in purity, alignment, throughput, and composability.

#### Floating-catalyst CVD

As a continuous growth-to-winding process, the floating-catalyst CVD (FCCVD) involves transporting vaporized carbon sources and catalyst precursors into a high-temperature reactor where CNTs nucleate into an aerogel sock, which is then densified into a continuous fiber (Fig. 2b(i)). Since the inception of FCCVD-produced fibers in 1998 [16,17], subsequent engineering efforts have increased the tensile strength from ~1 GPa [18] to ~9.8 N tex^−1^ (~9 GPa) [19]. Fibers were post-treated *via* strategies using mechanical pressing [12] and chlorosulfonic acid-assisted drawing [20], which significantly enhanced their macroscopic mechanical properties. Recent advances in the kilometer-scale fabrication of high-performance fibers have further stabilized these performance metrics [15,21,22]. Notably, in 2024, a strategy combining compositing with muscle training increased single-fiber static strength to ~8.2 GPa and dynamic strength to an unprecedented 14 GPa [14]. Consequently, this route provides an effective route for the direct preparation of functionalized fibers *via in situ* composite technology [23,24] and offers a favorable balance of performance and scalability, with kilogram-scale production now demonstrated by both academia and industry (Fig. 2c).

#### Array- or forest-drawing

This technique involves extracting fibers directly from vertically aligned CNT arrays grown on substrates (Fig. 2b(ii)). Since the first 30-cm fiber was drawn from a 100-µm array in 2002 [25], research has shown that twisting these membranes can substantially enhance their mechanical performance [26]. While array-drawn fibers offer superior purity and alignment, the process remains constrained by high costs and limited throughput. Recent scale-up demonstrations, such as batch growth on 8-inch wafers [27], have advanced, yet cost efficiency remains a critical hurdle for mass-market manufacturing.

#### Wet-spinning

This route adapts traditional polymer spinning concepts to concentrated CNT suspensions or liquid-crystalline dopes (Fig. 2b(iii)). Following early foundational studies (2000–2004) [28,29], breakthroughs in rheology control and post-doping (e.g. iodine or fuming sulfuric acid) have markedly improved the macroscopic properties. Iodine doping, for instance, can increase the tensile strength to ~1 GPa and the electrical conductivity to ~2.9 MS·m^−1^ [30]. More recent pilot-scale integrated processes have yielded fibers with thermal conductivities > 600 W·m^−1^·K^−1^ and electrical conductivities exceeding 20 MS·m^−1^, demonstrating exceptional electromagnetic interference shielding and electrothermal performance.

The utility of CNT fibers is most clearly demonstrated in extreme-environment domains, which impose contradictory requirements: ultralow mass coupled with extreme mechanical and transport resilience [31]. CNT fibers are uniquely positioned to resolve these tensions [32,33]. For example, continuous CNT-fiber laminates have demonstrated a specific tensile modulus (~258 GPa·(g·cm^−3^)^−1^) that competes with high-end T1100G carbon fiber systems [34]. NASA’s validation of large pressure vessels reinforced with CNT fibers confirmed their environmental resilience, with estimated structural mass savings of approximately 30% for launch vehicles [35]. Additionally, CNT-based coatings and engineered surface chemistries help mitigate abrasive planetary dust [36]. On the electrical side, copper/CNT (Cu/CNT) core–shell architectures and high-conductivity cables rival or exceed the specific conductivity of bulk copper at only ~1/6 of the density, making them premier candidates for lightweight power harnesses in aerospace and military systems [37–39]. Beyond their intrinsic structural excellence, CNT fibers serve as a critical performance determinant that governs the hierarchical evolution from individual components to complex architectures. In the subsequent discussion on fiber devices, we articulate how the hierarchical porosity and rapid charge-transfer kinetics of CNT fibers directly dictate the energy density of storage units and the limit of detection in biochemical sensors. This bottleneck hand-over emphasizes that device-level innovation is fundamentally rooted in the material’s interfacial engineering. Their ability to serve as both active functional cores and robust conductive buses facilitates the seamless orchestration of multi-node integrated-fiber electronic systems.

Over the past decade, a synergistic iteration of catalyst engineering, reactor architecture, dope chemistry, and multistep postprocessing, encompassing mechanical drawing, thermal annealing, and chemical doping, has systematically elevated the macroscopic performance of CNT fibers. These coordinated efforts have successfully propelled early sub-GPa tensile strengths into the contemporary multi-GPa specific strength regime, accompanied by significant enhancements in electrical and thermal transport metrics (Fig. 2d and e). Despite these advancements, a significant performance gap persists between macroscopic assemblies and the theoretical limits of individual nanotubes, suggesting substantial room for optimization. This discrepancy is fundamentally rooted in the inefficient property transfer across hierarchical levels, where imperfect packing, structural defects, and weak interbundle interfacial contacts act as critical bottlenecks. Moreover, the lack of a scale-aware process understanding continues to impede the uniformity and throughput required for consistent, large-scale manufacturing.

To bridge these gaps, a paradigm shift toward deterministic, mechanism-driven optimization is needed. At the atomic scale, mechanistic insights into CNT nucleation and chirality control, potentially accelerated by artificial intelligence (AI)-assisted catalyst design, are paramount for reproducibly synthesizing high-crystallinity, low-defect nanotubes. Concurrently, the spinning process must be refined through precise control of dope rheology and densification pathways (including solvent-free consolidation) to reveal conformational transitions during formation and maximize load transfer *via* optimized hierarchical twisting. At the system level, the development of multiscale predictive models linking the tube–bundle–fiber–yarn continuum will enable the rational design of composite architectures that preserve fiber integrity while adding functionality. Ultimately, the establishment of standardized testing protocols and national qualification standards will be indispensable for accelerating the transition of CNT fibers from laboratory prototypes to reliable, industry-grade components in lightweight power harnesses and next-generation smart electronic textiles.

### Graphene fibers

**Figure 3.**
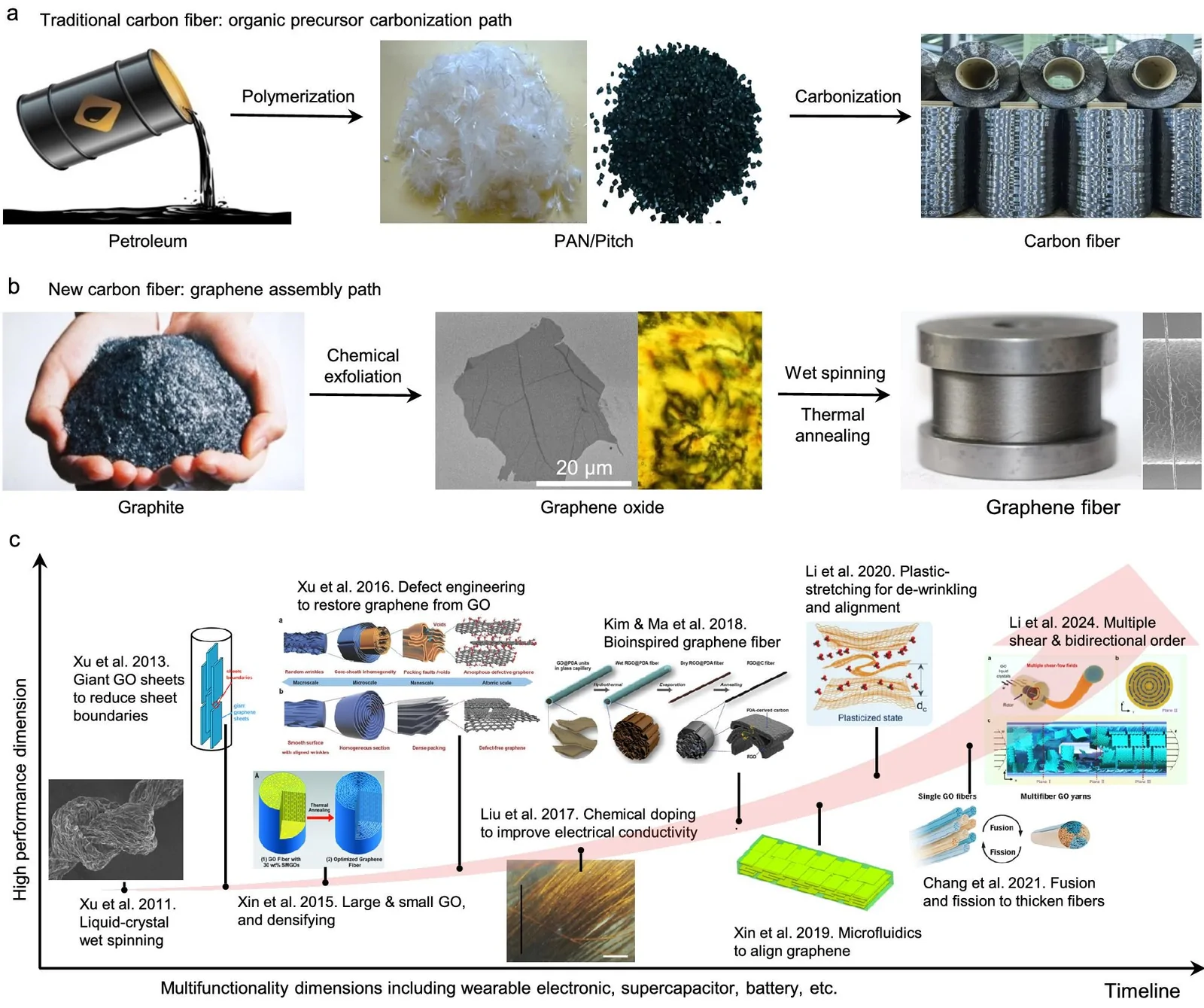
Structural evolution and performance milestones of graphene-based fibers. (a and b) Schematic illustrations contrasting the two primary routes for producing high-performance carbonaceous fibers: (a) the traditional top-down organic precursor carbonization path (e.g. polyacrylonitrile (PAN) or pitch-based carbon fibers); and (b) the emerging bottom-up graphene assembly path, primarily *via* the liquid-crystalline wet-spinning of graphene oxide (GO) followed by chemical or thermal reduction. (c) A comprehensive summary of historical milestones in the enhancement of graphene fiber properties. This panel highlights the critical role of various processing techniques, such as ultra-large graphene oxide sheets, high-draw-ratio spinning, defect engineering, and ultra-high-temperature graphitization, in advancing the limits of mechanical strength, electrical conductivity, and thermal transport. These advancements collectively demonstrate the transition of graphene fibers from early laboratory prototypes to high-performance functional materials that challenge, and in some cases surpass, the performance of state-of-the-art commercial carbon fibers (adapted from [41–44,46–52]).

Graphene fibers (GFs) represent a distinct paradigm in high-performance carbon materials: they are macroscopic 1D filaments assembled directly from 2D nanoscale graphene sheets. By leveraging the exceptional in-plane mechanical, electrical, and thermal properties of graphene, GFs provide a promising pathway to structural-functional fibers that combine robust load-bearing capabilities with superior transport performance. This field was catalyzed by the 2011 discovery of lyotropic liquid-crystalline (LC) behavior in graphene oxide (GO) dispersions (Zhejiang University, China), which established the sheet-to-fiber assembly paradigm, fundamentally departing from traditional polymer‒precursor carbonization routes (Fig. 3a and b) [40,41]. Since the first GFs exhibited tensile strengths of ~140 MPa, research has shifted from simple spinning to elucidating complex multiscale structure–property relationships, specifically, how sheet alignment, interlayer coupling, and defect distribution govern macroscopic behavior [42]. A key mechanistic insight was the intercalation-modulated plasticization in GO fibers, which enables the dynamic rearrangement of sheets during processing, thereby minimizing structural voids [43]. Furthermore, because GFs originate from 2D building blocks, simple longitudinal orientation is insufficient; the development of multiple shear-flow-assisted wet spinning has proven essential for inducing bidirectional ordering and producing dense, highly crystalline GFs [44]. A groundbreaking domain-folding philosophy was presented to construct a highly folded yet densely packed micro-fibrous nanotexture with a tenfold reduction in micro-voids [45].

While liquid-crystal wet-spinning remains the dominant fabrication route, the design space has expanded significantly to maximize intersheet contact and structural ordering [46,52–57]. For instance, the use of GO/DMF (dimethylformamide) liquid crystals with an ethyl acetate coagulation bath has enabled rapid gel filament drying and continuous fiber collection [48,58]. Dope rheology remains a critical determinant of spinnability. The addition of ultrahigh-molecular-weight polymers can increase viscoelasticity and mitigate electrostatic repulsion between GO sheets, thereby enabling high-throughput blow spinning and electrospinning to yield porous, nonwoven fabrics with high specific conductivity [59]. Complementary strategies, including confined hydrothermal assembly, film rolling/twisting, CVD growth on sacrificial templates, and electrospinning of graphene–polymer composites, have further broadened the accessible geometries and porosities [60–63]. Collectively, these methods enable controlled trade-offs among mechanical tenacity, electrical/thermal transport, and processing compatibility.

The refinement of these fabrication routes, coupled with advanced posttreatments, has driven dramatic improvements in GF properties. For example, a tensile strength of up to ~2.2 GPa was reported with a Young’s modulus near 400 GPa. Through an intercalation–plasticization–stretching crystallization protocol, GFs have achieved an orientation degree of ~92% with a crystalline domain size of ~174 nm, leading to a tensile strength of ~3.4 GPa, a Young’s modulus near 400 GPa, an electrical conductivity of ~1.19 × 10^6^ S·m^−1^ and an axial thermal conductivity of ~1480 W·m^−1^·K^−1^ [43,48]. Innovative packing concepts, such as the use of concentric liquid-crystal textures or tailored flake-size distributions, have further pushed thermal conductivities to ~1660 W·m^−1^·K^−1^ [44,47]. Controlling the precise domain folding of graphene has substantially reduced microvoids, resulting in room-temperature-prepared, high-performance graphene-based carbon fibers with a tensile strength of 5.19 GPa [45]. In parallel, chemical doping and intercalation have opened orthogonal pathways to metal-level conductivities and novel quantum phenomena. Electrical conductivities have been driven toward ~2.2 × 10^7^ S·m^−1^, comparable to those of engineering metals on a specific-conductivity basis, thus enabling realistic routes to lightweight power delivery. Remarkably, calcium-intercalated graphene filaments have even exhibited superconducting transitions near 11 K, an unprecedented observation for macroscopic carbon fibers that suggests the potential for flexible, lightweight superconducting cables in aerospace systems [49,64]. These advances are now transitioning toward industrial maturity, with the recent establishment of first-generation, ton-scale filament production lines (1k and 3k counts) with thermal conductivities of approximately 1200 W·m^−1^·K^−1^, marking a pivotal step toward commercial thermal management applications [65].

The combined attributes of GFs enable broad applications ranging from energy harvesting to advanced thermal management [66–70]. For instance, GFs have been successfully integrated into high-capacitance supercapacitors, fiber solar cells, flexible lithium-ion batteries, and moisture-driven harvesters and actuators [51,71–78]. A particularly compelling niche for GFs is in thermal-interface materials (TIMs). Unlike conventional pitch-based carbon fibers, which are often too brittle to optimize contact mechanics, GFs offer a unique combination of high axial conductivity and mechanical compliance [79–82]. GF-based TIMs, aligned *via* electromechanical dual-field strategies, rival metal-level thermal performance while maintaining the interfacial stability required for high-power electronics. Furthermore, GF filament composites have demonstrated exceptional specific thermal conductivities (~363 W·cm³·m^−1^·K^−1^·g^−1^), outperforming traditional pitch-carbon fiber composites and enabling structural-thermal components for complex, sharply curved aerospace packages [83,84]. Together, these processes, microstructure, and chemical advances demonstrate that carefully coupled assembly, compositing, and posttreatment strategies can push graphene fibers into regimes of mechanical and thermal transport performance relevant for high-value structural, thermal management, and electrical applications. In addition, characterized by their exceptional carrier mobility, high specific surface area, and chemical inertness, GFs serve as the quintessential signal transducers within many fiber devices. For instance, their atomic-scale interfaces directly enhance the limit of detection in biochemical sensors and provide ultra-low contact resistance for high-frequency fiber transistors. By mitigating signal attenuation in complex sweat environments, graphene fibers resolve a critical bottleneck for the long-term reliability of integrated-fiber electronic systems. This evolution from intrinsic material properties to device-level performance metrics highlights the pivotal role of graphene fibers in precision healthcare monitoring, serving as the logical starting point for robust, textile-integrated analytical platforms.

Despite these notable achievements (Fig. 3c), the transition of GFs to broad industrial impact remains hampered by unresolved scientific and engineering hurdles. At the fundamental level, sheet-to-fiber assembly introduces unique problems such as wrinkle formation, transverse ordering, and intersheet slip during consolidation. There remains a critical need for multiscale predictive models that capture these coupled phenomena to guide dope engineering. From a processing perspective, the inherent trade-off between alignment, volumetric packing density, and high-throughput production remains a primary bottleneck, often limiting the mechanical robustness of first-generation filaments. Future roadmaps for GFs require a transition toward second-generation fibers that simultaneously achieve multiple strengths and metal-level electrical/thermal conductivities [85,86]. This necessitates coordinated efforts to (1) optimize solvent-free densification strategies that preserve fiber integrity at scale; (2) integrate advanced intercalation chemistries that enhance transport without degrading mechanical tenacity; and (3) establish standardized testing and qualification protocols for fair industrial benchmarking. In short, GFs have evolved from a conceptual paradigm into a rapidly maturing platform. Their ultimate success as high-value electronic products depends on the convergence of process engineering, multiscale structural control, and rigorous standard development.

### Semiconducting fibers

Semiconducting fibers represent a convergence of sophisticated electronic functionality and the inherent mechanical compliance of textiles, establishing a distinct fiber electronic paradigm. Compared with planar thin-film or bulk semiconductors, the 1D fiber geometry offers an exceptionally high specific surface area, enhanced multiaxis bendability, and direct structural compatibility with textile assembly processes. These attributes enable a class of device concepts, ranging from conformal bioelectronic interfaces to interactive garment-integrated sensors, that are fundamentally impractical for rigid or sheet-like electronic platforms. Broadly, the field is bifurcated into inorganic semiconductor fibers and organic (polymeric) semiconductor fibers, each offering complementary strengths: inorganic systems provide high carrier mobility and thermal stability for demanding environmental conditions, whereas organic systems offer solution processability, large-strain compliance, and facile chemical tunability for wearable and bio-integrated applications.

#### Inorganic semiconductor fibers

**Figure 4.**
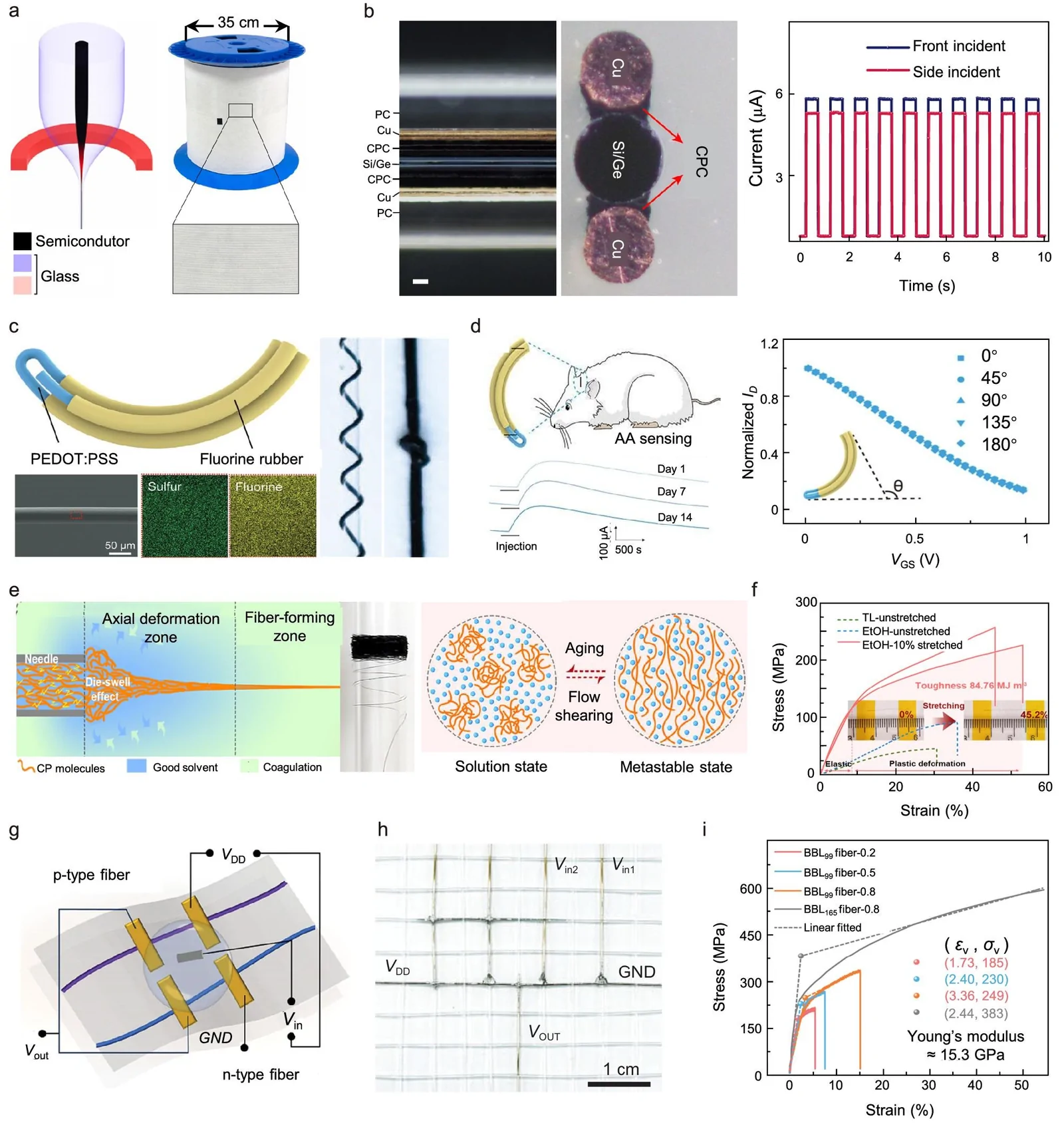
Advancements in semiconducting fiber materials and integrated logic devices. (a and b) Illustration of the melt-core approach for the large-scale production of semiconducting fibers, enabling a pseudo-omnidirectional response with stable sensitivity across different directions (adapted from [2]). (c and d) Application of PEDOT:PSS fibers for real-time *in vivo* ascorbate monitoring in a mouse brain, highlighting the superior bio-interface compatibility and electrochemical sensitivity of organic semiconducting fibers for advanced neuro-monitoring (adapted from [102]). (e and f) Schematic of the FLEX method designed for the continuous and high-fidelity production of organic semiconducting fibers, overcoming the brittleness of traditional organic semiconductors, and allowing the creation of flexible, functional filaments (adapted from [103]). (g) Schematic of complementary inverters constructed using p-type and n-type polymeric fibers, demonstrating the feasibility of building basic logic gates directly within individual fiber-based units (adapted from [104]). (h and i) Development of BBL fibers with an exceptional mechanical strength of up to 600 MPa, which can be seamlessly woven into complex NAND logic circuits, representing a critical step toward achieving textile intelligence and robust computing within everyday garments (adapted from [105]).

Inorganic semiconducting materials (e.g. Si, Ge, SiC, and various metal oxides) are the cornerstone of high-performance electronics due to their superior charge-transport properties. However, traditional obstacles including intrinsic brittleness, slow vapor-phase processing, and catastrophic damage from residual stresses during fabrication, have historically constrained their integration into deformable systems [2,87,88]. Recent breakthroughs in thermal drawing technologies have significantly expanded the feasibility of these materials in 1D forms [89]. For instance, a mechanically aided, thermally assisted drawing process was developed to extend traditional single-material thermal drawing into a sophisticated multi-material process. This innovation effectively mitigates the stress-mismatch and fluid-instability issues that typically plague multi-material preforms during the molten phase. This method enables high-speed drawing and continuous production of Si/Ge semiconductor fibers with lengths exceeding hundreds of meters, resulting in a pseudomagnetic directional response with stable sensitivity regardless of stimulus directions (Fig. 4a and b) [2]. Such advancements reopen the potential for integrating high-performance inorganic logic and sensing into textile-compatible formats. However, the limited large-strain deformability of these inorganic compounds continues to restrict their deployment in high-strain wearable environments.

#### Organic semiconductor fibers

Organic semiconductors, particularly conjugated polymers, are intrinsically amenable to fibrillation due to their solution-processability and inherent mechanical flexibility. Multiple fabrication pathways have been proposed to translate molecular properties into fiber-level performance: electrospinning is typically employed to yield high-surface-area, porous nanofibers suited for ionic–electronic coupling, whereas coating, assembly, and wet spinning provide routes to obtain continuous bulk fibers with tunable internal microstructures [90]. Early electrospinning demonstrations utilizing poly(3-hexylthiophene-2,5-diyl) (P3HT) as a p-type polymer paved the way for nanofiber field-effect transistors [91–95], followed by the development of n-type P(NDI2OD-T2) (a copolymer of naphthalene di-imide (NDI) and bithiophene unit) nanofibers for wearable thermo electrics, which achieved a Seebeck coefficient of −346 μV·K^−1^ [94].

Wet spinning has emerged as the mainstream route for industrial-scale production because of its solvent flexibility and continuous processing capabilities. The 2001–2009 period marked a foundational era, notably with the first report of wet-spun PEDOT:PSS (PEDOT: poly(3,4-ethylenedioxythiophene), PSS: polystyrene sulfonate) fibers, which laid the groundwork for functional-fiber manufacturing [96]. The continuous development of polymer blending, rheology control, and chemical posttreatments has since increased the PEDOT:PSS fiber conductivity to above 3000 S·cm⁻¹. These fibers have enabled a diverse array of device architectures, including organic electrochemical transistors (OECTs), organic thermoelectrics (OTEs), and biosensors [97–101]. Notably, an all-polymer PEDOT:PSS fiber OECT was developed with a mechanical modulus matching that of soft brain tissue. This fiber device resists biofouling and has demonstrated stable, high-sensitivity monitoring of ascorbate in the mouse brain for up to 14 days, revealing its potential for long-term bio-integrated sensing (Fig. 4c and d) [102].

A recently reported process innovation is flow-enhanced crystallization from dilute solutions (FLEX) (Fig. 4e and f). Departing from the conventional reliance on high-concentration dopes to ensure spinnability, the FLEX method exploits dilute solutions where stretching and shear flows promote polymer disentanglement and chain alignment. This mechanism enables the continuous production of organic semiconductor fibers with tensile strengths exceeding 200 MPa [103]. By using the FLEX approach, BBL fibers were produced by overcoming the processing challenges posed by corrosive methanesulfonic acid. The resulting fiber OECTs demonstrated transconductance and μC* figures of merit that surpassed those of their thin-film counterparts while maintaining long-term air stability, a prerequisite for p–n complementary fiber circuitry (Fig. 4g) [104]. In parallel, shear-enhanced liquid-crystal spinning techniques have been implemented to produce poly(benzimidazobenzophenanthroline) (BBL) fibers with mechanical strengths of up to 600 MPa. By integrating these high-strength fibers into woven architectures, researchers have successfully realized NAND logic circuits and programmable logic operations directly within health-monitoring textiles (Fig. 4h and i) [105]. Complementing these advances in p-type materials, optimized wet-spinning and drawing techniques have produced high-conductivity n-type polymer fibers with conductivities reaching ~1600 S·cm^−1^, significantly broadening the material toolbox for fiber-native digital and analog electronics [106].

Research on semiconducting fibers now spans the entire materials–fiber–device chain, yielding an increasingly mature ecosystem in which fiber-based devices, compared with thin-film technologies, demonstrate competitive mechanical robustness and electrical performance. Semiconducting fibers represent the cornerstone of the transition from passive sensing to active logic processing, a leap essential for the system-in-a-fiber paradigm. Bandgap engineering and charge-transport mechanisms directly dictate the on/off ratios and subthreshold swings of fiber transistors, which, in turn, determine the efficiency of fiber-based optoelectronic conversion. By tailoring the semiconducting microstructure, integrated systems can effectively decouple smart textiles from bulky external silicon chips, evolving from simple conductive filaments into autonomous platforms capable of real-time signal orchestration and logic decision-making. Such advances are fundamental to achieving fully decentralized, fiber-native intelligent systems.

Nonetheless, several tightly coupled challenges remain. First, material diversity is currently limited, with research concentrated on a narrow selection of commercial conjugated polymers. There is a critical need for new polymers and hybrid organic–inorganic systems designed specifically for 1D morphologies. Second, the mechanistic understanding of structure–processing–property relationships remains incomplete. Predictive mapping that links spinning parameters to device-level metrics such as charge mobility and ionic–electronic coupling is essential for rational optimization. Furthermore, the 1D geometry poses intrinsic constraints on the device architecture, complicating the implementation of multiterminal and multilayer configurations on a single filament. Many current demonstrations still rely on external supporting substrates, which can undermine the fiber’s native flexibility. Therefore, truly fiber-native solutions are needed, such as substrate-free, coaxial, or multicore architectures. Finally, the field must sharpen its application focus. Rather than attempting to replace conventional silicon-based electronics, research should prioritize problem domains where the fiber form factor offers unique, non-substitutable advantages, such as large-area distributed sensing and minimally invasive bio-interfaces. By aligning targeted materials chemistry with scale-aware processing and fiber-native engineering, semiconducting fibers can evolve into foundational building blocks for the next generation of intelligent, integrated-fiber electronic systems.

### Metal-based fibers

**Figure 5.**
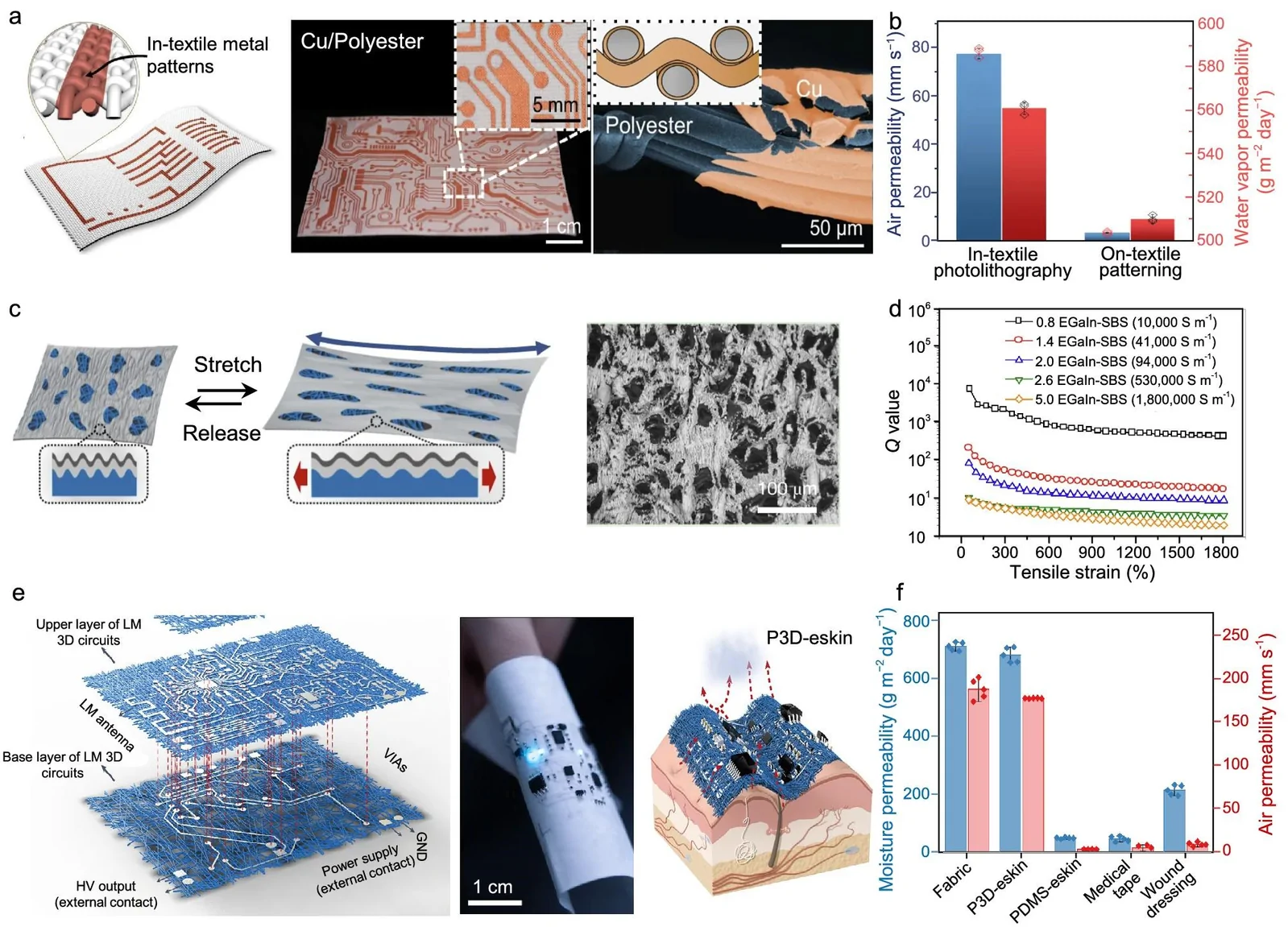
Interfacial engineering and structural design of metal-based fiber materials. (a and b) Detailed view of surface modification and interfacial coordination strategies for metal-based fiber electronic systems. These methods optimize the contact between rigid metallic components and soft textile substrates, ensuring electrical stability under repetitive mechanical strain (adapted from [118,119]). (c and d) Visualization of liquid metal-based textile substrates that maintain high air permeability, addressing the comfort paradox in wearable electronics by allowing gas exchange while maintaining metallic conductivity. (e and f) Strategies for engineering liquid metal-based textile circuit boards. By combining structural hierarchies with the inherent fluidity of liquid metals, these systems achieve high-density interconnects that are resilient to dynamic deformation, providing a reliable platform for air-permeable electronic textile systems (adapted from [112,117]).

Metallic materials have long underpinned the evolution of conventional electronics, offering a combination of exceptional electrical conductivity, chemical stability, and mature processing toolkits that remain unrivaled by most organic or carbon-based alternatives. In the nascent stages of fiber electronics, the integration strategy primarily involved the direct weaving of bulk metallic wires, such as silver (Ag) and copper (Cu), into textile architectures to facilitate basic charge transport or Joule heating [107–109]. While effective for simple heating garments or electromagnetic interference (EMI) shielding, this approach is fundamentally limited by the mechanical mismatch between high-modulus, dense metallic filaments and the ultrasoft, porous nature of textile substrates. Bulk wires often compromise the conformability and tactile comfort of wearable systems, resulting in a stiff, cumbersome user experience. Attempts to mitigate this by using planar thin-film metallization, either magnetron sputtering or thermal evaporation on elastic fibers, have produced stretchable traces. Yet, these configurations remain highly susceptible to microcracking and catastrophic electrical degradation under the cyclic, multi-axis strains characteristic of real-world use [110,111]. The critical transition in the field has thus shifted from simple additive strategies to integrative chemistry, notably through polymer-assisted metal deposition (PAMD) (Fig. 5a and b). This molecular-level engineering facilitates a robust organic–inorganic interface, while the emergence of liquid metals (LMs), such as eutectic gallium–indium (EGaIn), has redefined the design space by marrying metal-level conductivity with the fluid deformability necessary for truly elastic fiber pathways [112–114]. Zhang *et al.* have discovered a new type of metal gel materials-gems that incorporate a high-volume fraction of liquid metal as a fluid within a polymer network [115]. These materials exhibit metal-like electrical conductivity and tissue-like softness, paving the way for soft fiber electronics and related applications [116].

The current taxonomy of metallic fiber technologies comprises three distinct families, each presenting unique trade-offs between transport performance and mechanical resilience. Conventional bulk metal films and microwires occupy the high-performance end of the conductivity spectrum. Still, they are plagued by low fracture strains (typically < 5%) and poor fatigue resistance when bonded to soft, dynamic substrates. Metal–nanowire networks (e.g. AgNWs or CuNWs), often deposited by coating or spray processing, offer a compromise, achieving conductivities in the 10^4^–10^5^ S·m^−1^ range while significantly extending stretchability into the tens-of-percent strain regime. However, the durability of these networks is frequently compromised by weak interfacial adhesion and poor chemical resilience during laundering or exposure to sweat [110]. In contrast, liquid-metal composite fibers have emerged as superior materials for extreme-strain applications. By utilizing pre-strain-assisted self-assembly, microchannel infusion, or co-spinning with elastomers to create porous, percolating conductive architectures, these fibers can deliver conductivities exceeding 10^5^ S·m^−1^ while sustaining unprecedented strains (with isolated reports reaching ~1800%) with minimal resistance fluctuations (<5%). Critically, LMs allow the creation of breathable, interpenetrating networks that preserve high moisture and air permeability (~724 g·m^−2^·day^−1^), thereby resolving the long-standing trade-off between metallic functionality and the physiological requirements of wearable textiles (Fig. 5c and d) [117].

Within this roadmap, metal-based fibers, including emerging liquid-metal composites, serve not merely as conductors but also as indispensable energy buses and flexible joining hubs for complex systems. The high electrical conductivity of metallic fibers is foundational for high-power actuation and high-gain antenna performance. More critically, the mechanical toughness and stretchability explored in previous research directly address the interconnection reliability bottleneck, which may be prioritized in integrated-fiber electronic systems. By establishing low-impedance, mechanically matched electrical junctions at the system level, metal-based fibers ensure seamless power and data distribution across multi-node architectures. This trajectory, moving from ohmic contact optimization to systemic robustness, provides the underlying technical guarantee for resolving the persistent failures of intelligent textiles under rigorous washing cycles and extreme dynamic deformations.

Despite these advancements, the transition from laboratory prototypes to industry-grade components is hindered by four interrelated technical bottlenecks rooted in interfacial and structural mismatch [120]. First, the mechanical property mismatch between high-modulus metals and soft-fiber substrates induces localized stress concentrations, which manifest as cracking and delamination [121]. Addressing this requires the development of compliance-matching hybrids, such as ultrathin metal–nanosheet/fiber composites or LM–polymer blends [122,123]. Second, the inherent porosity and surface roughness of textile fibers complicate the formation of stable chemical bonds. To overcome this, PAMD and similar molecular grafting techniques enable guided *in situ* reduction of metal ions, resulting in tough nanocomposite interfaces with significantly enhanced shear strength and fatigue resistance (Fig. 5a) [118,119,124–128]. Third, the blockage of inter-fiber pores by continuous metal coatings often leads to a complete loss of breathability, a challenge now being addressed through hierarchically porous mesh designs and liquid–metal–elastomer interpenetrating networks (Fig. 5e and f) [112,129–131]. Finally, the challenge of high-precision patterning on rough, 1D geometries remains. Substrate-adaptive manufacturing, including laser direct writing and transfer printing, as well as self-sintering conductive inks, is essential for defining the complex, high-density traces required for modern integrated fiber circuits [132].

To accelerate the maturation of metal-based fiber electronics, a strategic roadmap focusing on scalability, reliability, and high-value applications is needed. Priority must be given to the development of integrated, continuous metallization platforms, such as roll-to-roll micro-patterning and in-line liquid-metal filling, to ensure uniform and low-cost production. Concurrently, the community must establish a multi-scale reliability framework that leverages multi-physics modeling and machine learning analysis to decode coupled mechanical–electrical failure mechanisms during dynamic use, providing the lifetime prediction tools necessary for industrial qualification. Furthermore, the field should pivot toward application-driven demonstrations in high-value, niche domains where the fiber form factor provides non-substitutable advantages. Examples include ultrahigh-density fiber electrodes for brain–machine interfaces and implantable wireless sensing fibers for deep-tissue monitoring. Such applications not only validate system-level performance but also create the commercial pull-through necessary to standardize manufacturing and testing protocols. By aligning advancements in interfacial chemistry with scale-aware processing and robust reliability frameworks, metal-based fibers will evolve into indispensable electrical backbones for the next generation of multifunctional, intelligent textile systems.

### Ceramic fiber materials

Within the broad family of ceramic fibers, continuous high-temperature ceramic fibers produced *via* polymer-derived ceramic (PDC) and sol–gel routes constitute a mature branch that has long served as the structural workhorse of high-temperature engineering [133,134]. Non-oxide systems epitomized by polycarbosilane-derived silicon carbide (SiC) fibers, successively the Nicalon, Hi-Nicalon, Hi-Nicalon Type S, Tyranno SA, and Sylramic generations, have evolved from oxygen-rich Si–C–O compositions toward near-stoichiometric SiC, raising their creep-rupture limits to ~1400°C and Young’s moduli up to ~420 GPa [133]. In parallel, oxide fibers such as the 3M Nextel 610/650/720 family combine α-Al_2_O_3_-based microstructures with mullite-pinning second phases to deliver high-temperature strength retention and oxidation stability [134]. A defining technological feature of these continuous ceramic fibers is that their multifilament tows can be plain-woven, satin-woven, braided, or 3D-orthogonally interlocked into near-net-shape preforms that, after matrix infiltration, yield ceramic-matrix composites (CMCs) for aero-engine hot-section components (turbine shrouds, nozzles, combustor liners) and hypersonic thermal-protection structures [135].

Inorganic nanofibers, broadly encompassing carbon and ceramic variants, constitute a class of high-performance materials that uniquely synergize the thermal and chemical stability of inorganic solids with advantageous size effects (e.g. surface dominance and quantum confinement) at the nanoscale. The fundamental values of these fibers lie in their high aspect ratio and structural continuity, which provide a geometric bridge between molecular-level properties and macroscale architectures. These synergistic attributes make inorganic nanofibers indispensable for applications in national defense, energy electronics, and transportation, where organic polymers fail due to thermal or chemical limitations [31,33]. While various fabrication methods, such as chemical vapor deposition and solution blowing, have been explored, electrospinning remains the preeminent route due to its exceptional compatibility with precursors and its ability to achieve ultrafine, uniform diameters at high throughput [136].

**Figure 6.**
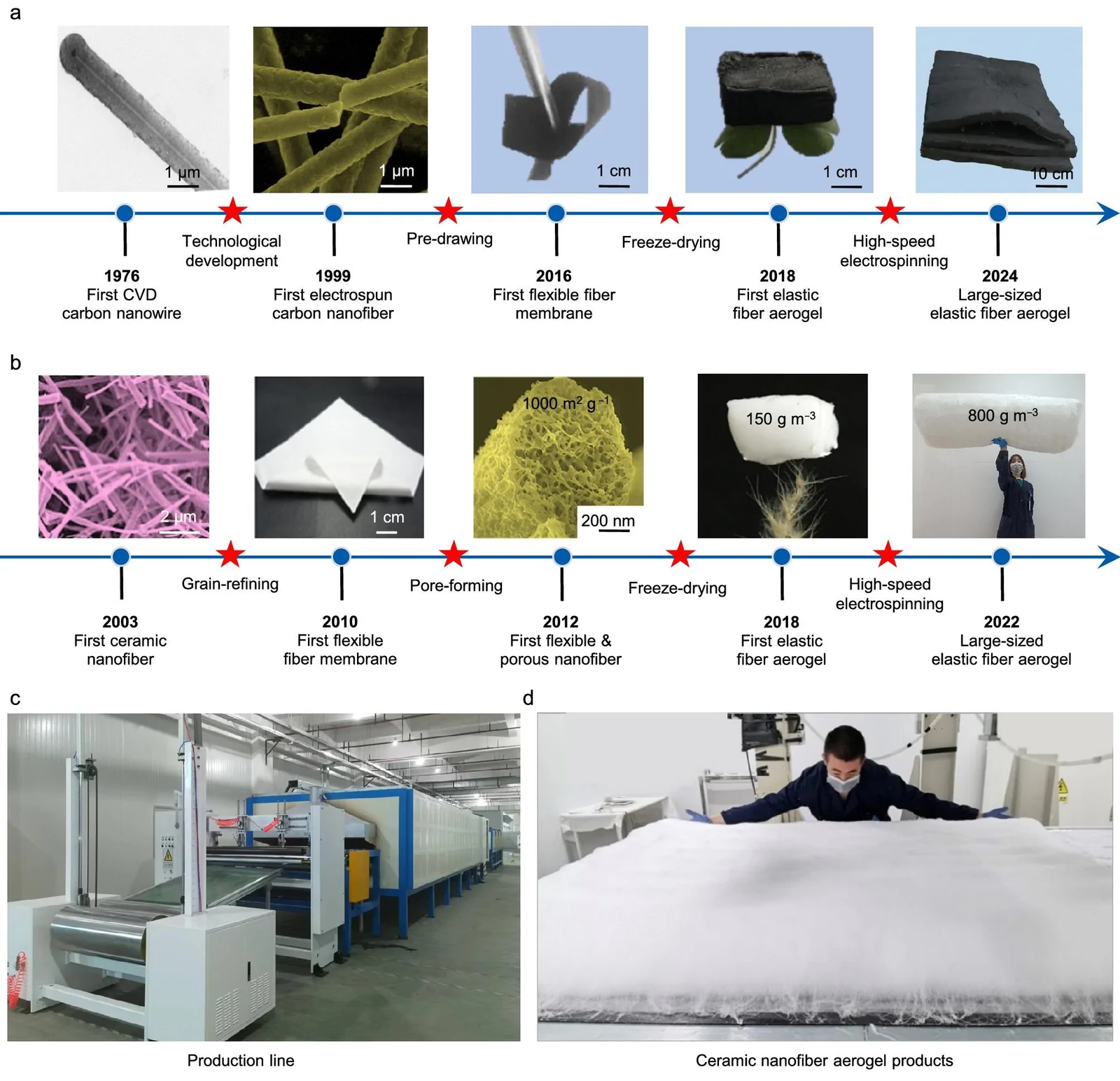
Evolution and industrial scale-up of inorganic nanofiber materials. (a and b) Historical and technological progression of carbon-based and ceramic-based nanofibers, illustrating the transition from basic structural reinforcement to multifunctional inorganic systems capable of operating in extreme thermal and chemical environments. (c and d) Overview of a scale-up production line for inorganic nanofibers, alongside examples of large-sized, high-quality end products, emphasizing the maturation of electrospinning techniques.

The evolution of carbon nanofibers (CNFs) illustrates a paradigm shift from simple filtration membranes to complex 3D elastic monoliths. Early efforts focused on stabilizing and high-temperature carbonizing polyacrylonitrile (PAN) to yield continuous CNFs (Fig. 6a) [137]. While these early membranes served well for energy storage and filtration [138,139], their utility was often confined to 2D formats. A significant breakthrough occurred with the development of porous carbon aerogels and, more recently, the in-flight self-entangling assembly reported in 2024 [140]. This innovation enables the continuous production of elastic CNF aerogel networks, representing a departure from traditional batch-processed, brittle aerogels. By transitioning from disordered membranes to ordered, elastic 3D networks, the field has unlocked practical routes for electromagnetic shielding and advanced thermal management, demonstrating that structural design at the fiber-assembly level is as critical as the carbonization chemistry itself.

The trajectory of ceramic nanofibers reveals a similar struggle to overcome the intrinsic brittleness (Fig. 6b) [141–143]. Since the synthesis of the first SiO_2_–TiO_2_ nanofibers in 2003, researchers have employed diverse microstructural strategies, such as densification control, ionic doping, and grain boundary engineering, to increase the flexibility of inherently rigid oxide systems [144–148]. To address scale and mechanical resilience, a one-step fiber-curling molding and topological-assembly route was introduced in 2022 to produce mullite (3Al_2_O_3_·2SiO_2_) nanofiber aerogels that combine compressive and tensile recovery, overcoming earlier bottlenecks in the low-cost, continuous bulk production of ceramic nanofiber monoliths (Fig. 6c and d) [149]. However, a longstanding chemistry-level bottleneck in polymer-assisted electrospinning is the decomposition of organic binders during calcination, which inevitably results in structural defects that severely limit single-fiber strength [150]. The most profound advancement in this domain is the polymer-free synthesis strategy. By utilizing inorganic–alkoxide inert coordination followed by linear polycondensations, researchers have synthesized linear inorganic macromolecules with metal–oxygen repeat units. This approach avoids the defect-inducing decomposition phase, yielding densified ceramic nanofibers with record-breaking mechanical properties. For example, Al_2_O_3_ single nanofibers produced *via* this route achieved a tensile strength of 1.02 GPa even after calcination at 1700°C, nearly four times that of commercial 3M Nextel® micron-scale fibers [151–153]. This transition from filler-grade to load-bearing nanofibers is foundational for the establishment of the first industrial production lines for flexible/elastic ceramic nanofibers.

Building on these foundations, several recent advances from 2024 to 2026 have substantially expanded the mechanical-elasticity envelope of ceramic nanofibers, moving the field beyond the long-standing brittleness barrier toward fatigue-resistant, repeatedly deformable, and multifunctionally integrated behavior at the single-fiber level. A coaxial electrospinning strategy has been developed in which a non-spinnable, low-viscosity sol (<10 mPa·s) consisting only of ceramic precursors and solvents is encapsulated inside a polymeric shell, forming core–shell precursor fibers that are subsequently calcined into ceramic fibers with reduced porosity, decreased surface defects, uniform crystal packing, and controlled diameters. This approach has been demonstrated across a wide range of compositions, including TiO_2_, ZrO_2_, SiO_2_, and Al_2_O_3_. Most strikingly, normally-fragile polycrystalline TiO_2_ nanofibers acquire excellent flexibility, while single-component ZrO_2_ nanofibers display markedly enhanced toughness; the same approach further enables the creation of ceramic micro- and nano-springs, whose fiber assemblies constructed from helical fibers exhibit a density-normalized toughness 3.5–5 times that of straight fibers, attributed to improved fracture strain [154]. Through a distinct mechanistic route, a carboxylic-ligand confinement strategy combining formic and acetic acids has been employed to regulate inorganic colloidal growth in α-Al_2_O_3_ nanofibers, where silica- and boron-oxide-induced local disorder around the α-Al_2_O_3_ grains imparts excellent mechanical properties and flexibility. The resulting nanofibers withstand 500 buckling cycles without fracture and retain their flexibility across the wide temperature window of −196 to 1100°C, demonstrating sustained mechanical robustness under extreme thermomechanical conditions [155]. Most recently, this strong ligand-coordination strategy has been extended to multi-component systems, where reactive metal precursors stabilized by carboxylic acids form a homogeneous multiphase ceramic nanofiber: with a buffering zirconia phase between the alumina skeleton and infrared-reflective titanium oxide species, the resulting nanofibers simultaneously achieve mechanical robustness, outstanding thermal insulation, and infrared reflection within a single fiber building block [156]. Together, these advances mark a clear paradigm shift, in which elastic ceramic nanofibers are no longer mere passive insulating elements but are now mechanically deformable, fatigue-resistant, and multifunctionally integrated structural components for next-generation extreme-environment applications.

Building on these mechanical and chemical foundations, inorganic nanofibers have transitioned from laboratory curiosities to critical components in extreme-environment systems [136]. Their ultrahigh porosity and superior refractory properties make them ideal for thermal protection in hypersonic vehicles and acoustic stealth in advanced aircraft [157–162]. In the realm of energy, flexible ceramic membranes have redefined safety standards for lithium-ion batteries by suppressing dendrite growth and mitigating thermal runaway [163,164]. Furthermore, refractory fibers such as SiO_2_ act as white activated carbon, offering regenerable sorbents that can withstand corrosive, high-temperature filtration cycles that incinerate conventional carbon fibers [165]. These applications highlight a clear evaluative trend: the value of inorganic nanofibers is not just in their nano size but in their ability to maintain functional integrity under physiological or mechanical conditions where all other material classes fail.

Despite this maturity, three tightly coupled challenges define the future research frontiers of inorganic nanofiber materials. First, while electrospinning is versatile, accessing the ultrafine regime (diameters ≤30 nm) remains difficult. At this scale, size-dependent strengthening and transport effects become dominant, yet achieving such diameters within high-throughput platforms requires novel strategies to stabilize fluid jets. Second, the predictive mapping of grain-boundary engineering to tensile toughness is still in its infancy. More sophisticated calcination pathways are needed to maximize single-fiber strength for structural applications. Third, the scalability of 3D assembly remains the ultimate industrial hurdle. Translating single-fiber records into cost-effective, continuous production of bulk monoliths is essential to move beyond niche defense applications. Addressing these challenges through the convergence of inorganic macromolecular chemistry and scale-aware manufacturing will be a catalyst that transforms inorganic nanofibers into a cornerstone of next-generation structural and functional engineering.

### Novel fiber materials

The contemporary landscape of fiber electronics is predominantly defined by the established material families (e.g. polymeric, carbon-based, and metallic fibers), which serve as the structural and functional bedrock for smart textile systems. However, an analysis of these materials reveals a fundamental performance-compliance trade-off that limits the ceiling of fiber-native devices. Conventional polymers offer exceptional processability and mechanical flexibility but lack intrinsic electronic or electrochemical activity. Carbon nanotubes and graphene fibers exhibit impressive strength and conductivity, but often struggle with functional extensibility and limited redox-active sites. Conversely, while metal microwires provide superior conductivity, their high density and inherent stiffness lead to mechanical fragility and poor conformability under the complex deformations required for wearable interfaces [166]. Overcoming this long-standing compromise demands a transition from additive strategies, where active fillers are merely dispersed within passive matrices, to a molecular-integration paradigm. The objective is to engineer fiber materials that inherently possess a polymer-like rheology and compliance alongside metal-like electronic functionality. Metal-backboned polymers (MBPs) have recently emerged as a transformative solution to this challenge, effectively bridging the conceptual divide between organic macromolecules and inorganic metals at the molecular level [167].

**Figure 7.**
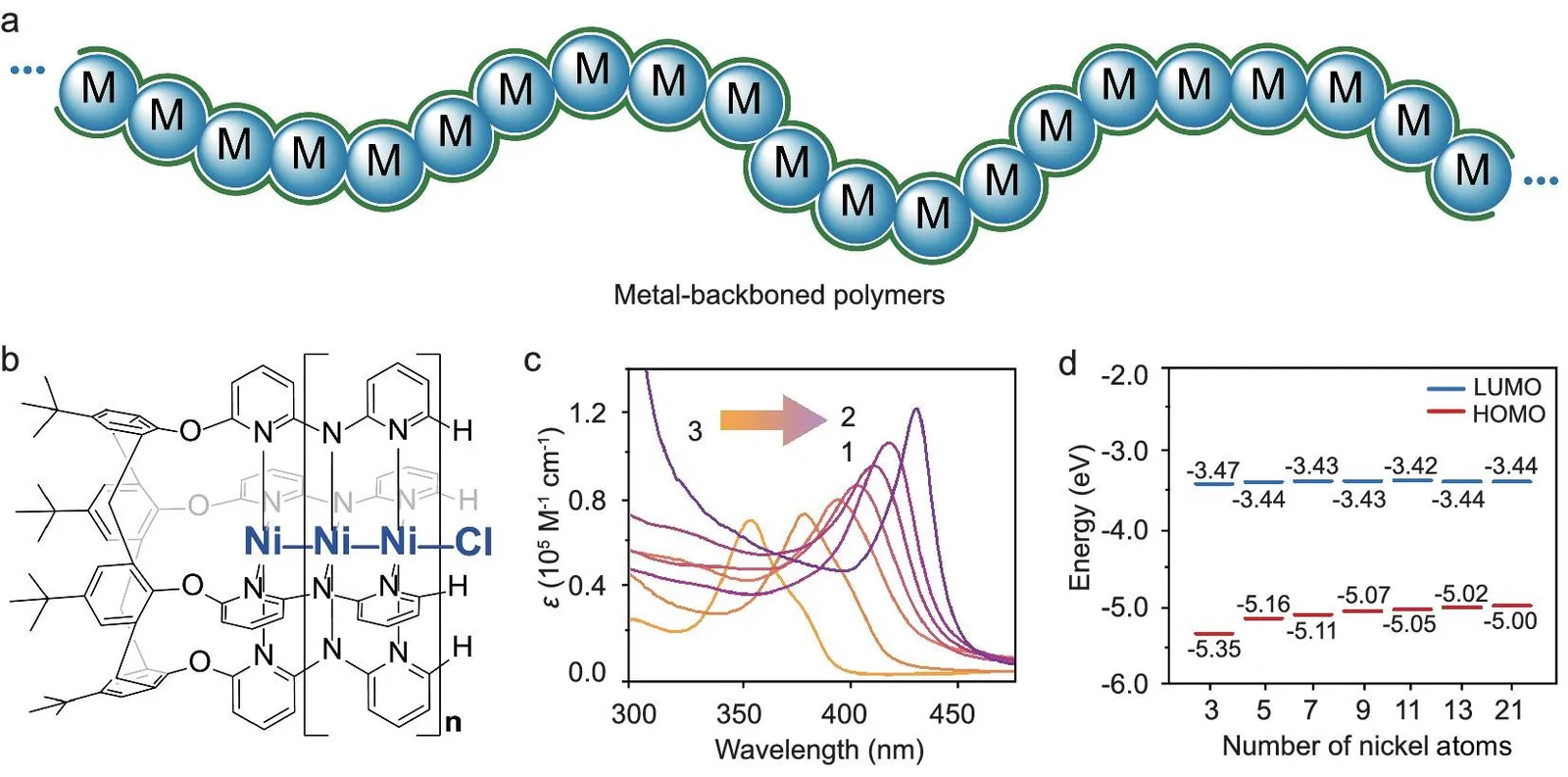
Molecular engineering and property tuning of metal-backboned polymers. (a) Structural illustration of a metal-backboned polymer (MBP), where M represents a metal atom integrated into the polymeric chain. This unique architecture combines the electronic properties of metals with the processing flexibility of polymers (adapted from [170]). (b) Specific molecular structure of nickel-based MBPs, showcasing the precise coordination chemistry that governs the material’s backbone (adapted from [168]). (c and d) Optical and electrochemical profiles of nickel-based MBPs, demonstrating how the electronic energy levels and electrochemical response can be finely tuned by adjusting the length of the nickel backbone, thereby providing a molecular-level design tool for creating customized electronic fiber materials (adapted from [170]).

The defining characteristic of MBPs is a primary chain formed by metal atoms linked through delocalized one-dimensional metallic bonding, facilitated by sophisticated orbital overlap (Fig. 7a). Unlike traditional coordination polymers, where metal ions are isolated by organic spacers, the metal–metal distances in MBPs are shorter than the sum of the van der Waals radii of isolated metal atoms, creating continuous charge-transport channels. This architecture allows the backbone to serve as a molecular wire, while conformationally flexible organic ligands ensure structural integrity and solution processability. The intrinsic value of MBPs lies in their immense design space. By modulating metal identity, ligand chemistry, and coordination geometry, researchers can program electronic bandgaps, carrier delocalization, and redox activity with atomic precision [168,169]. For instance, structure–property evaluations in nickel-based systems have demonstrated that increasing the chain length and molecular ordering lead to pronounced bathochromic shifts in the optical absorption spectrum and a systematic narrowing of the effective bandgap (Fig. 7b–d). This molecular-level tunability indicates that MBPs are not merely passive conductors but also active semiconductors or electrocatalytic platforms whose electronic characteristics can be tailored to surpass the limits of traditional inorganic or polymeric fibers [170,171].

From a manufacturing perspective, the solution processability of MBPs offers a distinct advantage for scalable fiber production through established wet-spinning and thermal-drawing platforms. The true engineering challenge, however, lies in preserving molecular-level order during the transition to macroscopic fiber forms. Under controlled shear and extensional flows during spinning, MBP chains can be highly aligned along the fiber axis. This process is critical for maintaining continuous axial charge-transport pathways in the solid state. This oriented assembly minimizes electric field distortion during mechanical bending, a property of immense value for wearable energy textiles and high-sensitivity sensors. Furthermore, the functionality of MBP fibers can be enhanced through targeted post-processing strategies, such as hierarchical twisting, controlled pore induction, and chemical annealing, thereby strengthening interchain electronic coupling and improving mechanical robustness [172]. By treating the fiber as a hierarchical assembly of molecular wires rather than a bulk homogeneous material, MBPs enable a new class of active fibers capable of high-density energy storage, optoelectronic conversion, and even neuromorphic computing.

Despite this extraordinary potential, MBP fibers are currently in their exploratory infancy and face multi-dimensional challenges that prevent immediate industrial adoption. The primary bottleneck is synthetic. Current routes are often condition-sensitive and complex, making it difficult to achieve the high molecular weight and metal-site purity required for high-performance devices. To move forward, the community must transition from trial-and-error synthesis to a computational-design framework, utilizing machine learning and coordination kinetics modeling to predict and enforce specific metal-bond topologies. Additionally, while MBPs provide exceptional axial transport, their system-level performance is highly dependent on interfacial engineering with electrodes and synergistic integration with other conductive fillers such as carbon nanotubes or graphene. Such hybrid strategies can enhance percolative transport and mechanical toughness without disrupting the molecular order of the MBP backbone. Finally, rigorous longitudinal assessments of environmental stability and fatigue resistance are essential. If these synthetic and engineering hurdles can be overcome through convergent research in coordination chemistry and fiber-native processing, MBPs will establish a third axis in the materials library, providing the long-sought synergy of flexibility, conductivity, and multifunctionality for the next generation of integrated fiber electronics.

**Figure 8.**
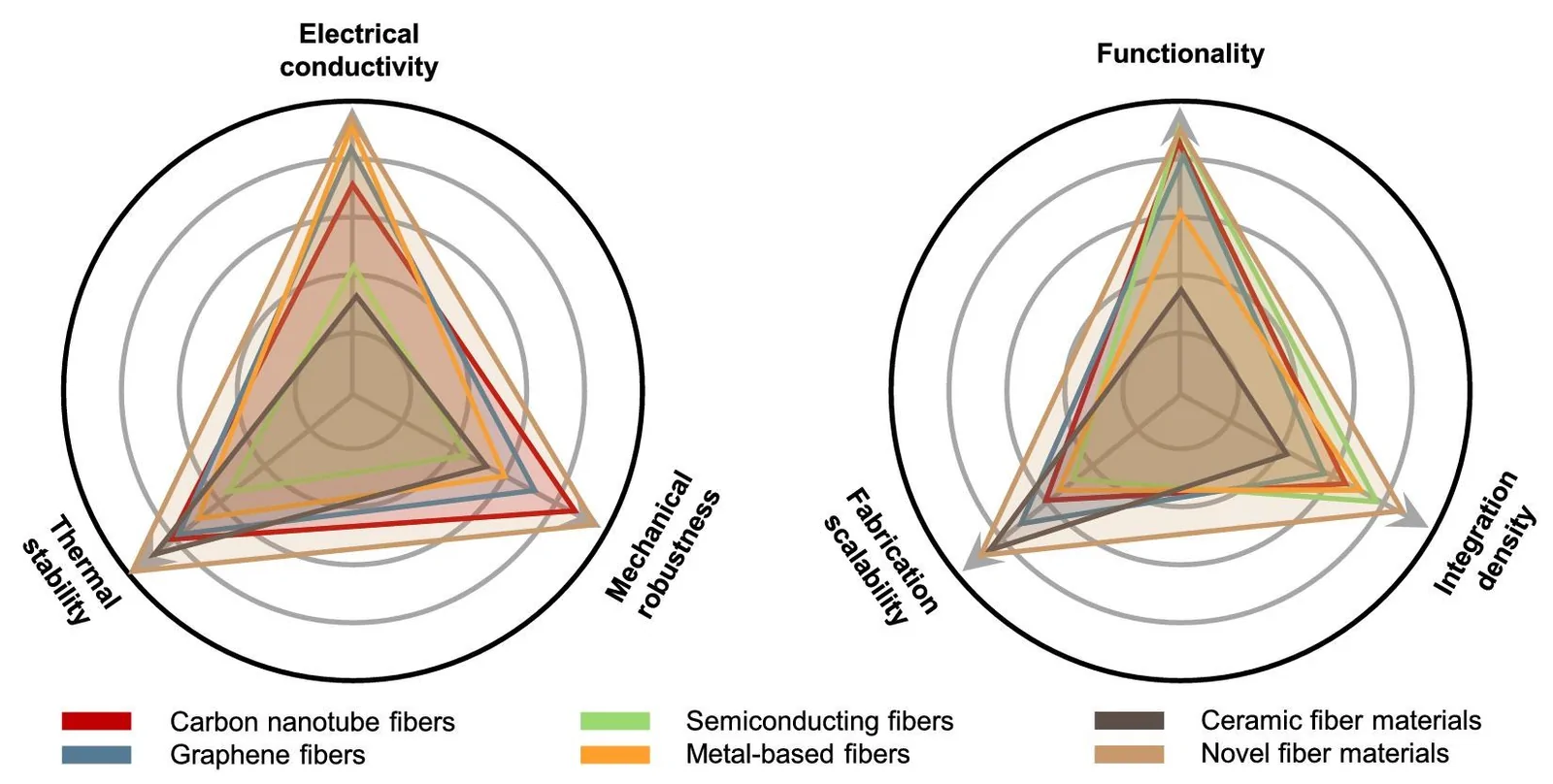
Multi-dimensional performance benchmarking and trade-off analysis of functional fiber materials. The left panel compares electrical conductivity, mechanical robustness (encompassing strength and stretchability), and thermal stability. The overlapping envelopes highlight the complementary nature of these materials, such as the extreme conductivity of metal fibers versus the balanced electro-mechanical profiles of carbon-based filaments. The right panel presents an evaluation based on functional diversity, integration density, and fabrication scalability.

In short, the diverse material portfolio established in this section, ranging from carbon-based nanomaterials and semiconductors to metals, inorganic nanofibers, and novel materials and structures, constitutes the fundamental toolbox for the hierarchical evolution of fiber electronics. To quantitatively evaluate these candidates, we provide a multi-dimensional benchmarking of material families across intrinsic physical properties and systemic integration metrics (Fig. 8). These radar plots reveal the inherent performance trade-offs that necessitate a shift from single-property filaments toward a paradigm of heterogeneous material orchestration. Specifically, the high carrier mobility and exceptional interfacial sensitivity of carbon nanotubes and graphene can be leveraged to raise the performance ceiling of fiber-sensing and energy-storage devices, among others. The fabrication and bandgap engineering of semiconducting fibers enable the transition from passive components to active logic and fiber-native computing. Furthermore, the high-conductivity, stretchable metallic/liquid-metal networks, combined with the environmental resilience of ceramic and novel fibers, provide the necessary structural and electrical backbone for the system-level integration of fiber devices. These materials collectively address the most pressing practical challenges, namely the interconnection reliability, washability, and long-term stability, transforming discrete components into robust, autonomous integrated systems.

## FIBER DEVICES

Building upon the material foundations established in the previous chapter, this chapter provides a comprehensive review of the principal functions realized in fiber device platforms (e.g. energy harvesting and storage, physical and chemical sensing, light emission and display, and neuromorphic computing), linking each functional class to its underlying material compositions, innovative device architectures, and specialized fabrication strategies. A central focus is placed on how the unique one-dimensional, curved form factor of fibers fundamentally reshapes device physics, scaling laws, and manufacturing processes, presenting distinct engineering challenges in curved-surface deposition, interface stability, continuous manufacturing, and durable encapsulation. For each functional category, we systematically summarize representative device demonstrations and benchmark their performance against real-world application requirements. Furthermore, we critically outline potential pathways and necessary innovations to bridge the gap between compelling laboratory proofs of concept and the creation of robust, textile-compatible products, thereby mapping the trajectory from fundamental device principles to integrated system-level applications.

### Energy-conversion fiber devices

**Figure 9.**
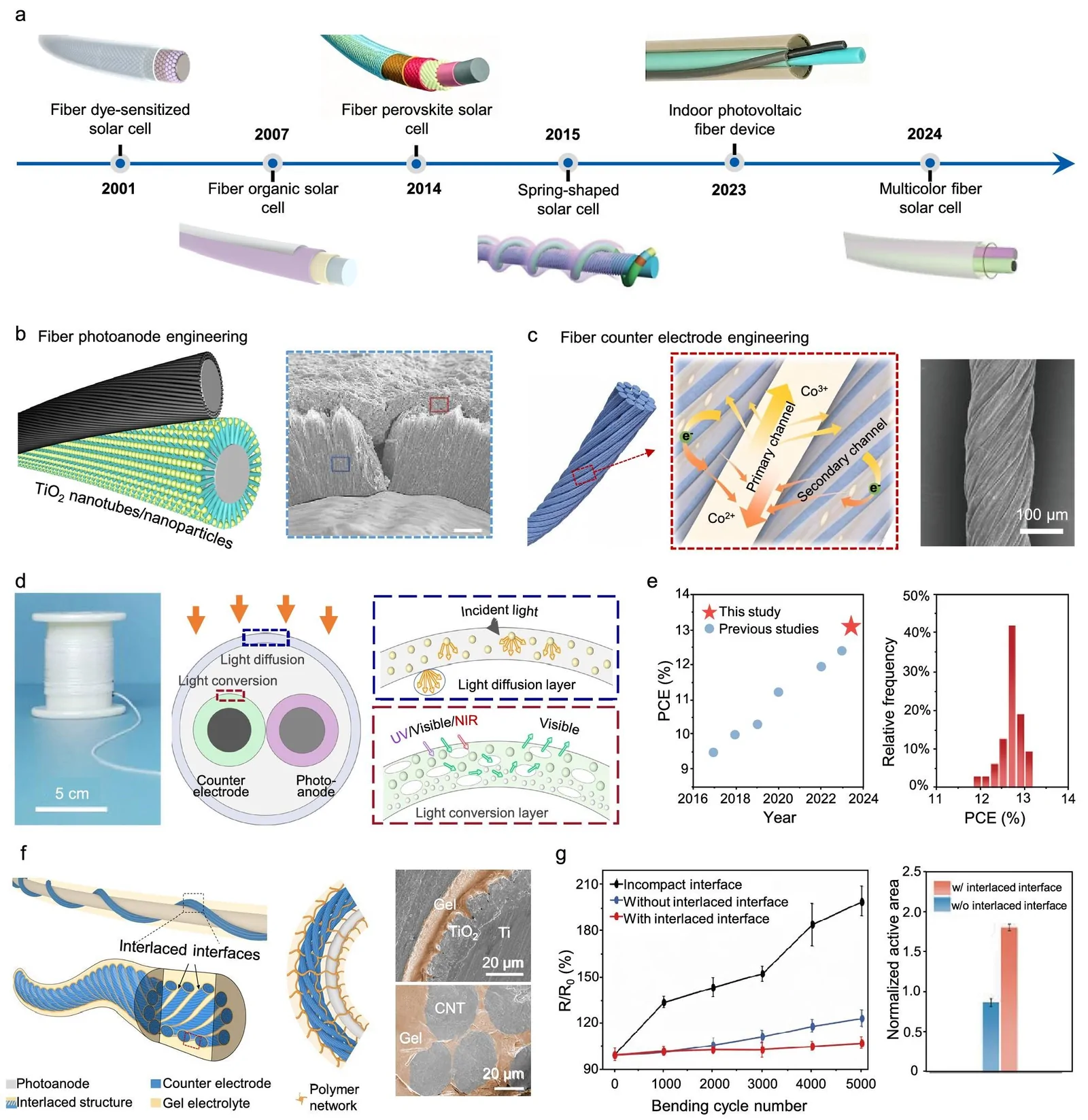
Strategic evolution and performance optimization of fiber solar cells. (a) A comprehensive overview of the trajectory of fiber solar cells, tracing the evolution from early dye-sensitized prototypes to high-efficiency perovskite and organic architectures, highlighting the transition from laboratory curiosities to flexible, wearable power-generating units (adapted from [175,177–180,185]). (b–g) Specific methodologies for optimizing fiber-shaped dye-sensitized solar cells, including (b) photoanode optimization to enhance surface area and light harvesting (adapted from [175]), (c) counter-electrode architecture optimization using nanomaterial coatings to improve charge collection efficiency (adapted from [183]), (d and e) structural design (e.g. coaxial vs. twisted geometries) to balance flexibility with active-area exposure (adapted from [185]), and (f and g) the *in situ* synthesis of gel electrolytes, which addresses the critical challenges of solvent leakage and long-term environmental stability in wearable formats (adapted from [173]).

Fiber solar cells represent a fundamental departure from the planar photovoltaic paradigm, offering a unique combination of low areal mass, multi-axial mechanical compliance, and seamless compatibility with large-scale textile assembly. Unlike traditional rigid or flexible thin-film cells, the 1D geometry allows light harvesting from 360°, making them ideal for distributing power generation in wearable electronics [173–176]. The evolution of this field has been driven by three primary material families: dye-sensitized solar cells (DSSCs), organic solar cells (OSCs), and perovskite solar cells (PSCs). The historical trajectory of these devices reveals a steady increase in power conversion efficiency (PCE), starting with the 2001 nascent demonstration of coaxial DSSCs with limited open-circuit voltages (~0.30–0.35 V) (Fig. 9a) [177]. OSCs were developed in 2007, utilizing a coaxial multilayer stack which has since seen efficiency increase from an initial value of 1.1% to the best-reported value of approximately 9.4% [174,178]. PSCs, the most recent addition, have demonstrated the most rapid progress. Since their 2014 debut, with a PCE of 3.3%, innovations in elastic architectures, such as spring-shaped titanium anodes and 2D–3D hybrid perovskite absorbers, have increased the efficiency to 11.96% while maintaining remarkable ambient stability [179–181].

The rapid advancement of fiber DSSCs, in particular, serves as a master class in electrode and interface engineering under 1D constraints. The primary challenge has been to maximize the charge collection efficiency on a curved, limited surface area. To address this, researchers have transitioned from simple metallic wires to hierarchical nanostructures, such as TiO_2_ nanotube arrays grown directly on Ti wires, which facilitate rapid interfacial charge transport [182]. Further refinements, including the embedding of TiO_2_ nanoparticles within V-shaped nanotube channels, have an optimized surface area for high-performance indoor energy harvesting (Fig. 9b) [175]. On the counter electrode side, the development of hierarchically porous CNT fibers provides abundant catalytic sites and open ionic channels for accelerated electrolyte diffusion (Fig. 9c) [183]. Moreover, the introduction of polar-polymer interlayers for interface passivation has proven effective in suppressing electron recombination and increasing dye loading, thereby increasing the photocurrent. These electrode-centric strategies highlight that the performance ceiling of fiber photovoltaics is dictated not only by active materials but also by the architectural efficiency of the charge-extraction interfaces [184].

Beyond internal transport, the unique optical physics of the fiber form factor offer opportunities for light management that are unattainable in planar configurations. By exploiting the 360° illumination capability of the fibers, researchers have integrated light-scattering and wavelength-conversion layers to broaden the utilization of the solar spectrum, achieving a record PCE of 13.11% while simultaneously enabling multi-color appearances for aesthetic textile integration (Fig. 9d and e) [185]. Parallel to these optical gains, the field has aggressively addressed the safety and operational stability of these devices. The transition from volatile liquid electrolytes to quasi-solid or gel formulations is a critical milestone for wearable applications. *In situ* synthesis techniques, in which polymer gel electrolytes are polymerized within porous fiber electrodes, have produced mechanically interwoven electrode–electrolyte interfaces. This interlocked architecture not only mitigated leakage risks but also preserved rapid ion transport, with gel-based DSSCs achieving PCEs of 7.95%-8.58% (Fig. 9f and g) [173,183,186–188]. This shift toward solid-state and quasi-solid-state systems underscores a broader evaluative trend: for fiber electronics to succeed, wearability and safety must be engineered with the same rigor as efficiency.

Despite these remarkable laboratory milestones, the transition from centimeters to industrial-scale kilometers remains hindered by three interrelated technical bottlenecks. First, the curvature problem persists. Conventional planar deposition techniques often fail to produce uniform functional layers on high-curvature surfaces, leading to interfacial defects and performance degradation [189,190]. This necessitates the development of fiber-specific photoactive inks and substrate-adaptive deposition processes that can precisely control wetting and drying on cylindrical geometries [191]. Second, operational stability under the dynamic mechanical and environmental stresses of daily wear, including humidity, oxygen exposure, and repeated bending, is currently inadequate, with most studies lacking standardized long-term testing protocols [192–195]. Third, the scale-up of production requires a move toward continuous roll-to-roll manufacturing, which imposes strict demands on ink viscosity and the mechanical durability of the deposited films during processing [196–199]. Moving forward, the industrialization of fiber photovoltaics will require coordinated convergence among fiber-tuned material formulations, automated deposition platforms, and community-agreed durability standards. Only by aligning these three pillars can we transform high-performance filaments into robust, energy-harvesting backbones for next-generation smart textiles.

### Energy storage fiber devices

**Figure 10.**
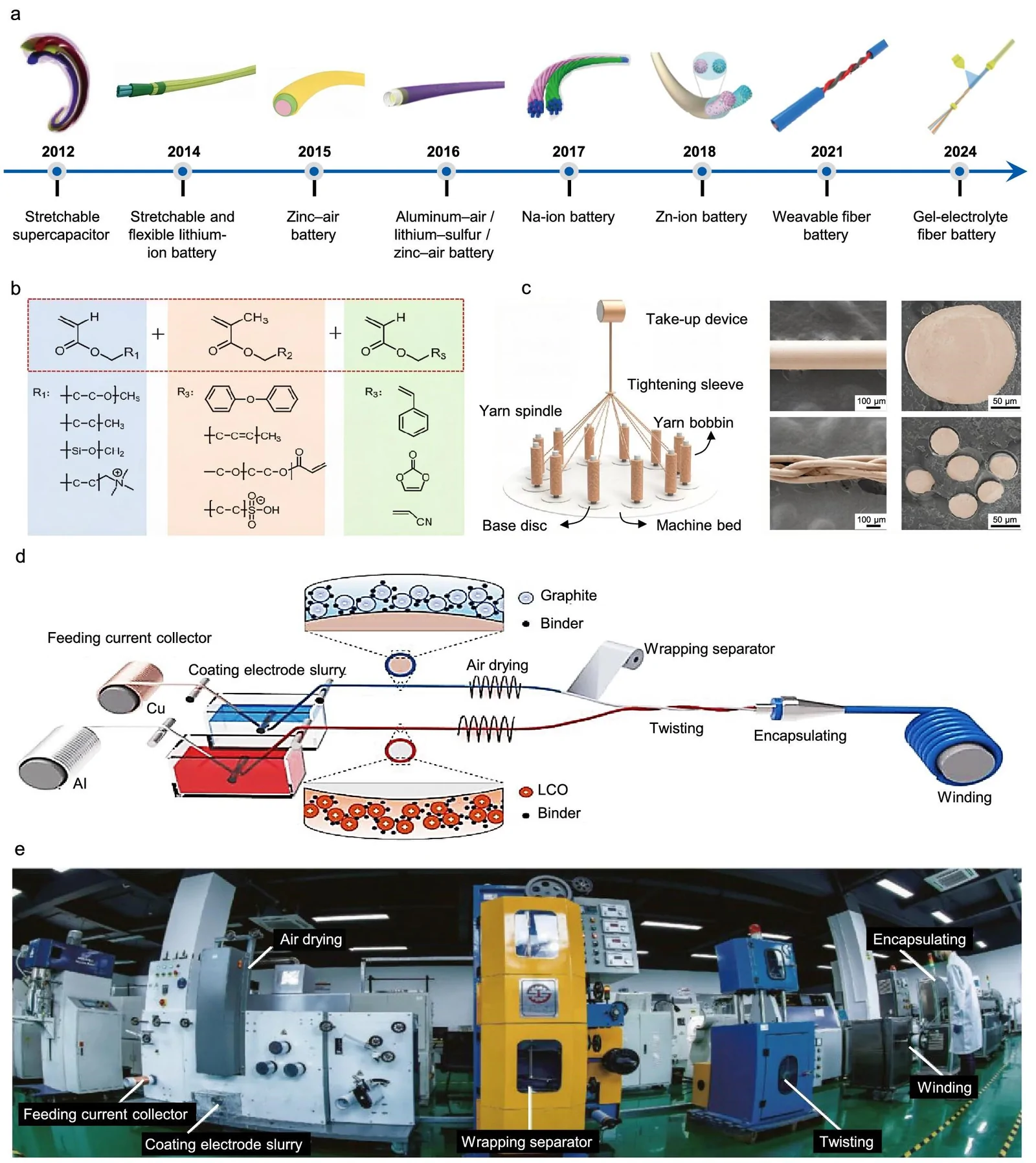
Material innovation and continuous manufacturing of energy-storage fiber devices. (a) A summary of the development of fiber-shaped lithium-ion and aqueous batteries, highlighting the increase in energy density and mechanical durability. (b) Detailed view of advanced gel electrolytes and active materials that provide high ionic conductivity while maintaining a solid-state-like safety profile, essential for wearable and implantable applications (adapted from [212]). (c) Strategies for engineering high-conductivity, flexible current collectors that minimize internal resistance and enhance rate performance (adapted from [7]). (d and e) Illustration of the continuous fabrication process and the transition to an industrial-scale production line. This marks the leap from batch-wise manual assembly to kilometer-length, high-yield manufacturing, which is a prerequisite for the commercialization of electronic textiles (adapted from [6]).

As the primary power modules for autonomous wearable systems, energy-storage fiber devices, commonly referred to as fiber batteries or supercapacitors, have transitioned from nascent proof-of-concept demonstrations to a sophisticated stage of materials and system engineering. The fundamental value proposition of the fiber form factor lies in its ability to decouple the total energy capacity from the device thickness. Unlike planar batteries, where increasing capacity often necessitates thicker, more rigid electrodes that compromise user comfort, integrated-fiber electronic systems achieve energy scaling through length extension or textile-area expansion without sacrificing breathability or multi-axial flexibility. Since the early demonstrations of carbon nanotube-based filaments in 2013 [200], the field has rapidly expanded to encompass diverse chemistries, including lithium-ion, lithium–sulfur, and zinc–air systems (Fig. 10a) [201–205]. However, an evaluative look at this progress reveals that the transition is not merely about miniaturizing bulk batteries; it is a fundamental reimagining of electrochemical kinetics and mechanical stability within the confines of a 1D geometry.

The architectural evolution of fiber batteries has been defined by the need to resolve the mechanical–electrochemical mismatch inherent in 1D systems. Early innovations focused on structural stress-buffer mechanisms, such as the helical dual-electrode winding introduced in 2014, which enabled the device to withstand thousands of bending cycles by converting axial strain into torsional displacement [206]. While these structural designs improved durability, a deeper physical challenge remained: the non-linear relationship between fiber length and internal resistance [207–209]. In planar electronics, scaling is relatively straightforward, but in fiber devices, a high aspect ratio can lead to a precipitous decrease in power density as the length increases. This was theoretically codified in 2021 through the derivation of scaling laws for fiber batteries, providing the first rigorous physical guide for optimizing internal resistance in kilometer-scale systems [6]. Later, a solution-extrusion production line was proposed, and kilometer-scale fiber batteries with high structural consistency and compatibility with commercial textile equipment were demonstrated, an important step toward large-area smart fabrics [210]. In 2022, a thermal-drawing route was introduced for Li-ion battery fibers, opening a promising fabrication axis but facing material selection and high-temperature processing limitations that constrain the achievable energy density and flexibility [211]. Furthermore, recent shifts toward *in situ* polymerization of gel electrolytes have created interlocked electrode–electrolyte interfaces, achieving energy densities of approximately 128 Wh·kg^−1^ while suppressing the interfacial delamination that resulted in earlier batch-processed prototypes (Fig. 10b and c) [212].

The realization of continuous manufacturing represents the most significant technical hurdle in transitioning fiber batteries from laboratory samples to industrial commodities. Conventional planar electrode fabrication techniques, such as slot-die coating and rolling, are fundamentally incompatible with high-curvature fiber surfaces, leading to nonuniform stress distributions and active material detachment, resulting in inconsistent electrochemical performance. Currently, two primary manufacturing paradigms have emerged: thermal drawing and solution extrusion. Thermal drawing offers the advantage of multi-material integration within a single high-precision step, yet the narrow thermal-processing windows of high-capacity active materials often limit it. In contrast, solution-extrusion production lines have demonstrated greater compatibility with established textile equipment, enabling the production of kilometer-scale battery fibers with high structural uniformity. However, the field currently lacks an end-to-end, closed-loop manufacturing ecosystem. To bridge this gap, future production lines must integrate inline diagnostics, such as real-time Raman mapping of the coating morphology or inline electrochemical impedance spectroscopy (EIS), to provide automated feedback and ensure per-meter consistency, which is essential for the large-area integration required in smart fabrics (Fig. 10d and e).

Beyond manufacturing, current research is undergoing a paradigm shift from single-component optimization toward system-level synergy [5,213–225]. A central conflict in the current state of the art is the trade-off between active material loading and kinetic performance. To achieve commercially viable energy densities, fibers must carry a high mass of active materials; however, increasing the diameter of the active layer typically increases the path length for ion and electron transport, leading to sluggish rate capabilities and particle delamination. To mitigate this, researchers are now engineering three-dimensional, continuous conductive scaffolds and hierarchical pore architectures (Fig. 10c) [7]. These bionic designs maintain accessible ion pathways even under high mass loading, effectively decoupling the kinetic limitations from the structural requirements. Complementary advances in interface chemistry, such as the use of elastic, solid-state electrolytes with nanoscale interface modifiers, have proven essential for lowering interfacial impedance and suppressing side reactions during cyclic strain during daily wear [201,226,227].

To achieve true industrialization, the strategic roadmap for energy-storage fibers must move toward a four-axis synergistic framework. First, electrode scaffolds must be redesigned to support high active material loading without sacrificing mechanical compliance. This involves a move toward modular designs (e.g. core–shell or graded architectures) that can be tuned for specific electrochemical loads. Second, electrolyte technologies must evolve into deformation-resilient solids that maintain high ionic conductivity while serving as mechanical adhesives to stabilize the interface under repeated strain. Third, as discussed, continuous manufacturing platforms must be standardized, shifting the focus from laboratory-scale batch stability to industrial-scale throughput with real-time quality assurance. Finally, the community must establish harmonized testing protocols tailored to 1D geometries, including standardized metrics for deformation-dependent impedance, rate performance under stretch, and fatigue retention. When these four dimensions, namely the material chemistry, structural mechanics, manufacturing scale, and rigorous standardization, advance in concert, energy-storage fibers will finally evolve into the reliable, textile-compatible power backbones required for the next generation of pervasive ambient intelligence and autonomous wearable systems.

### Luminescent fiber devices

Luminescent fibers represent the visual interface of the fiber electronics ecosystem, embedding light-emitting functionality into one-dimensional filaments that range from microscopic diameters to millimeter-scale cords. Their primary value proposition lies in decoupling high-performance optoelectronics from the rigid, planar form factors that have historically dominated the display industry. By preserving the inherent breathability, drape, and tactile comfort of textiles, these fibers transform traditional garments into dynamic, distributed display systems for human–machine interactions, healthcare monitoring, and aesthetic expression [228–230]. However, the transition from a simple glowing thread to a high-resolution display fabric involves navigating a complex landscape of material chemistry, optical physics on curved surfaces, and the topological challenges of textile weaving.

The evolutionary trajectory of luminescent fibers has been defined by a move away from hybrid systems toward intrinsically flexible architectures. Early strategies relied on a locally rigid, globally flexible paradigm, in which miniaturized inorganic light-emitting diodes (LEDs) were soldered or thermally drawn into composite fibers [231–233]. While inorganic LEDs provide unrivaled luminance and longevity, they create mechanical discontinuities that compromise the hand feel of the textile and present significant assembly hurdles. This motivated the shift toward organic light-emitting diodes (OLEDs), which promise the brightness and full-color gamut of commercial screens in a flexible format. However, OLEDs face a fundamental geometric bottleneck: vacuum evaporation techniques struggle to maintain thin-film uniformity across the high-curvature, 1D surfaces of fibers, often resulting in localized hot spots or premature electrical failure [174,234–236]. Consequently, powder-based alternating current electroluminescence (ACEL) has emerged as the most industrially viable route. Despite its higher driving voltages, the ACEL is uniquely suited for textiles because it is solution-processable, insensitive to minor film roughness, and chemically robust enough to withstand the rigors of machine washing and mechanical friction.

**Figure 11.**
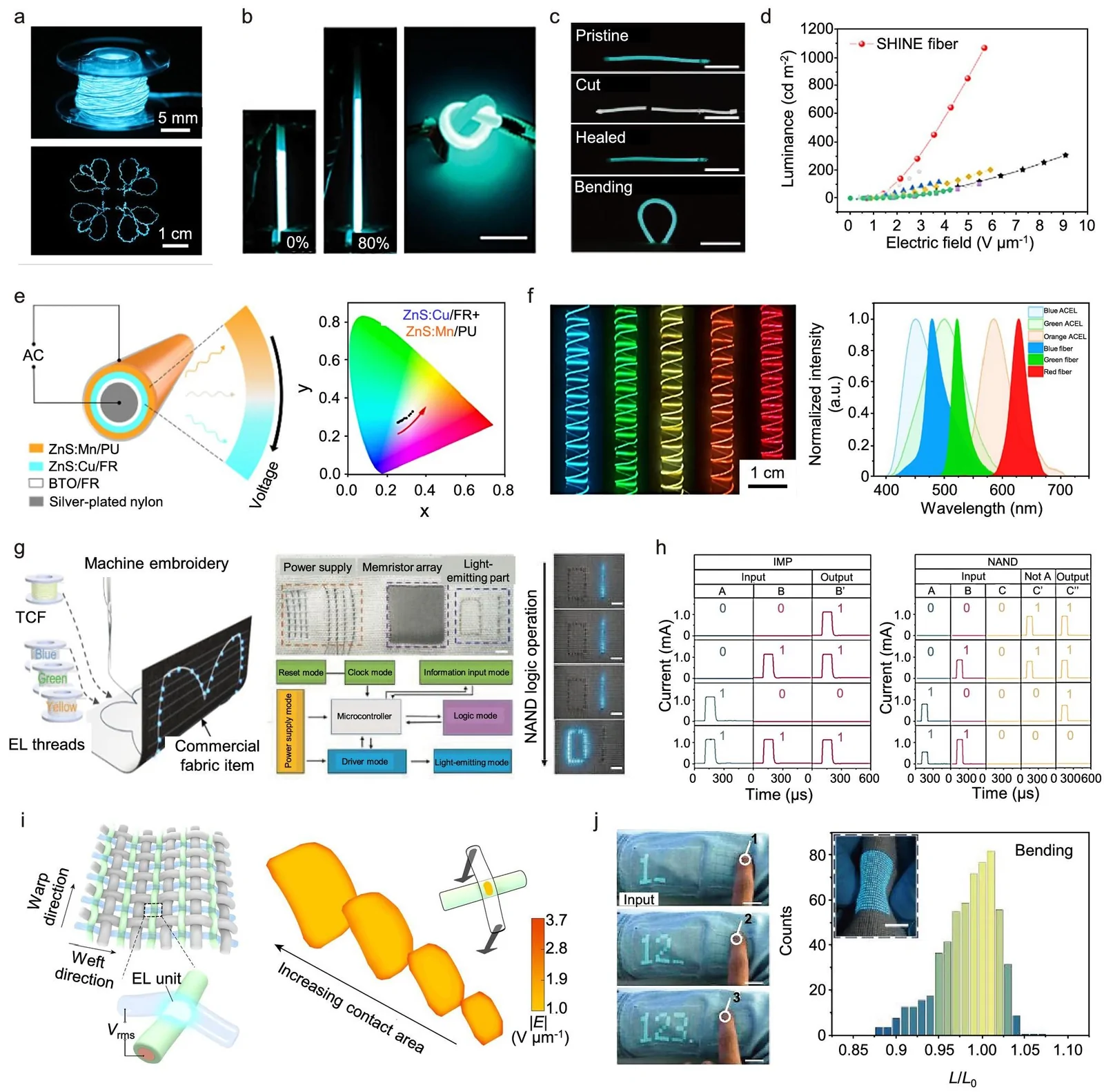
From luminescent fibers to high-resolution display textile systems. (a) Structural overview of alternating current electroluminescent (ACEL) fibers with sheath-core architectures (adapted from [237]). (b) Stretchable ACEL fibers (adapted from [238]). (c and d) High-brightness self-healable fibers and the brightness comparison chart, ensuring device longevity under real-world mechanical stresses (adapted from [239]). (e) Strategies for dual-emission-layer color-changing fibers (adapted from [240]). (f) Strategies for quantum dot-luminescent fibers, achieving high-brightness and broad-gamut emissions across the visible spectrum (adapted from [241]). (g) The evolution from fixed-pattern luminescent textiles to active-matrix display systems (adapted from [242,243]). (h) Luminescent fiber-based display system and the basic logic-in-memory function of IMP and NAND (adapted from [244]). (i and j) By utilizing warp-weft lapped architectures, fibers are transformed into interactive display pixels, enabling the fabric to display information within a single textile intelligence platform (adapted from [1]).

The architectural design of these fibers is as critical as the materials themselves. The field has largely moved from wound designs to coaxial geometries. Coaxial ACEL fibers, popularized since 2017, utilize a transparent conductive sheath that enables 360°, omnidirectional light extraction [238,245,246]. This radial symmetry is not merely an aesthetic choice. It optimizes the optical field distribution and ensures the light intensity remains consistent regardless of the fiber’s orientation within a weave. Advances in dielectric materials and refinements in fabrication processes have facilitated a continuous increase in their luminance (Fig. 11a–d). Furthermore, the incorporation of color-conversion materials, such as quantum dots, has expanded the color gamut of these fiber-based light-emitting systems (Fig. 11e and f). Recent innovations have pushed this further by integrating multiple functionalities, such as self-healing fibers with a magnetic core and a hygroscopic hydrogel sheath (Fig. 11c) [239]. By enabling the restoration of 98.6% of luminance after total mechanical severing, these designs address the high fragility of 1D electronics, moving the field toward damage-tolerant wearable systems. To enable color tuning, multiple ACEL phosphors have been blended into active layers. Their differing dielectric responses produce voltage-dependent color switching between the inner and outer electrodes [240]. Because voltage control cannot independently tune luminance and color, some teams have added electrochromic shells (electrochemically deposited) to create dynamically tunable fibers. However, the achievable color gamut, particularly deep red and absolute luminance, remains constrained by the intrinsic limits of current electroluminescent materials [247].

The true paradigm shift, however, occurred when luminescent fibers transitioned from being standalone light ropes to addressable pixels within a fabric (Fig. 11g and h). The 2021 demonstration of a large-scale display textile (6 m × 25 cm) represents a milestone in system-level integration [235]. By exploiting the warp–weft topology of traditional weaving, where each crossing point between a conductive weft and an active-layer-coated warp functions as an independent pixel, researchers have achieved true pixelation with more than 500 000 units (Fig. 11i and j) [1]. This approach elegantly bypasses the need for individual-pixel patterning on single fibers and instead uses the weave’s structure to define the circuit logic. While this was a monumental proof-of-concept, the system still operates within a luminance-color trilemma: current ZnS-based ACEL phosphors lack efficient deep-red emitters, limiting the display to single-color or narrow-gamut visuals with a peak luminance of approximately 115 cd·m^−2^, sufficient for indoor use but far below the requirements for outdoor visibility.

Despite rapid progress, several tightly coupled challenges must be solved before luminescent fibers become widely practical. First, the use of emitter materials remains a bottleneck. Organic emitters can achieve OLED-level luminance and a full-color gamut, but are chemically fragile and require near-perfect thin films on curved fibers or porous textile substrates. In contrast, ACEL phosphors are mechanically robust and washable but require high drive voltages and offer limited brightness and color range (notably weak deep-red emission). Bridging this gap requires new emitter chemistries that combine ACEL durability with OLED-like luminance and color breadth for low-voltage, high-efficiency electroluminescent materials. Second, color generation and optics on cylindrical surfaces are not straightforward extensions of planar RGB rules. Optical field distribution, viewing geometry, and spatial mixing differ on cylindrical surfaces. Therefore, systematic optical characterization and design rules for trichromatic fiber ensembles (geometry, spacing, and viewing-angle effects) are needed to establish practical color-mixing rules. Third, resolution and pixel scaling are major technical hurdles. Current display textiles have pixel sizes >200 µm and pitches >800 µm, which are far coarser than those of commercial planar panels. Delivering fine, uniform pixels will require reliable deposition and patterning of functional layers on sub-100-µm-diameter fibers. A detailed understanding of local electric-field distributions and carrier transport at fiber crossings is needed to enable uniform per-pixel drive. Fourth, system integration and driving architectures must be rethought for display textiles. Robust, washable interconnects, electrical matching across heterogeneous fiber devices, distributed power and per-pixel driving, and the elimination of rigid peripheral electronics are all necessary for moving from attach electronics to fabric to truly weave electronics into fabric for comfort and long-term wearability.

Meeting these challenges demands concurrent advances in materials, optics, microfabrication, and textile engineering. Priority directions include the development of durable, low-voltage, full-spectrum emitters; optical design methodologies for curved emitters; color-mixing rules for fiber arrays; microscale deposition and patterning techniques compatible with high-throughput textile processes; and textile-native integration strategies that embed flexible drivers, distributed power management, and washable interconnects into the weave. Standardized durability tests (mechanical cycling, washing, and environmental aging) and reliability metrics will also be essential to accelerate adoption. If these elements are advanced in parallel, luminescent fibers can evolve from decorative light ropes to high-quality, full-color, high-resolution, washable display textiles, unlocking new markets in assistive devices, wearable communication, and ubiquitous, fabric-integrated displays.

### Sensing fiber devices

**Figure 12.**
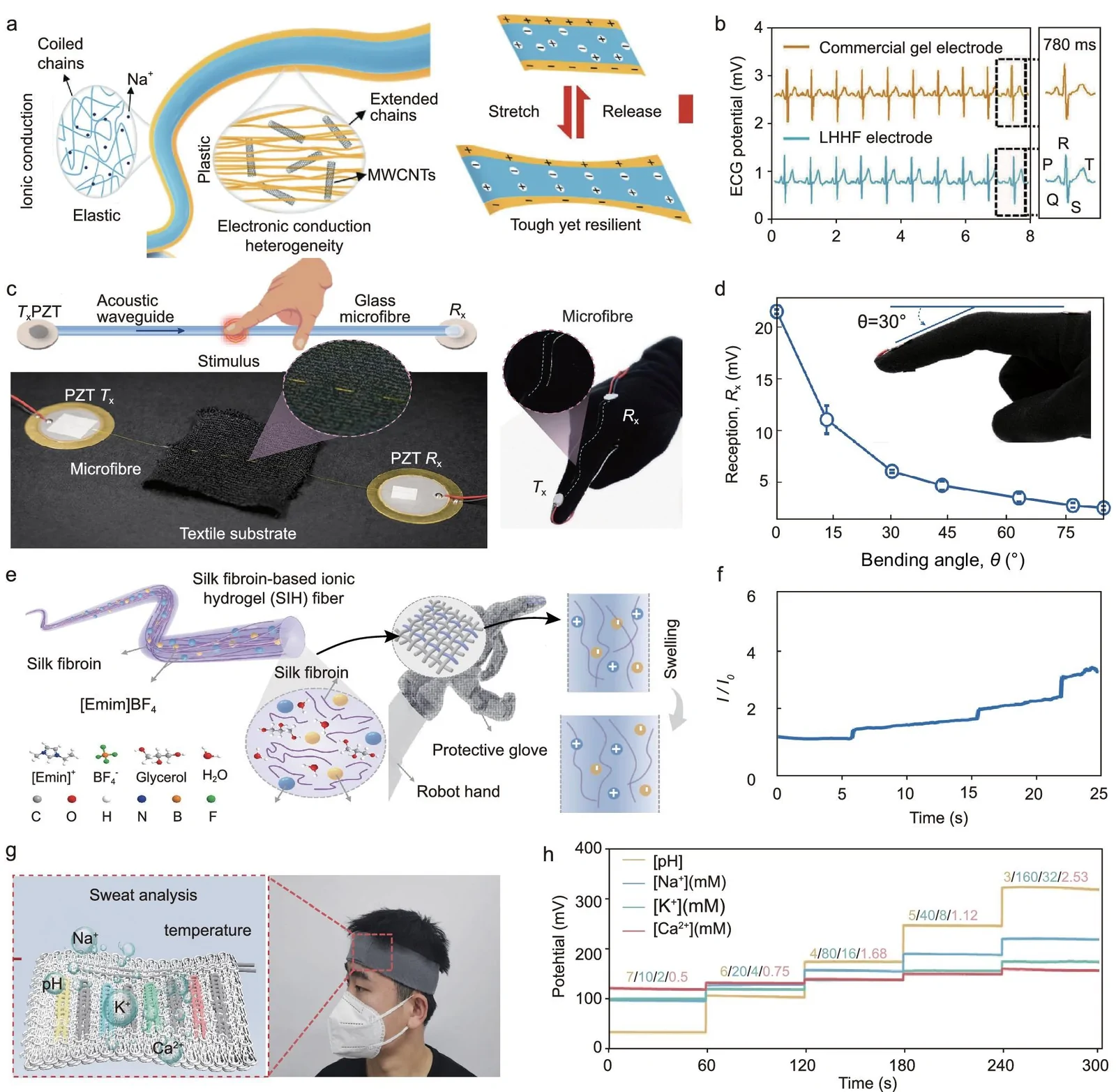
Wearable sensing fibers for multimodal physiological monitoring. (a and b) Soft, low-impedance fibers designed for the continuous acquisition of ECG signals, providing clinical-grade monitoring without the discomfort of traditional rigid electrodes (adapted from [254]). (c and d) Piezoresistive or capacitive fibers optimized for bending and strain detection, enabling the precise tracking of human joint movements and respiratory patterns (adapted from [264]). (e and f) Porous humidity-sensing fibers that offer real-time detection of skin moisture and ambient humidity, critical for thermoregulation and comfort assessment (adapted from [260]). (g and h) Electrochemical fibers functionalized with ion-selective membranes or enzymes for the selective detection of target ions (e.g. K⁺, Na⁺) or molecules (e.g. glucose) in sweat, facilitating non-invasive, continuous health diagnostics (adapted from [269]).

Sensing fiber devices, or fiber sensors, are soft, textile-compatible sensing elements engineered for continuous on-body acquisition of physiological and environmental signals. Their conformability, breathability, low areal mass, and direct weaveability make them well-suited for long-term health monitoring, human–machine interfaces, medical wearables, and environmental sensing platforms [248,249]. Early implementations involved coating conventional textile yarns with conductive materials and transducing them with mechanical stimuli (strain, pressure, etc.) *via* resistance or capacitance changes [250]. Subsequent work introduced new fiber architectures, namely, the coaxial, hollow, multi-core, and related geometries, that substantially improve signal stability under stretch and bending while reducing drift and hysteresis [251,252]. More recent advances in transduction mechanisms, active materials, and device layouts have broadened the sensing repertoire from purely mechanical measurements to electromechanical, thermal, humidity, and chemical analytes, greatly enhancing the multimodal capabilities of fiber sensors [253]. Broadly, wearable fiber sensors can be classified into two families: physical sensors (Fig. 12a–f), which measure mechanical (strain, pressure, and bending), electrophysiological (ECG: Electrocardiogram, EEG: Electroencephalogram, EMG: Electromyography) and environmental/physiological (temperature, humidity, and evaporation) parameters, and chemical sensors, which detect ions, metabolites or volatile compounds in sweat, saliva or air (Fig. 12g and h).

Physical sensing fiber devices deliver high-quality electrophysiological, mechanical, and environmental measurements by combining conductive fiber electrodes, engineered interfaces, and tailored structural designs. Electrophysiological fiber devices achieve reliable ECG, EMG, and EEG acquisition by creating low-impedance, conformal skin–electrode contacts (Fig. 12a and b) [254]. Conductive coatings are typically deposited onto flexible cores or woven from conductive polymers, carbon nanomaterials, or metal nanostructures to form continuous, wearable electrodes [255,256]. Minimizing interface impedance and motion artifacts is typically accomplished through modulus matching to the skin, micro-structured electrode surfaces, and fabric-level conformability engineering [257]. Mechanical fiber sensors transduce strain, pressure, and bending into electrical signals *via* piezoresistive, piezo-capacitive, piezoelectric, or triboelectric mechanisms [258–260]. Coaxial and multi-core geometries that distribute stress more uniformly, combined with engineered microstructures in the conductive layer (microcracks, wrinkles, pores), deliver high sensitivity while preserving linearity and dynamic range [261–263]. Emerging modalities extend this platform. For instance, acoustic-waveguide fibers convert surface vibrations into electrical signals for voice interaction and novel health monitoring modalities (Fig. 12c and d) [4,264]. While temperature and humidity fiber devices exploit thermosensitive and hygroscopic materials (conductive polymers, hydrogels, and carbon fillers) whose resistance or capacitance track environmental changes, these devices enable epidermal thermal-moisture monitoring (Fig. 12e and f) [260,265]. These functions are typically realized by coating or compositing conductive and responsive materials onto fiber backbones. However, many designs exhibit response lag, cross-sensitivity to confounding environmental factors, and residual motion artifacts that complicate signal interpretation [266–268].

Chemical-sensing fiber devices enable continuous, real-time monitoring of biofluids (sweat, saliva, and tears) and ambient species, targeting ions, small-molecule metabolites, and volatile organic compounds to reveal physiological state or environmental exposure. Transduction modalities include electrochemical methods (potentiometric, amperometric, and impedance) and optical and colorimetric readouts. High selectivity is typically achieved by functionalizing electrode surfaces with ion-selective membranes, enzyme layers, or molecularly imprinted polymers for analytes such as glucose, lactate, pH, and common electrolytes in sweat (Fig. 12g and h) [269]. Gas detection typically relies on chemiresistive films (metal oxides and conductive polymers) whose adsorption or reaction with target gases (NH₃, CO₂, and volatile organic compounds) results in measurable changes in resistance. Structurally, these active chemistries are deposited onto conductive fiber backbones by coating, electroplating, or *in situ* polymerization to form flexible, conformal sensing interfaces [267,270]. In realistic use settings, chemical sensing must overcome additional hurdles. For instance, complex biofluid matrices, cross-sensitivity among analytes, variable sweat rates, and surface fouling can decrease selectivity and long-term stability [271]. Recent design trends mitigate these limitations by embedding reference electrodes and multi-channel architectures, integrating microfluidic sweat-handling and preconcentration units, and implementing on-fiber signal-processing and self-calibration routines to enable robust multimodal measurement. These strategies, combined with antifouling coatings and robust immobilization chemistries, are shifting chemical fiber sensors from single-analyte demonstrations toward reliable, multimodal *in situ* monitoring platforms suitable for biomarker screening, environmental safety, and metabolic assessment.

From a technology-readiness perspective, physical-sensing fiber devices have achieved sufficient manufacturability and system integration for field demonstrations in sports monitoring and rehabilitation. In contrast, chemical sensing fiber devices lag and remain largely at the prototype-to-pilot stage, significantly constrained by reagent stability, reproducibility, and environmental robustness. Across both domains, translation is blocked by several tightly coupled, cross-cutting challenges that must be addressed in parallel. First, achieving the correct trade-off among sensing performance, wearer comfort, and textile integrability requires active materials and device layouts that survive washing, repeated mechanical fatigue, and direct skin contact while preserving low mass, breathability, and conformability. Second, long-term operational robustness under real-world wear demands improvements in interface chemistry, encapsulation, and mechanical design, together with adaptive signal-processing algorithms that suppress motion and environment-induced artifacts. Third, the absence of standardized evaluation protocols and metrics impedes comparison and commercialization. The community needs harmonized test frameworks covering material durability, mechanical cycling, electrical performance, data quality, and biomedical relevance. Fourth, system-level integration remains immature. Reliable, washable interconnects, on-fiber signal conditioning, low-power edge processing, compact energy solutions, and seamless textile embedding of drivers and connectors are all required to move from laboratory demos to commercial products. Beyond individual sensing functionalities, the field is rapidly advancing toward integrated platforms capable of multimodal monitoring and autonomous data transmission. The synergy between functional nanomaterials and wireless, battery-free architectures is transforming sensing fibers into comprehensive wearable and even implantable systems for long-term, real-time chemical and biological health diagnostics [272,273].

To accelerate the deployment of sensing fiber devices, research should prioritize four convergent thrusts: (i) design intrinsically robust sensing chemistries and fouling-resistant interfaces that retain activity after laundering and long-term wear; (ii) develop scalable fiber production and textile-integration workflows that lock in sensor functionality through repeated deformation and washing; (iii) create adaptive electronics and algorithms for on-fiber preprocessing, motion-artifact rejection, and self-calibration; and (iv) establish standardized, application-oriented performance metrics and accelerated-aging protocols to qualify systems for clinical, consumer, and industrial use. Progress along these axes will be necessary to realize reliable, long-lived, textile-native sensing systems for healthcare, environmental monitoring, and interactive textiles.

### Implantable fiber devices

**Figure 13.**
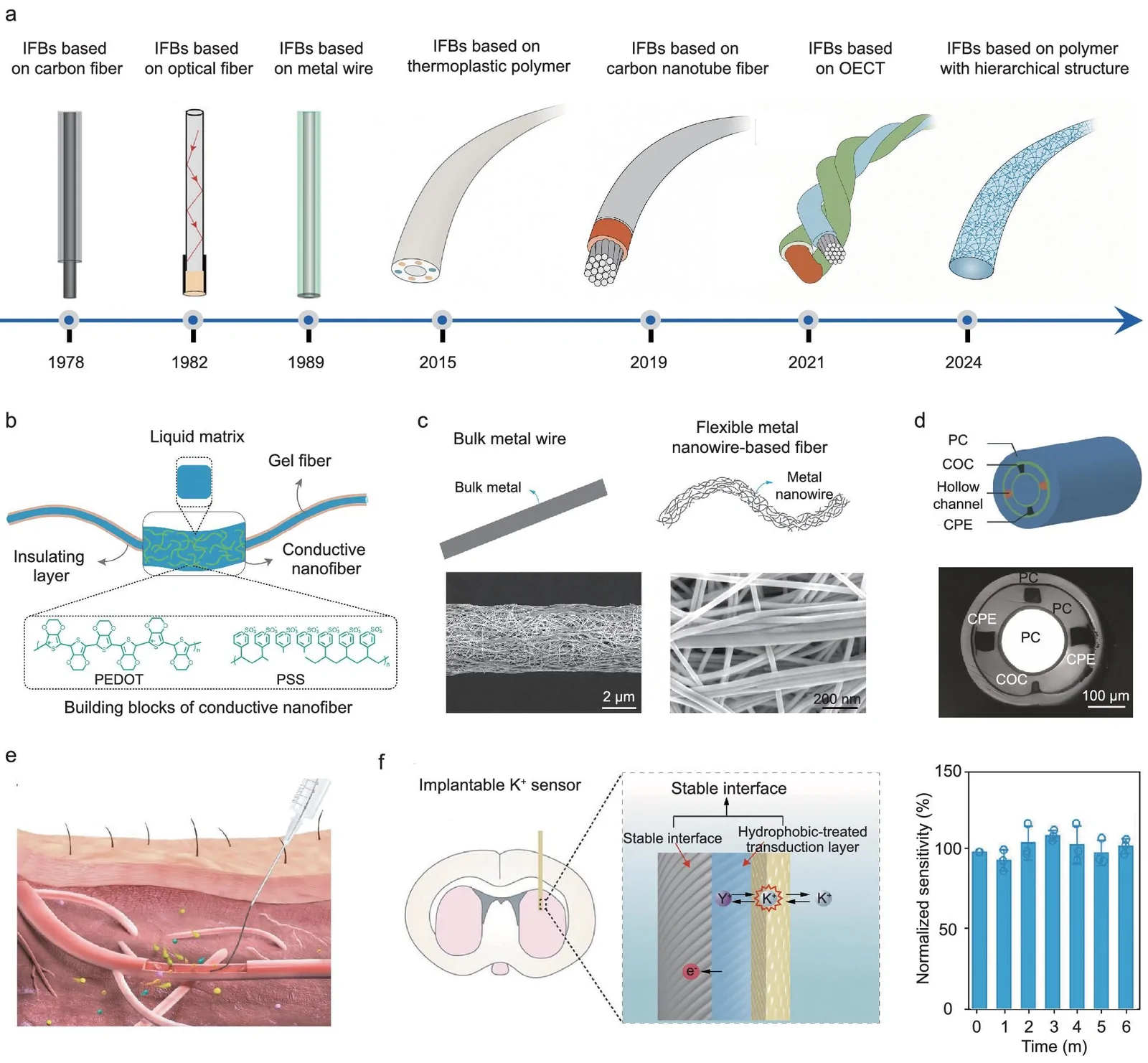
Implantable fiber sensors. (a) Evolution of implantable fiber biosensors, highlighting the shift toward tissue-like, flexible probes for chronic neural and biochemical interfacing (adapted from [275]). (b) Conductive-polymer-nanowire-assembled implantable fiber neural electrode (adapted from [285]). (c) All-metal flexible fibers obtained by assembling metal nanowires to achieve stable, low-impedance interfaces that minimize the inflammatory response of biological tissue (adapted from [293]). (d) Multimodal-fiber neural probe fabricated by thermal drawing, combining optical, electrical, and chemical channels within a single filament for comprehensive mapping of brain activity (adapted from [284]). (e) Hierarchically helical CNT-fiber electrochemical sensor for long-term *in vivo* sensing of disease biomarkers (adapted from [9]). (f) Fiber sensors for long-term *in vivo* monitoring of K^+^ concentrations in the brain (adapted from [277,281]).

Real-time, *in vivo* monitoring of physiological signals is central to understanding biology, enabling early diagnosis and supporting personalized therapies. Implantable fiber sensors, which exploit slender, one-dimensional geometries, offer minimally invasive access, high spatial precision, and the ability for continuous, on-site electrical and biochemical monitoring and modulation, and have therefore become key approaches for in situ bioelectrical and biochemical sensing [9,274–278]. The earliest intracerebral recordings (1950s) used metal microwires, but rigid electrodes can cause chronic tissue damage due to a severe mechanical mismatch with soft tissue [279]. This limitation motivated the development of soft, tissue-compliant substrates (carbon allotropes [280–283], conductive polymers [102,284–286], and hydrogel composites) and the rapid evolution of soft implantable fiber devices (Fig. 13a). Carbon fibers were first applied to monitor dopamine release in the rat striatum in 1978 [275]. In 2015, thermoplastic polymers were drawn by thermal drawing into multifunctional fiber neural probes [284]. In 2020, flexible electrochemical fiber sensors based on hierarchically helically assembled CNT fibers enabled long-term *in vivo* monitoring of multiple disease biomarkers [9]. CNT fibers, which combine high flexibility with strong electrochemical performance, have since become a preferred substrate for various implantable fiber sensors, including *in vivo* neural recording and modulation [286].

A broad range of flexible conductive fibers has been developed from carbon materials [287–289], conductive polymers [284–286], and hydrogel composites [290], with impedance further reduced by surface coatings and micro/nanotexturing [287,291]. Of particular interest are fiber electrodes assembled directly from conductive nanomaterials. Unlike homogeneous conductive fibers, these assemblies can simultaneously achieve ultralow modulus and low impedance without complex post-treatments. Examples include CNT-based [288], graphene-based [289], conductive-polymer-nanowire [285] and metal-nanowire [292] assembled fiber electrodes. For instance, a fiber neural electrode was constructed by hierarchical assembly of conductive-polymer nanowires [285], achieving a kilopascal-level modulus, high conductivity, and low impedance. These electrodes recorded stable action potentials in large animals during vigorous motion (Fig. 13b). The impedance of graphene-based fiber neural electrodes was reported to be two orders of magnitude lower than that of the same-sized platinum wires, resulting in *in vivo* signal-to-noise ratios of up to 9.2 [289]. All-metal fiber electrodes with both high flexibility and high conductivity were prepared from silver nanowires via a continuous wet-spinning process. These electrodes have been demonstrated to function as neural probes with stable chronic recording of single neurons (Fig. 13c) [293]. Beyond material optimization, fabrication strategies have been used to add multifunctionality. A thermal drawing technique was employed to fabricate multimodal fiber probes that integrate electrical recording, optical stimulation, and fluid delivery for combined neuromodulation and neurochemical experiments (Fig. 13d) [284]. Thermal-drawn fibers were coated with hydrogels to endow them with adaptive mechanical properties and to improve chronic stability [290]. Most recently, a high-density micro/nano-patterned film can be rolled into one-dimensional fiber devices to produce high-density, multimodal fiber neural electrodes, providing a scalable strategy for dense functional integration [294].

Implantable neural probes transduce local extracellular ionic currents produced by action potentials into measurable electrical signals *via* capacitive/electrode–tissue coupling and are the principal tools for decoding brain activity [287,295]. Fiber-shaped neural electrodes attract attention because their one-dimensional geometry and mechanical compliance reduce insertion damage and chronic foreign-body responses. The critical electrode metrics are a low Young’s modulus (for mechanical compliance) and low electrochemical impedance (for efficient electrode–tissue charge transfer and high-quality recordings) [296,297]. Implantable fiber chemical sensors perform *in situ*, continuous, spatially resolved monitoring of chemical species in subcutaneous tissue, blood vessels, and deep organs. Typical devices are two- or three-electrode electrochemical constructs: a conductive fiber working electrode functionalized with selectively active materials, a stable reference electrode, and a high-surface-area counter electrode. Chemical concentration is transduced electrochemically into continuous current or potential signals, enabling rapid, high-resolution readout. Advances in flexible electrodes, sensing chemistries, and detection principles have enabled *in vivo* monitoring of dopamine, glutamate, ascorbate, glucose, hydrogen peroxide, ions, and other analytes [9,281–283]. The first use of a fiber organic electrochemical transistor (fiber-OECT) for highly sensitive *in vivo* biochemical sensing was reported in 2021 [283]. Subsequent work has produced implanted fiber-OECTs with high transconductance, fast response, and high flexibility [102,298]. Collectively, these advances in materials, device architectures, and fabrication are driving implantable fiber sensors toward integrated, intelligent platforms for personalized health management and precision medicine.

Because the in-body environment is complex, implantable chemical sensors must meet stringent specifications, including high sensitivity and low detection limits, high selectivity and reversibility, and long-term *in vivo* stability [275]. For example, an implantable-fiber OECT was developed that amplifies detection currents for H₂O₂, glutamate, dopamine, and glucose, enabling high-sensitivity monitoring of low-concentration dopamine in the brain for up to 7 days [283]. A molecularly imprinted polymer-based fiber sensor was demonstrated for the selective in vivo monitoring of vanillylmandelic acid with excellent selectivity [299]. To achieve long-term stable in-body sensing, researchers focus on stabilizing the device–tissue interface and internal electrode interfaces. A biomimetic, hierarchically helical CNT-fiber electrochemical sensor whose bending stiffness matched that of the surrounding tissue was developed, establishing a stable tissue–device interface and enabling long-term sensing of disease biomarkers (Fig. 13e) [9]. Fiber sensors that seamlessly adhere to tissue were fabricated using polymeric hydrogel interfaces and stably monitored multiple biochemical species in amniotic fluid [300]. The electrode surface roughness and hydrophobicity were optimized to stabilize internal sensor interfaces, achieving prolonged *in vivo* monitoring of cerebral potassium ion concentrations (Fig. 13f) [281].

Despite significant progress, several tightly coupled barriers still limit the clinical translation of implantable fiber sensors and must be addressed together [3]. First, in sensing chemistry, the palette of robust, immobilizable recognition elements is narrow, and long-term *in vivo* stability is imperfect. Coordinated discovery efforts, accelerated by AI-guided material design, curated databases, and bottom-up screening, are needed to expand target repertoires and develop reliable immobilization strategies. Second, with respect to mechanical adaptability, implantable fibers must not only be soft but also conformable, stretchable, and able to accommodate tissue motion or actively adapt to position. This requires the co-design of soft substrates, hierarchical geometries (helical, segmented, and coaxial), and dynamic anchoring to preserve contact and signal fidelity under large deformations. Third, system integration with multifunctional architectures is required to combine multimodal sensing, closed-loop actuation (stimulation/drug delivery), on-fiber signal conditioning/transmission, and integrated power, but still remains immature. Promising hardware design rules include helical twisting, segmented functionalization, and multilayer coaxial layouts, but module-level coupling and energy-matching schemes must be solved for practical implants. Finally, translation requirements (e.g. biocompatibility, sterilization, anti-fouling, long-term durability, and regulatory validation) pose multidisciplinary challenges that demand coordinated materials, devices, and preclinical and clinical programs. In short, implantable fiber sensors have moved from basic electrodes to multifunctional, high-density platforms capable of *in situ* electrophysiology and biochemistry. To reach clinical utility, the field must broaden and stabilize sensing chemistries, advance mechanically adaptive architectures and interface strategies, develop multimodal fiber systems, and establish rigorous preclinical testing, sterilization protocols, and regulatory pathways. These integrated advances will be decisive for realizing implantable fiber devices for precision neuroscience, continuous biomonitoring, and responsive therapeutics.

### Data-processing fiber devices

Data-processing fiber devices represent a critical frontier in fiber electronics, addressing the growing demand for on-fiber computational capabilities that support intelligent, real-time decision-making in wearable and embedded systems [301,302]. This domain encompasses two complementary technological pathways: neuromorphic fiber devices, which emulate biological neural architectures to enable energy-efficient in-memory computing, and fiber-integrated chips, in which miniaturized silicon-based systems are seamlessly incorporated into fibrous substrates to achieve high-performance processing. Neuromorphic implementations leverage memristive materials and synaptic transistors woven into textiles to perform parallel, low-power computations directly at the sensing site, dramatically reducing the latency and energy consumption associated with data transmission [303–305]. Concurrently, fiber-integrated system-on-chip (SoC) technologies enable complex signal processing and wireless communication functions within the fiber itself, supported by advanced interconnection strategies that maintain mechanical flexibility [249,306–309].

**Figure 14.**
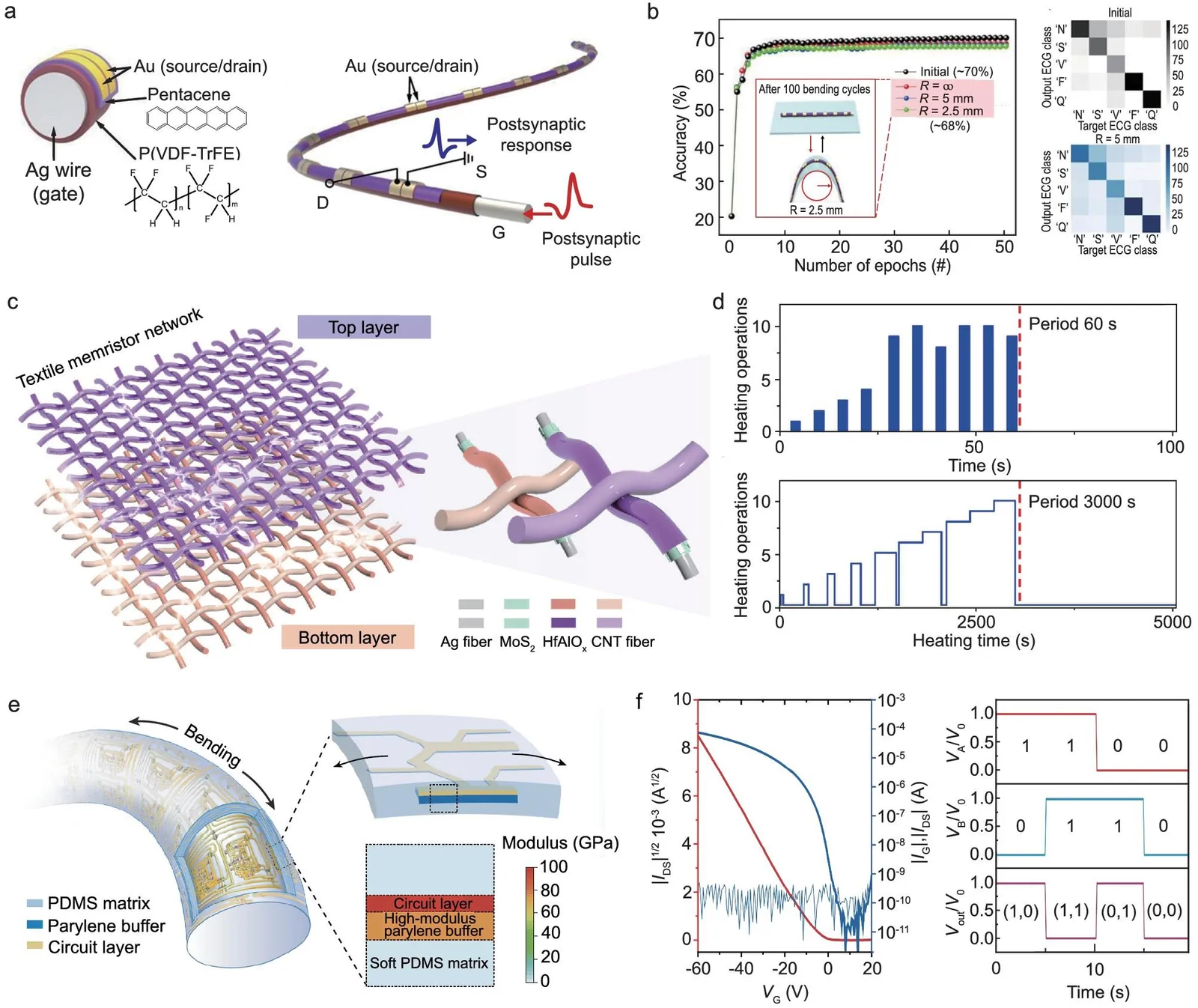
Data-processing fiber devices. (a and b) Organic artificial synapses that mimic biological synaptic plasticity, exhibiting high robustness and accuracy in pattern recognition even under severe mechanical disturbances, and representing a fundamental building block for neuromorphic computing in textiles (adapted from [311]). (c and d) A reversible nonvolatile memory and a volatile threshold-switching memristor on a reconfigurable textile chip (adapted from [312]). (e and f) Illustration of multi-layered spiral architectures that enable the integration of transistors and basic logic gates (e.g. inverters) within a single filament, along with the voltage output of the gate circuit in the fiber, demonstrating the transition from discrete devices to integrated circuits at the fiber level (adapted from [314]).

Recent work has shown that high-performance neuromorphic elements can be implemented directly on fibrous substrates using tailored materials and device architectures to emulate synaptic functions (plasticity, retention, and short- and long-term memory) while retaining mechanical compliance and reproducibility. For instance, a wearable memristor array was constructed on a flexible fiber substrate, leveraging DNA molecules as a functional layer to facilitate the formation of a conductive channel *via* silver ions [244]. This device exhibits exceptional memory characteristics, including a high switching ratio of 106, a low operating voltage of 0.3 V, and a faster-erasing speed of 20 ns. It also demonstrates the capacity to implement Boolean logic functions with real-time output. In parallel, a high-performance textile memristor was developed using an electric-field-assisted assembly method with robust Pt/CsPbBr_3_ fibers [310]. This device features low cycle-to-cycle variation (<8%) and a low average set voltage of ~0.16 V. These flexible, mechanically stable fiber devices were woven into a scalable array with excellent device-to-device reproducibility, enabling seamless integration into smart clothing for the accurate processing of complex physiological signals. One-dimensional fiber-shaped multi-synapses were designed based on ferroelectric organic transistors fabricated on a 100-μm silver wire (Fig. 14a and b) [311]. These channels were successfully employed as multi-synaptic channels in an e-textile neural network for wearable applications. The device reliably emulates diverse synaptic functions, maintaining high performance even after 6000 repeated mechanical bends. By cross-pointing these 1D synapses, NOR-type textile arrays were constructed, ensuring integrated and leakage-free signal propagation at each synaptic node.

Two complementary trends dominate system-level progress. The first is the creation of intrinsically multifunctional devices. Reconfigurable-fabric memristive networks are capable of performing both artificial synaptic and neuronal functions (Fig. 14c and d) [312]. A textile memristor network (Ag/MoS_2_/HfAlO*_x_*/CNT) whose reconfigurable devices exhibit both long-term synaptic plasticity and volatile neuron-like spiking was demonstrated, enabling a single device to act as an integrate-and-fire element and substantially simplifying the overall neural circuitry. The second trend is the integration of modules toward near-sensor and in-sensor computing. Rather than routing raw sensor data through remote processors, neuromorphic fibers perform front-end preprocessing or embed processing within the sensing element itself, reducing data transfer, latency, and energy expenditure [313]. A smart-textile prototype integrates reconfigurable synapses, neuron primitives, and heating resistors so that sensing, memory, and simple computation occur within a compact, fabric-native unit, improving functional density and utilization efficiency for intelligent wearable applications [312].

These bio-inspired units effectively mimic the distributed processing and low-power consumption of biological systems, providing a robust pathway for localized, brain-like signal interpretation at the skin-textile interface. However, the leap from biomimetic sensing-processing units to sophisticated, programmable fiber devices requires more than just individual synaptic fidelity. It demands the realization of high-density, deterministic digital logic within the 1D architecture. A breakthrough in this direction was recently reported by introducing the concept of fiber integrated circuits (FICs) enabled by a multilayered spiral architecture (Fig. 14e and f) [314]. By geometrically transforming the traditional planar layout into a 1D coaxial-spiral assembly, the researchers achieved an unprecedented integration density of approximately 10^5^ transistors per centimeter. This performance metric bridges the gap between discrete fiber components and modern microelectronics. This architecture allows functional fibers (semiconductors, insulators, and conductors) to be synergistically interwoven, facilitating the execution of complex logic functions such as exclusive-OR (XOR) gates and set-reset (SR) latches. More importantly, to reconcile the high integration density with the rigorous mechanical demands of wearable use, a modulus-heterostructure design was implemented. This structural strategy enables the FIC to maintain stable electrical performance even under extreme stress, such as being crushed by a 15.6-ton container truck, thereby solving the long-standing conflict between the rigidity of high-density electronic components and the flexibility required for textile integration. By converging these advanced digital logic capabilities with the aforementioned neuromorphic learning abilities, data-processing fibers are evolving from standalone prototypes into integrated computational hubs.

Application demonstrations of neuromorphic fiber devices are advancing rapidly in edge computing and health monitoring. Triboelectric sensing, memristive recognition, and display textiles were integrated to form an on-fabric sensing-processing-display pipeline that accurately classified physiological events, including normal, interictal, and ictal epileptic EEG states, with >95% accuracy [315]. A triboelectric all-textile sensor array (TATSA) was developed that can be directly integrated into clothing at key physiological monitoring sites, such as the neck, wrist, and chest, corresponding to pulse wave and respiratory detection zones. Using the TATSA, a personalized, intelligent health monitoring system was established for real-time, remote monitoring, enabling continuous acquisition and analysis of physiological signals for the assessment of cardiovascular disease (CAD) and sleep apnea syndrome (SAS) [316]. Moving toward autonomous, biologically inspired systems, a textile-based multiplexing neuromorphic sensorimotor system was designed [317]. This system integrates sensing and actuation using ion-conductive synaptic fibers (ISFs), amplifiers, and soft artificial yarn muscles while incorporating dendritic interconnects and parallel information pathways. The system exhibits short-term plasticity even in aqueous environments and facilitates biological signal transmission over centimeters while consuming ultralow energy at the femtojoule level. This innovative architecture successfully demonstrates the integration of pro-neuronal sensing and coordinated motor output for adaptive motion control, revealing significant potential for applications in healthcare and autonomous feedback loops.

Despite notable unit-level breakthroughs, neuromorphic fiber devices remain at a critical transitional stage because their large-scale deployment is blocked by tightly coupled manufacturing, systems, and algorithmic challenges. Manufacturing and multilayer integration present major hurdles: conventional high-precision planar lithography and alignment do not translate to curved, micron-scale fiber substrates; thus, realizing high-density logic and reliable multilayer stacks on fibers requires disruptive curved-substrate co-fabrication methods and low-temperature, conformal thin-film processes (variants of atomic layer deposition, conformal printing, wrap-and-roll lithography, etc.) that produce uniform, adherent films without degrading compliant fiber cores [318–320]. System reliability and mechanical–electrical co-design are equally urgent. When fibers are networked into fabrics, maintaining signal integrity, balancing distributed power, and preventing mechanical–electrical failure under repeated bending, washing, and wear become pressing problems. Addressing these demands requires standardized, washable mechanical–electrical coupling interfaces, textile–native interconnect protocols, and design rules that jointly optimize electrical performance and mechanical compliance. Finally, algorithmic and processing efficiency must keep pace with hardware. Practical on-fiber intelligence requires lightweight, noise-tolerant, and variability-tolerant learning and control schemes that run on resource-constrained neuromorphic primitives, as well as training methods robust to device mismatch, drift, and nonstationary inputs. Overcoming these barriers requires a coordinated roadmap that advances fabrication, system engineering, and algorithms in parallel. First, scalable curved-substrate fabrication and assembly techniques that enable uniform, high-density device stacks on fibers should be developed. Second, textile-native interconnect and packaging standards that ensure low-loss, washable electrical coupling and predictable mechanical durability, and co-design power distribution solutions matched to neuromorphic duty cycles should be defined. Third, co-develop algorithmic toolchains and training paradigms tailored to the realities of neuromorphic fiber devices. Finally, testbeds and benchmarks that quantify energy per inference, latency, accuracy under deformation, and lifetime under realistic wash/cycle conditions should be established to drive convergence progress. Potentially, neuromorphic fiber devices can realize the long-promised capability to compute where data are generated, delivering low-power, low-latency, robust intelligence for wearable edge AI, real-time biomedical processing, adaptive textiles, and distributed neuromorphic sensing networks.

In brief, the various types of fiber-shaped functional devices, ranging from energy conversion and storage units to multimodal sensors, logic transistors, and optoelectronic displays, serve as indispensable functional modules for integrated-fiber electronic systems. The transition from individual materials to these 1D device architectures represents the critical realization of specific electronic tasks within a textile-compatible form factor, bridging the gap between molecular engineering and systemic utility. Each device category plays a non-substitutable role in the systemic roadmap of fiber electronics. The fiber-native energy harvesters and batteries provide the foundation for energy autonomy, liberating the final integrated systems from the weight and rigidity of traditional external power sources. Simultaneously, high-fidelity sensing fibers act as the primary interface for complex data acquisition, capturing bio-physical or chemical information and environmental signals with unprecedented spatial resolution and conformability. Most crucially, the emergence of fiber-shaped transistors and logic circuits (e.g. fiber chips) enables local signal processing, allowing the systems to transcend simple passive sensing and achieve active computing within the fabric itself. Furthermore, optoelectronic fibers provide the essential human–machine interface, allowing the system to communicate information directly through visual feedback or interactive displays. The irreplaceability of these devices lies in their unique synergy of electronic performance and textile-like mechanics. Unlike planar electronics that are merely attached to fabrics, these 1D devices are designed to be co-woven or knitted, ensuring that the resulting systems (discussed in the following sections) maintain the inherent comfort, breathability, and washability of clothing. This bottom-up functionalization is the core engine driving the 2035 vision of system-in-a-fiber, where discrete device functionalities are orchestrated into synergistic, autonomous, and wearable intelligent platforms capable of real-world deployment.

## FUNCTION INTEGRATION TO ELECTRONIC FIBER SYSTEMS

The evolution of fiber devices is now reaching a critical juncture, mirroring the trajectory of mature information technologies where progress hinges on system-level integration rather than isolated component optimization. This chapter addresses the pivotal transition from individual, high-performing fiber devices to intelligent, reliable, and fully integrated electronic fiber systems. Real-world applications, from health-monitoring garments to interactive textiles, demand the seamless co-integration of sensing, energy, communication, storage, and computation within a single, textile-compatible form factor. While this integration poses significant technical challenges, a spectrum of innovative strategies has emerged [315], which can be broadly categorized into two complementary paradigms: the internal integration of multiple functional elements and interconnects within a single fiber, and the external assembly of numerous specialized fibers into organized, system-level fabrics. This chapter examines these multi-scale integration pathways, exploring how they collectively enable the development of complex, multifunctional systems that are greater than the sum of their parts, thereby fulfilling the promise of truly pervasive and functional fiber electronics.

### Multi-element integration and intra-fiber interconnects

**Figure 15.**
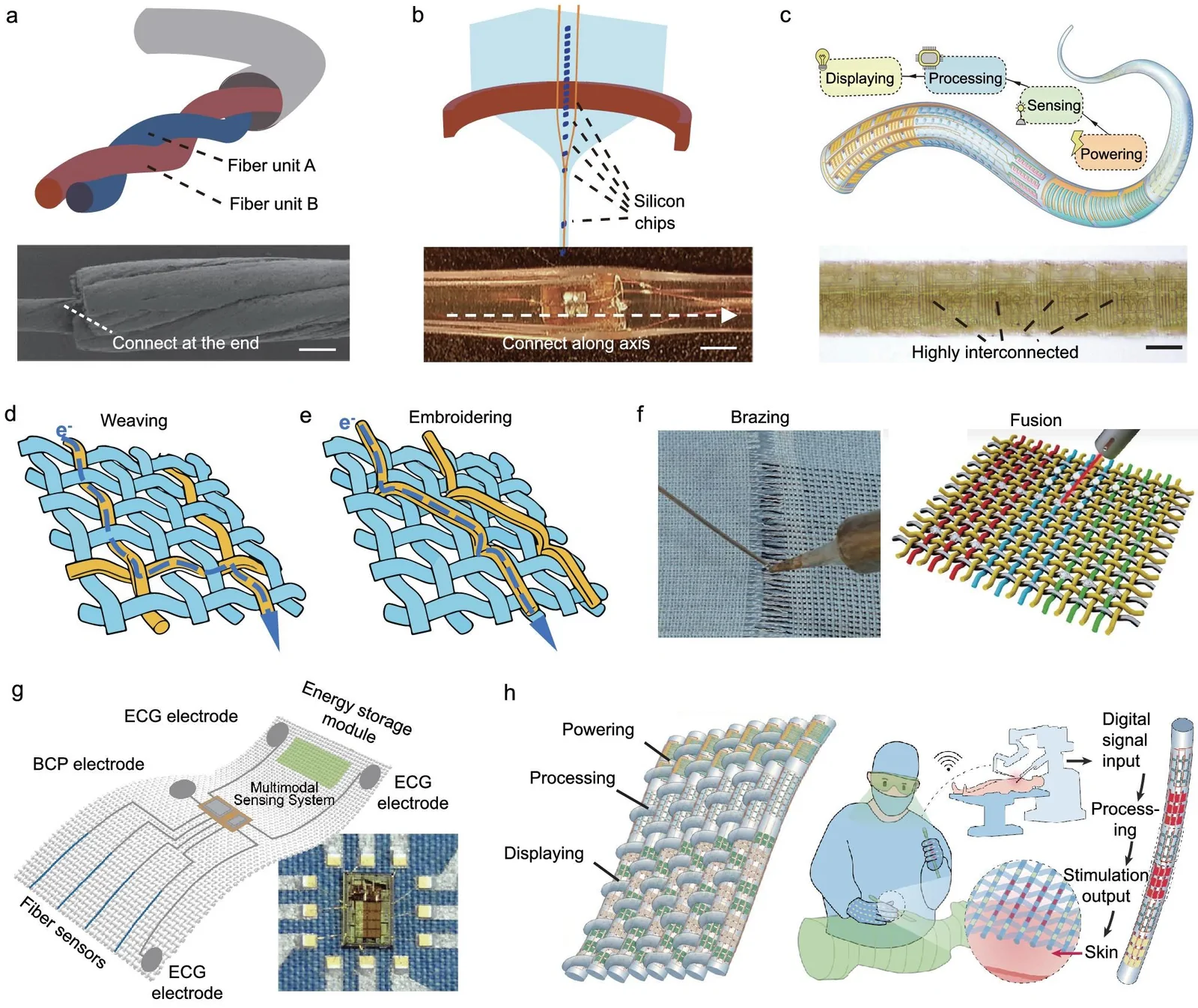
Integration strategies for integrated-fiber electronic systems. (a–c) Illustrations of methodologies to embed multiple functionalities into a single filamentary form, including (a) twisting of functional fibers into hierarchical yarns (adapted from [9]), (b) pre-embedding of micro-components during the fiber-drawing process (adapted from [233]), and (c) nano- or microscale fabrication directly on the fiber surface or within its core (adapted from [314]). These approaches represent the pursuit of the system-in-a-fiber paradigm, where power, sensing, and processing are natively integrated. (d and e) Techniques for scaling functional fibers into large-area systems *via* (d) industrial weaving and (e) precise embroidering. These methods utilize established textile manufacturing infrastructure to realize distributed electronic functions across 2D surfaces (adapted from [336]). (f) High-resolution photographs and schematics showing brazing and fusion welding techniques used to establish robust electrical connections between electronic threads (adapted from [337,338]). (g) Schematic and photograph of a system-on-cloth, where a silicon-based SoC is hybridized with a fabric-printed circuit, combining the high computational power of traditional chips with the conformability of textiles. (h) A conceptual schematic for next-generation flexible textile systems. These systems are envisioned to feature high-performance, self-powered, and flexible SoCs, enabling autonomous data processing and intelligence directly within the fabric matrix (adapted from [314,315]).

Progress in this area has followed a path from simple mechanical assembly toward precision manufacturing driven by innovations in fiber architecture and processing. Early, low-complexity integration involved axial twisting or winding to bundle distinct functional filaments (sensing, power, and communication) into a single composite yarn, achieving primitive intra-fiber integration (Fig. 15a). Twisting is fast and practical. It allows simple multimodal units (for example, co-twisting separate Ca2^+^, glucose, and H^+^ sensors into one filament). However, mechanical integration by twisting or winding typically limits the integration density to the order of tens of device units and struggles to scale to hundreds or thousands of modules. Thermal drawing has been developed as a more advanced integration strategy that increases integration density by pre-assembling chips and multi-material preforms, then drawing them into continuous, chip-embedded fibers (Fig. 15b). This technique leverages the high integration density and performance of commercial silicon chips to expand the functionality of integrated-fiber electronic systems greatly. Examples include thermally drawn fiber lighting (LEDs embedded and drawn into fibers) and single-fiber edge-computer systems produced by integrating microcontrollers, radios, and sensor ICs before drawing [233,321]. Nonetheless, embedding rigid silicon dies compromises the fiber’s inherent flexibility. Arranging chips in parallel along the fiber axis also fixes the circuit topology and constrains design flexibility for multifunctional applications.

More recently, *in situ* micro/nanofabrication on fiber substrates has emerged as a promising route to overcome the integration density barrier (Fig. 15c). To adapt ideas from silicon IC workflows, researchers now use conformal photolithography, low-temperature thin-film deposition, and direct printing to define high-precision circuit elements on fiber surfaces or within multilayer fiber stacks. *In situ* processing permits customized circuit layouts and dense device placement. At the same time, soft–hard hybrid architectures mitigate the stiffness introduced by metallic or ceramic components, preserving bending and tensile flexibility even when hundreds to tens of thousands of functional elements are integrated. Demonstrations include microfabricated transistors and logic on noncylindrical fibers, and the rolling of prepatterned elastic films into fiber geometries to realize closed-loop integrated circuits [322]. However, significant technical challenges remain. Curved, micron-scale geometries reduce lithographic resolution and make multilayer alignment sensitive to errors, and most polymeric fiber substrates cannot tolerate high-temperature or aggressive chemistries. To overcome these limitations, the field is advancing surface-conformal lithography tools, substrate activation (plasma and self-assembled monolayers), and low-temperature, low-damage microfabrication flows, while simultaneously developing fiber-compatible functional materials (organic semiconductors, 2D materials). Together, these innovations aim to push fiber-based integration toward higher density without sacrificing mechanical compliance.

### System-level integration across multiple fibers

At the fabric scale, system-level integration relies on a complementary toolbox. Printing, transfer, embroidery, and weaving are the core manufacturing paradigms for assembling integrated-fiber electronic systems. Printing and transfer methods, well established for planar flexible electronics, have been adapted for e-textile fabrication [323]. Printing builds conductive circuit traces directly on textile substrates (e.g. extrusion or screen printing of conductive inks) with feature resolution on the order of hundreds of micrometers. Transfer techniques use a pre-fabricate → release → laminate workflow. Flexible circuits are defined with high precision on a temporary planar substrate, then released and bonded to textiles using an adhesive layer. This route achieves higher fidelity than direct printing does. Embroidery and weaving provide complementary bottom-up assembly methods [324]. Conductive yarns are embedded or sewn into textiles to realize robust interconnects with process precision suitable for conventional textile circuits (Fig. 15d and e). Weaving enables large-scale assembly, while embroidery can overcome the texture constraints of the textile base and realize customized conductor layouts. In practice, hybridizing these approaches, using transfer/printing for high-precision modules and weaving/embroidery for coarse, robust interconnects, provides a pragmatic path toward scalable, manufacturable e-textiles that balance circuit fidelity, mechanical robustness, and compatibility with existing textile processes.

### Node-level fiber-to-fiber interconnection

While fabric-level techniques define the overall architectural layout, the system’s functional integrity hinges on the reliability of discrete fiber-to-fiber junctions. To establish these connections, electrical terminals are connected to the axial ends of each functional filament. These terminals form low-resistance electrical junctions with pre-embroidered conductive pads on the textile substrate through localized metallurgical soldering or conductive silver epoxy bonding [1,306]. Within this framework, a textile-based circuit network, constructed *via* industrial embroidery using high-conductivity silver-plated polyamide yarns, serves as the interconnecting backbone, effectively bridging the electrodes of discrete functional fibers into a cohesive system. To ensure long-term operational stability, silicone rubber encapsulation is applied at all interconnection nodes, providing a hermetic seal against dynamic mechanical deformation and repeated washing cycles [1,325]. This multi-level interconnection strategy aligns seamlessly with conventional textile manufacturing flows, providing a robust and scalable route for the assembly of sophisticated multi-fiber electronic systems.

### System-on-chip for integrated-fiber electronic systems

High-integration SoC solutions tailored for fiber devices are essential for expanding the application envelope of integrated-fiber electronic systems. In practice, such SoCs bundle multichannel, ultralow-noise analog front ends (AFEs) for electrical, optical, and biochemical acquisition, with on-chip demodulation, feature extraction, digital processing, and local memory, enabling rich edge pre-processing and reducing bus bandwidth and wiring needs [326]. Integration of low-power wireless interfaces (Bluetooth, near field communication, and body-coupled communication) minimizes wiring and enhances system compactness and user comfort (or fiber system friendliness) [327,328]. With careful power management, contemporary SoCs can achieve active-mode power in the microwatt range and sleep-mode power down to nanowatt levels, significantly extending battery life [329,330]. Coupling these SoCs with local energy-harvesting methods (triboelectric, photovoltaic, and biofuel cells, or body-coupled harvesters) or wireless power delivery provides a route to partially or fully self-powered integrated-fiber electronic systems suitable for long-term, low-maintenance deployment [331–333]. Practical demonstrations underscore the viability of this pathway. For instance, Schönle’s VivoSoC integrates multichannel AFEs and a quad-core microcontroller unit (MCU) for parallel optical/electrical processing and feature extraction [326]. Chen *et al*. demonstrated a 4 × 4 mm^2^ CHIW-SoC paired with CNT-fiber sensors to realize a flexible sweat-analysis patch that operates from NFC power, with ≈ 33 μW of running power, suitable for extended monitoring [334,335].

Establishing robust electromechanical interfaces and interconnection protocols is a cornerstone for achieving system-level fiber electronics [339]. Through advanced techniques such as laser micromachining, ultrasonic welding, and conductive bonding, researchers have enabled efficient signal transmission between heterogeneous fibers [331]. While silicon CMOS remains the workhorse for high-performance SoCs, its intrinsic planarity and rigidity have historically constrained textile conformability. Current strategies have mitigated this by shifting toward flexible carriers (polyimide, polyethylene terephthalate, polydimethylsiloxane) and robust interconnection methods, such as brazing and fusion welding (Fig. 15f), that ensure reliable mechanical and electrical bonding between rigid chips and soft fiber/textile substrates (Fig. 15g) [340–343]. Furthermore, device-level advances in ultrathin chips (<50 µm) and improved packaging have significantly increased pliability while reducing power draw [344–347].

However, despite these advancements, extrinsic interconnections and hybrid packaging strategies still rely heavily on physical contact points, which increase overall system volume and introduce stress concentrations under dynamic deformation. These interfaces emerge as a critical bottleneck for long-term system reliability. To realize high-performance, truly flexible in-fiber intelligence, SoCs must not only reduce power consumption without sacrificing signal integrity but also overcome reliability challenges arising from mechanical and environmental interference [335,348,349]. Modular assembly paradigms where functional units are connected *via* garment hardware such as buttons or zippers, offer a serviceable route to scalability [336]. Yet, they do not address the underlying structural heterogeneity. Consequently, the evolution of integration paradigms compels us to consider a fundamental shift through transcending these discrete, external inter-fiber connectivity and achieving true monolithic system integration within a single filament.

This pursuit of ultimate integration density has catalyzed the emergence of the single-fiber electronic system paradigm. By utilizing a multilayered spiral architecture, a fiber integrated circuit (FIC) was developed that marks a milestone leap from multi-fiber assembly to monolithic single-fiber integration (Fig. 15h) [314]. Distinct from traditional hybrid systems realized through weaving or lamination, this approach achieves *in situ* monolithic integration of energy modules (fiber batteries), sensing modules (glucose, temperature, and electrophysiological sensors), and high-density logic modules (FIC chips) by geometrically transforming spatial structures during continuous fiber fabrication. This system-in-a-fiber architecture not only eliminates redundant interconnections between heterogeneous units (e.g. significantly enhancing spatio-temporal resolution and data processing capabilities) but also ensures functional integrity under extreme mechanical loads (e.g. crushing by a 15.6-ton truck) *via* a modulus-heterostructure design. This paradigm shift not only reshapes the physical boundaries of fiber electronics but also steers the textile-as-a-computer vision from multi-level distributed architectures toward more efficient, compact, and autonomous intelligent units.

Fiber-electronic integration is advancing along two complementary pathways: internal, within-fiber integration (shifting from simple mechanical twisting to *in situ* micro- and nano-fabrication), and external, between-fiber system assembly (using printing, transfer, embroidery, and weaving). In both routes, interface engineering and system architecture are decisive. While the transition from discrete devices to single-fiber systems outlines a grand blueprint for the industrialization of fiber electronics, achieving commercial applications remains a challenge at the system level. First, manufacturing compatibility and yield management remain central challenges. As the integration dimension increases, a defect in any single functional module can cause the entire fiber to fail, demanding extreme precision and closed-loop feedback in fabrication processes. Second, system energy efficiency and thermal management become increasingly prominent in microscopic spaces; effectively dissipating the heat generated by high-density logic operations through multilayered encapsulation without compromising wearable comfort is a critical thermodynamic challenge. Finally, the lack of standardized interfaces and software ecosystems limits the synergy between heterogeneous fiber systems. A universal fiber bus standard and lightweight operating systems optimized for fiber hardware resources are urgently needed to support future complex, multimodal intelligent textile networks, thereby enabling deep deployment of fiber electronics in integrated-fiber electronic systems, implantable platforms, and related applications.

The technological trajectory detailed in the preceding sections, progressing from fundamental materials and the structural design of functional devices to the sophisticated orchestration of integrated systems, forms a complete, hierarchical framework for the future of fiber electronics. This bottom-up evolution ensures that every laboratory innovation is strategically aligned with the ultimate requirements of field-deployable applications. The synergistic integration discussed thus far provides a robust foundation for the multi-sectoral applications discussed in the following section. Specifically, the diverse material palette delivers the essential electronic performance and mechanical resilience required for various scenarios. The individual functional modules, including fiber-native sensors, power units, and logic circuits, serve as the organs of the system, enabling autonomous data acquisition and signal processing. Finally, the systemic integration strategies explored in this section act as the nervous system that transforms discrete filaments into cohesive, intelligent platforms. The comprehensive technological foundation established in the first four chapters now enables a paradigm shift across diverse fields: from pervasive, long-term healthcare monitoring and immersive human–machine interfaces to biomedical applications, among others. By bridging the gap between fiber science and systemic utility, this roadmap culminates in a vision where integrated-fiber electronic systems are seamlessly woven into the fabric of modern life. The following section will thus delineate how these technical milestones translate into socio-economic value, while identifying the remaining grand challenges (such as industrial scalability and sustainability) that must be addressed to realize the 2035 vision of a fully connected, intelligent textile world.

## APPLICATION FRONTIERS OF FIBER DEVICES

The technological evolution of fiber devices is now transitioning from fundamental material and component development to transformative real-world applications. This chapter surveys the most promising application frontiers where fiber-based technologies are demonstrating significant practical impact and where near-term breakthroughs are anticipated. It focuses on three interconnected domains that represent the cutting edge of fiber electronics: wearable electronic textiles that integrate sensing, display, and computational capabilities directly into garment platforms; fiber devices for energy management, environmental sensing, and human–machine interfaces; and implantable and epidermal fiber systems for continuous health monitoring and targeted therapy.

### Wearable electronic textiles

Wearable electronic textiles constitute a new generation of smart systems in which flexible fiber devices serve as the basic electronic units and interconnects. By integrating electronic functions while preserving the intrinsic wearability of textiles, these systems embed sensing [2,4], actuation [350], energy harvesting/storage [7], computation and communication modules [1,233,351,352] into multiscale textile architectures, from individual fibers and yarns to fabrics, using processes such as spinning, weaving, embroidery, and coating [353]. This fiber-as-device, fabric-as-system paradigm removes the barrier between rigid electronics and soft textiles, enabling conformal, breathable, and comfortable garments with embedded intelligence. Early engineering routes to this vision were pioneered by the Wearable Motherboard™/Smart Shirt concept from Georgia Tech in 1997 for integrated physiological sensing [354] and the MIT Media Lab’s E-broidery work in 2000, which established conductive-yarn-based circuit construction on fabrics and laid the foundation for contemporary fiber-device electronic textiles [355].

**Figure 16.**
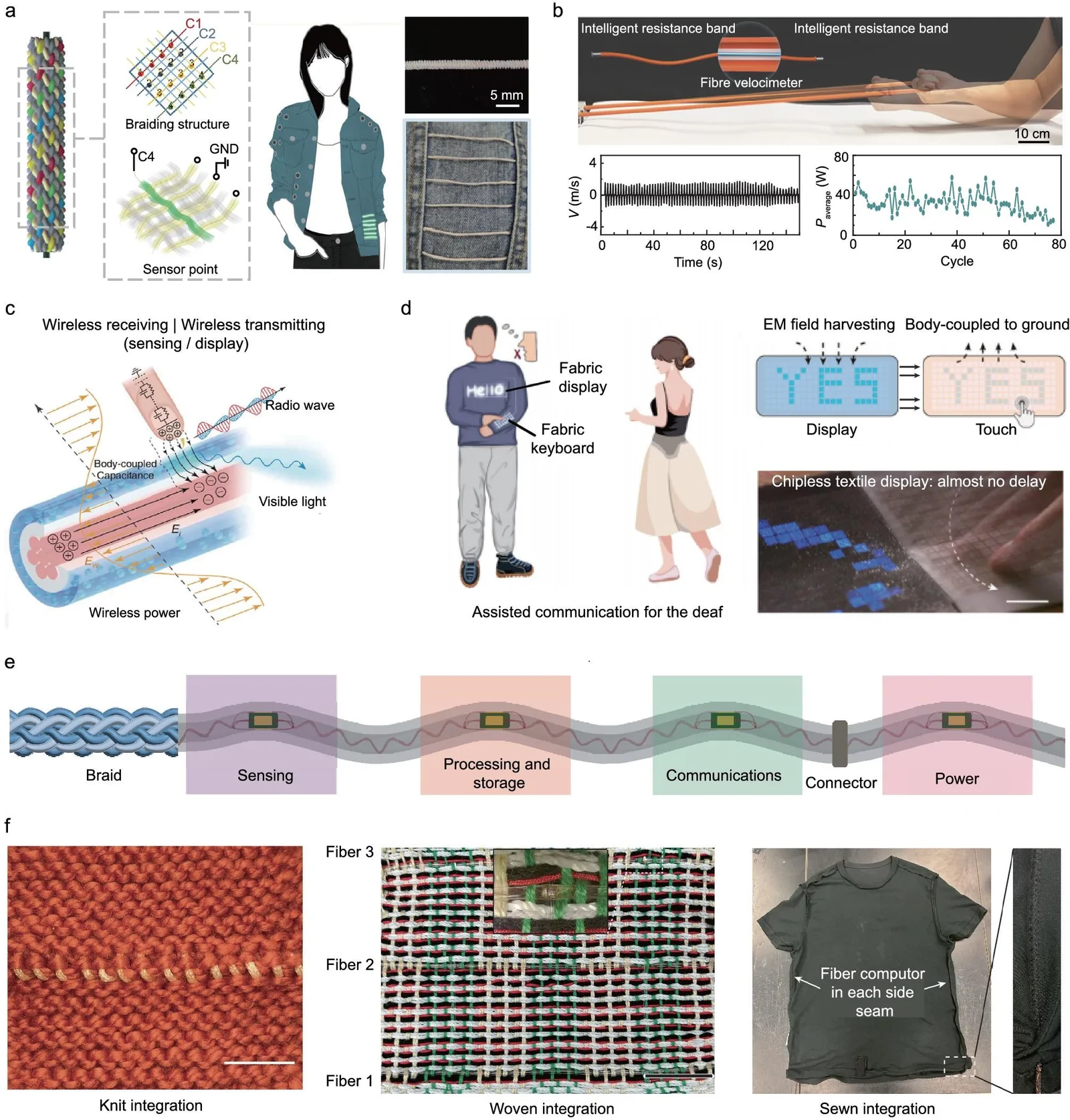
Multifunctional wearable electronic textiles. (a) A braided electronic cord designed for the seamless remote control of IoT devices (adapted from [356]). (b) A fiber velocimeter integrated into sports apparel for the real-time dynamic digitization of resistance training, illustrating the transition of textiles into active digital interfaces (adapted from [357]). (c and d) Demonstration of a cohesive fiber system capable of simultaneous powering, sensing, displaying, and interacting. By moving away from traditional von Neumann architectures, these systems achieve localized, low-latency processing and feedback within the textile itself (adapted from [351]). (e and f) High-level integration of miniaturized electronic modules within a single fiber. Through the encapsulation of discrete functional units, these fibers achieve complex system behaviors while maintaining the mechanical flexibility and tactile comfort of standard textile yarns (adapted from [321]).

Over the past decade, the field has matured along a hierarchical pathway, from single-fiber functionalization and fabric structuring to system integration, where advances at each scale compound to increase overall capability and usability. Single-fiber hetero-integration is the foundation for high-density functional textiles, and several groups have reported notable breakthroughs. An imperceptible, integrated, intelligent braided electronic rope was developed by co-weaving conductive and flexible substrate fibers (Fig. 16a) [356]. Combining multi-sensor feature-fusion algorithms, precise touch localization, regional sensing, and complex-gesture recognition enabled core interaction accuracy >96%, providing a prototypical route for lightweight interactive fabrics. An intelligent fiber velocimeter has also been engineered, which achieves a velocity measurement accuracy of over 95% in the 0–2.5 m s^−1^ human motion range for dynamic sensing in resistance training (Fig. 16b) [357]. A non-von-Neumann smart fiber was proposed that integrates a silver-plated nylon antenna, a dielectric layer, and an electric-field-sensitive emissive shell in a sheath-core geometry (Fig. 16c and d) [351]. Single fibers simultaneously harvest electromagnetic energy and sense and transmit signals, enabling touch-activated emission and wireless command transmission without external chips or batteries. The team at MIT demonstrated high-level heterogeneous integration within a single fiber by encapsulating a low-power microcontroller, a high-density addressable LED array, and a flexible pressure-sensor array. The woven fabric produced from these fibers supports synchronized gesture recognition and real-time heart rate visualization while tolerating > 50% tensile strain (Fig. 16e and f) [321].

Beyond single-fiber capabilities, fabric-level co-design enables practical wearable systems by balancing multifunctionality with wearer comfort. Ion-conductive silk-based smart textiles were developed that extend sensing from physical parameter monitoring to hazard response and human–machine interaction. This protective–interactive design preserves silk’s skin-friendly breathability while adding an active response, opening new safety dimensions for wearable devices [260]. Another example is the hierarchical optical-metamaterial textile that partitions the solar spectrum into ultraviolet, visible and near-infrared, and mid-nfrared bands across layered metamaterials. Compared with commercial white cotton, this layered fabric achieves broadband spectral control with ~92.4% solar reflectance and ~94.5% mid-IR emissivity, lowering the skin temperature by ~5°C [358]. A SonoTextile platform that integrates guided acoustic channels into a fabric architecture to deliver robust, acoustically enabled functions was recently reported. When combined with wearable power and wireless modules, such acoustic textiles can implement closed-loop sense—feedback—intervene medical garments that mitigate material degradation and system-complexity bottlenecks [264]. System-level integration seeks to close the gap from isolated functional fibers and discrete functional fabrics to monolithic and intelligent textiles by deeply embedding modules and achieving cross-module synergy. A computational fabric concept was proposed, in which textile-level integration of 5G connectivity and on-fabric AI enables energy harvesting, local data storage, machine learning, and closed-loop actuation for applications in healthcare, aerospace, and the metaverse [248]. A landmark demonstration was the integration of display–touch–power into a fully flexible textile by forming micro-light emitters at fiber cross-points, producing a large-area display textile with ~5 × 10^5^ luminous pixels, an important step toward truly textile-native user interfaces and immersive wearable systems [1].

The form factor of fiber devices and systems makes them ideal building blocks for next-generation smart spacesuits that go beyond passive protection to integrate life support, sensing, power, and actuation in a single conformal garment [359]. On-garment energy harvesting and storage (fiber solar cells and fiber-scale lithium storage) can be used in local power systems [360,361]. Embedded artificial muscle fibers provide soft, assistive actuation to augment joint motion [362,363]. Reparable, airtight barrier layers enable self-healing and long-term suit integrity [364]. Crucially, these functions can be woven into layered, body-integrated systems [365]. Flexible display and controller fibers in the outer layers provide distributed human–machine interfaces [1]. Inner sensor layers continuously monitor physiology and infer the wearer’s intent for adaptive support [248]. Integrated thermal control, air management, power routing, sensing, and actuation modules converge to form a compact life-support architecture that is lighter, more resilient, and far more capable than conventional suit designs [366].

Key technical challenges remain before wearable electronic textiles can be deployed at scale. The first is single-fiber multifunctional integration and fabric-scale coordination. Packing multiple functions into a single filament often results in electrical crosstalk, thermal and mechanical incompatibilities, and complex routing requirements. At the production scale, the arrangement, interconnection, and coprocessing of heterogeneous functional fibers become increasingly difficult. Practical mitigations include spatially separated layered or coaxial architectures that isolate sensing, conduction, and barrier layers and standardized module interfaces that simplify assembly and testing. The second is the materials versus mechanics and stability. Surface functionalization or filler loading used to increase conductivity, energy storage, or sensing typically undermines intrinsic toughness, fatigue resistance, and environmental stability. Approaches that preserve mechanical integrity include covalent grafting, graded nanoscale filler distributions, interpenetrating polymer networks, and binder chemistries that lock functional phases without embrittling the host fiber. The third goal is to preserve softness and wearer comfort. Embedded electronics tend to stiffen textiles and degrade drape and touch. Retaining garment comfort requires ultralight, compliant conductors (nanofibers, metal nanowires), elastic conductive composites, ultrafine yarns, and knit/mesh constructions that distribute stiffness and maintain breathability. Fourth, yield, cost, and quality control for industrialization should be considered. Multi-material, multi-step processes accumulate tolerances and drive-up scrap rates, but the field currently lacks unified quality-traceability frameworks and textile-specific test standards. Therefore, realizing scalable adoption demands manufacturing solutions compatible with existing textile infrastructure (e.g. continuous spinning and coating, automated weaving/embroidery, roll-to-roll transfer, and inline optical/electrical inspection) as well as process-level closed-loop control, statistical quality metrics, and standardized test protocols for mechanical durability, washability, and per-meter electrical/functional uniformity. Addressing these four areas in parallel, including device architecture, materials chemistry, textile design, and manufacturing infrastructure, will be essential to translate laboratory demonstrations into comfortable, reliable, and cost-effective wearable electronic textiles.

### Fiber devices for human–machine interaction systems

Human–machine interactions (HMIs) built from fiber devices promise a qualitatively different interface paradigm: body-conformal, lightweight fabrics that sense, wirelessly communicate, compute, and actuate at the skin–textile boundary. Compared with rigid peripherals, fiber-native HMIs offer superior mechanical compliance, continuous multimodal sensing, and the potential for on-node preprocessing and low-latency feedback, properties that make them especially attractive for health monitoring, assistive technologies, and immersive augmented reality or virtual reality (AR/VR). This section summarizes recent progress across the four core stages of fiber-based HMI systems (signal acquisition, namely the input, wireless transmission, information processing, and feedback control, namely the output) and highlights the technical priorities required to close the loop between people and intelligent platforms [5,6,367–370].

### Signal acquisition (input)

**Figure 17.**
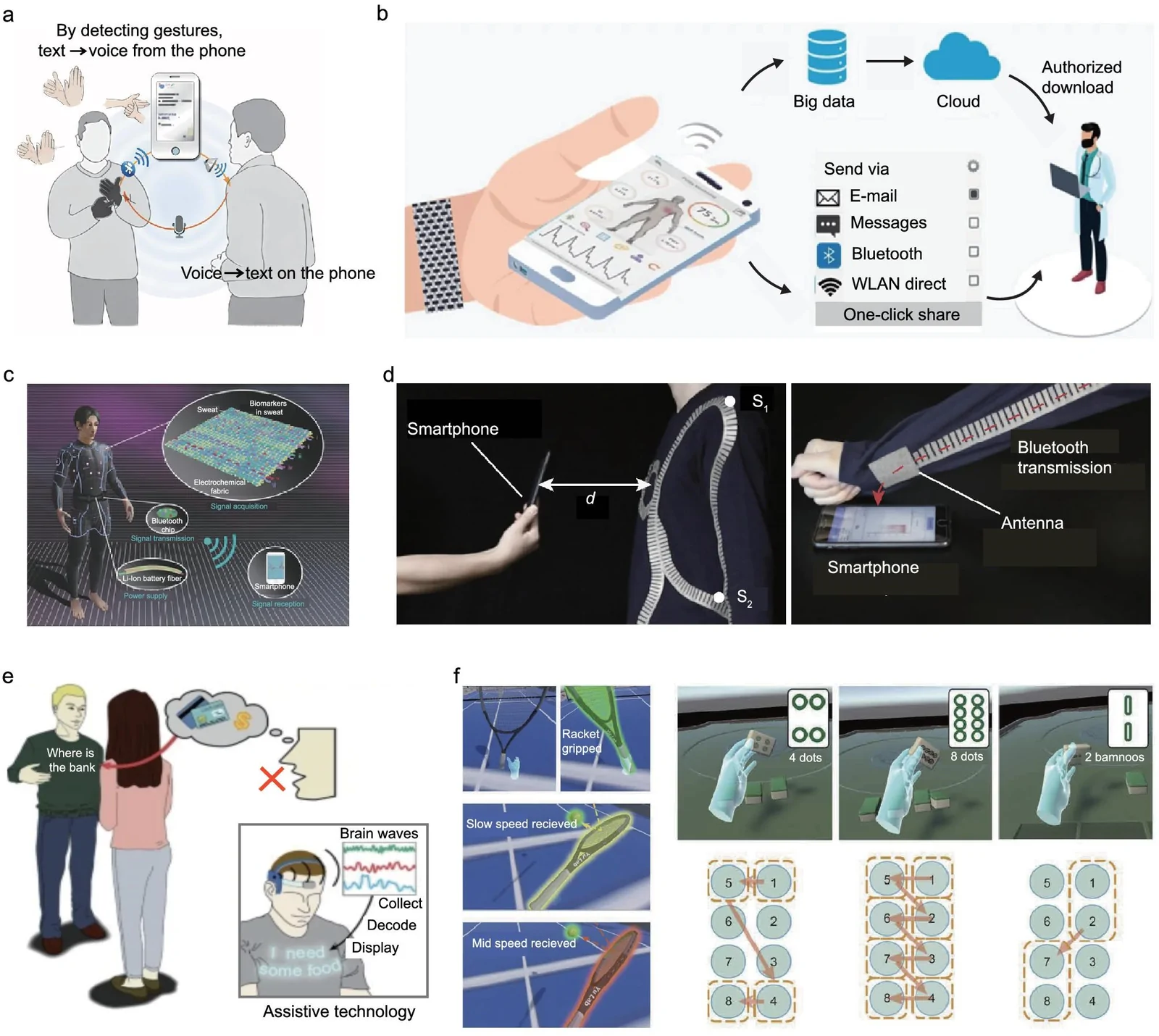
Integrated-fiber electronic systems in human–machine interface (HMI) applications. (a) Gesture recognition systems that support assistive interaction for individuals with mobility impairments (adapted from [376]). (b) Integration of bioelectrical acquisition (e.g. EMG/ECG) with machine-learning pipelines for autonomous, long-term personal health monitoring (adapted from [377]). (c) Wearable electrochemical fabrics designed for the continuous monitoring of biomarkers in sweat (adapted from [379]). (d) Textile NFC systems that enable wireless power and data transfer across the body *via* inductive coupling, eliminating the need for bulky batteries or wired connections (adapted from [384]). (e) A visual-feedback smart textile that translates gestures into text or light signals, assisting speech-impaired users in communication (adapted from [1]). (f) A tactile-feedback system for virtual reality (VR), providing users with immersive haptic sensations through fiber-based actuators (adapted from [114]).

Fiber sensors serve as the primary source of information for HMIs and must capture physiological and environmental signals with high fidelity and temporal resolution. Mechanical fiber sensors (strain/pressure) are widely used for pulse, respiration, and joint-motion monitoring [371]. Extracting these signals enables higher-level functions such as information encoding [372], gesture translation and texture recognition [373–375], which, in turn, support assistive interactions for special populations (Fig. 17a) [369,376]. Conductive fiber electrodes provide low-impedance, conformal contact for continuous acquisition of bioelectrical signals (EEG, ECG, and EMG). When they are combined with appropriate machine-learning pipelines, they can drive precise, real-time HMI responses (Fig. 17b) [377]. In parallel, chemical fiber sensors complement electrical and mechanical modalities by continuously tracking biochemical markers (electrolytes, lactate, and metabolites) in sweat or saliva, providing real-time physiological feedback that is highly valuable for interactive health systems (Fig. 17c) [98,378,379]. Optical fiber sensors, such as fiber Bragg gratings (FBGs), are also important for HMI and rehabilitation. FBGs can monitor blood pressure, respiratory rate, and body posture, and enable systems to adapt therapeutic parameters accordingly [380–382]. Optical sensors can simultaneously capture multiple parameters (e.g. strain and temperature) by demodulating wavelength shifts, resulting in a multimodal perceptual capability that enriches decision logic and supports more sophisticated interactions [383].

### Wireless transmission

A key challenge across the HMI chain is the reliable, efficient transmission of multimodal signals from distributed fiber sensors to back-end processors while preserving mobility. Conventional wired interconnects (metal conductors) suffer from instability, limited bandwidth, high power consumption, and connector aging. As a result, integrating wireless communication functions directly into fiber architectures has become an important research direction for the construction of low-power, robust transmission frameworks [321,385]. Demonstrations include fiber loop antennas made from conductive yarns or metal filaments to establish closed-loop links with terminals [353] and textile NFC networks based on inductive coupling for short-range wireless power and data transfer around the body (Fig. 17d) [384]. These approaches decentralize the topology, increase device form-factor flexibility, and broaden the application scenarios for HMI fabrics.

### Information processing

Closing the perception–action loop requires in-fabric processing to reduce latency and bandwidth. Fiber-compatible compute primitives, notably memristive and other in-memory devices, enable massively parallel computation at the textile node [315,386]. Crossbar arrays and memristive fiber networks have demonstrated basic logic and compute-in-memory capabilities, collapsing the path between sensing and decision and enabling on-textile preprocessing, feature extraction, and low-latency classification of physiological events (e.g. seizure detection). Coupling such primitives with ultralow-power SoCs and energy-aware architectures supports real-time operation while minimizing data transfer and preserving user privacy.

### Feedback control (output)

Feedback fibers convert algorithmic decisions into perceptible, closed-loop responses and encompass visual, tactile, and auditory modalities [367,387,388]. Visual feedback remains the dominant human sensory channel (over 80% of perceptual input). Seamlessly weaving light-emitting fiber elements into textiles enables wearable displays and status cues that translate abstract information into intuitive visual messages, thereby closing the human–machine information loop effectively for speech or communication impairments (Fig. 17e) [1]. Tactile feedback fibers transduce digital commands into mechanical, thermal, or electrical stimuli, significantly improving the immersion, responsiveness, and reliability of human–machine interactions. Examples include fluid-driven fibers that produce rapid linear actuation and strong force feedback [389] and electrically stimulated textiles that deliver localized dynamic touch on the palm or other body sites [390]. Mapping sensory data into spatial vibration patterns across the body provides rich virtual-touch interfaces for many scenarios, such as assisting visually impaired or hearing-impaired users in perceiving obstacles or experiencing content in virtual reality (Fig. 17f) [114]. Auditory feedback fibers add a time-dependent, spatially structured information channel. Triboelectric acoustic fabrics both sense and reproduce sound and, when paired with deep-learning models, enable voice control, cloud access, and conversational interaction with AI agents [391]. Magnetically actuated vibrating fibers can also serve as wearable sound transducers [392]. Modern HMI systems, therefore, combine woven visual emitters, haptic actuators, and textile-integrated audio to enable immersive, bidirectional, multisensory interactions.

Fiber-device and fabric-integration approaches have produced substantial advances in sensing and feedback, but communication and computation fibers remain at an earlier stage. Improving their performance places greater demands on micro- and nanostructure design, heterogeneous material integration, and advanced textile manufacturing. Closing that gap requires tightly coupled, cross-disciplinary work spanning functional materials, device–fabric coupling, and hardware/software co-design so that communication and computation elements can meet textile constraints (stretchability, washability, low profile) without sacrificing throughput, latency, or energy efficiency. Concrete near-term priorities are (1) developing multimodal, fabric-compatible sensor materials and robust fixation chemistries; (2) optimizing device–textile mechanical coupling to preserve signal fidelity under deformation; (3) scaling edge-compute elements (memristive arrays, low-power SoCs) within textile constraints; and (4) designing reliable wireless, power, and data architectures. Achieving closed-loop integration of sensing, transmission, processing, and feedback at the fiber level will enable intelligent fabrics that are autonomous, adaptive, and environmentally integrated, opening new routes for natural human–machine interaction.

### Medical applications and health management

Medical and health-management applications are among the most strategically important targets for functional fiber materials, devices, and integrated fiber systems. Fibers offer tailorable, multiscale architectures, facile functionalization, and bio-friendly interfaces, making them ideal substrates for biomimetic tissue microenvironments and devices that emulate physiological processes. Because fiber platforms can be soft, breathable, low-mass, and highly conformal, they enable closed-loop workflows for continuous monitoring, early warning, and precise intervention, opening the way for personalized regenerative medicine, minimally invasive therapy, and continuous clinical care. Recent progress in the use of fiber materials and devices for physiological monitoring, diagnosis, and therapy is summarized below.

**Figure 18.**
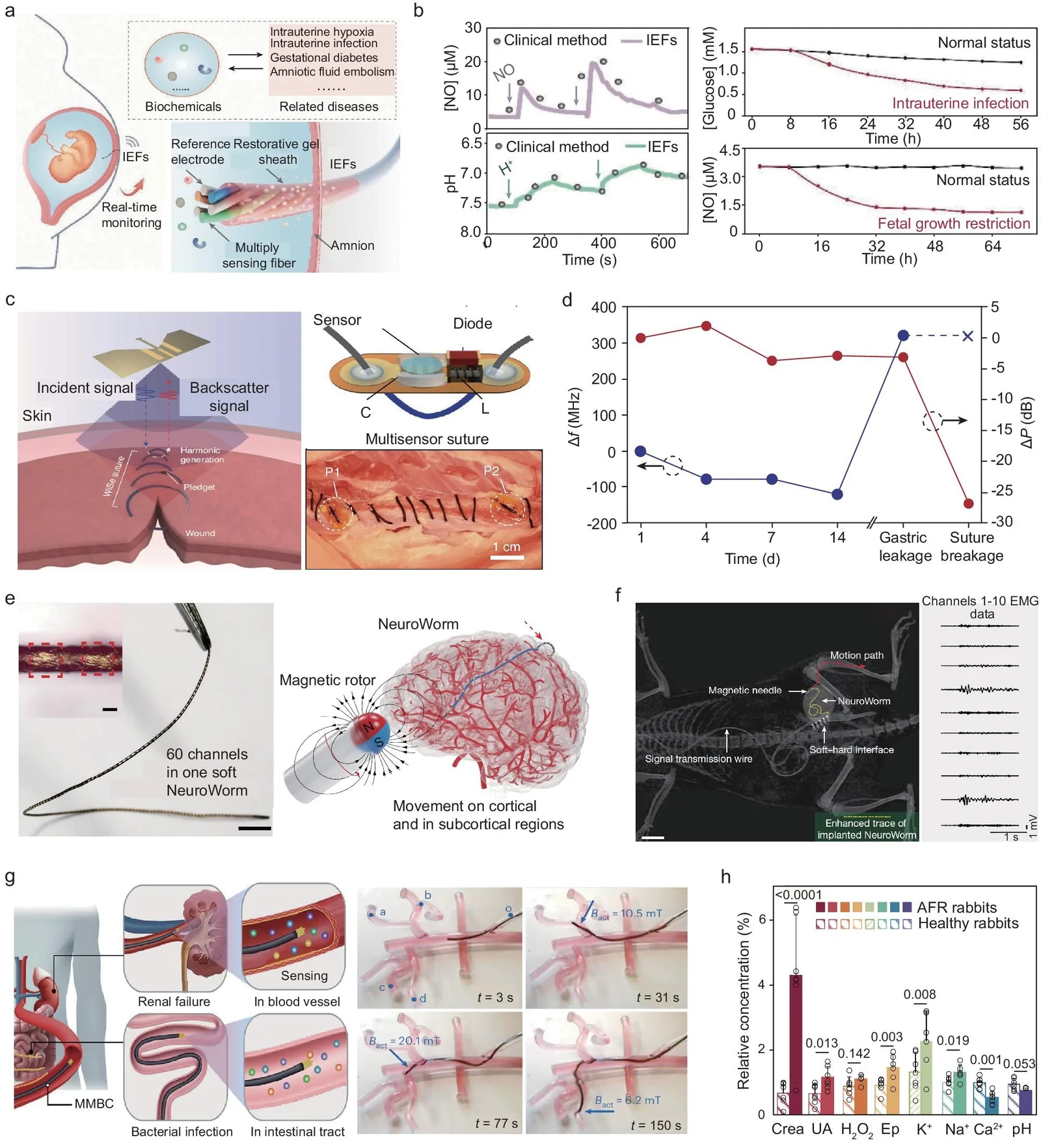
Fiber devices for medical and healthcare. (a and b) Application of an implantable fiber device for the long-term, high-fidelity tracking of amniotic fluid indices and infant respiratory patterns, providing a safer and more continuous alternative to traditional ultrasound or bulky sensors (adapted from [300]). (c and d) A fiber-based sensing network integrated into surgical dressings to monitor wound integrity and detect postoperative microleakages in real time, facilitating early intervention (adapted from [397]). (e and f) Soft, magnetically actuated neural fiber electrodes that can be precisely steered to minimize tissue damage during implantation (adapted from [3]). (g and h) A magnetically controlled microfiber robot capable of navigating through complex vascular or neural networks for targeted drug delivery or minimally invasive surgery (adapted from [398]).

Non/minimally invasive physiological monitoring. Owing to their light weight, breathability, and tissue compliance, fiber devices have advanced from single-signal acquisition to continuous, multimodal monitoring for noninvasive health management. Early fiber devices based on metallic wires suffer from poor wearing comfort because of their rigidity and are therefore limited in application. The introduction of liquid metals, conductive polymers, and carbon nanomaterials has transformed the field. Modern soft-fiber devices now support stable, continuous acquisition of bioelectrical signals (ECG, EMG, etc.) and high-resolution monitoring of biochemical and physiological parameters, including *in vivo* metabolites, amniotic fluid indices, and neonatal respiration. For example, a stretchable, breathable ECG patch was fabricated by photolithographic transfer and fiber spinning, enabling high-precision wireless intra- and post-operative ECG monitoring [393]. Soft, breathable fiber sensor systems have also been applied to high-resolution assessments of knee swelling during rehabilitation, where good biocompatibility supports long-term stability [394,395]. In perinatal care, flexible fiber sensors can continuously track amniotic fluid indices and infant respiration, supporting early warning for sudden infant death syndrome (SIDS) (Fig. 18a and b) [300,396]. Smart beds that fuse multimodal sensing with edge computing, collecting ECG, body temperature, and limb motion synchronously, improve data throughput and real-time analytics for clinical monitoring.

Fiber devices are also driving surgical and interventional techniques toward more minimally invasive, precise, and intelligent paradigms [294,399–401]. In therapeutic applications, smart sutures integrating electrical stimulation and drug-release functions enable real-time monitoring of wound integrity and microleakage while promoting tissue healing at medical-grade efficacy levels (Fig. 18c and d) [397,402–405]. Multifunctional fiber catheters that integrate microelectrodes and optoelectronic probes can continuously monitor local myocardial pH and lactate levels or detect early thrombus formation *via* impedance spectroscopy [399–401]. A soft, stretchable, magnetically actuated neural fiber electrode was developed for dynamic, stable *in vivo* electrophysiological monitoring (Fig. 18e and f) [3]. Using 4D multi-channel printing, a flexible magnetic fiber catheter was produced with multi-channel sheath structures that permit simultaneous, *in situ* multichannel sensing of metabolites and ions [406]. High-density flexible electrode bundles can be implanted into deep brain structures to stably record activity from targeted neuronal ensembles over long periods, providing critical data for the precise diagnosis and treatment of neurological disorders such as Parkinson’s disease.

Medical fiber robots act as distal physiological interfaces and as proximal actuators/sensors, enabling active navigation, laser ablation, and embolization within the body, opening new routes for minimally invasive surgery. A magnetically guided fiber robot was reported for neurovascular thrombectomy, capable of reaching intracranial clots with high targeting precision [407]. Magnetically actuated microfiber robots with high maneuverability and reconfigurable morphologies can traverse submillimeter vasculature against flows up to 200 mm s^^−1^^ and perform cascaded embolization operations (Fig. 18g and h) [398]. Robots combining magnetic guidance with onboard micro-lasers can autonomously navigate to lesions within vessels or cavities and perform precise laser ablation or release preloaded embolic agents to occlude tumor-feeding vessels, achieving minimally invasive, combined diagnosis-and-therapy operations [408].

In regenerative medicine, integrated-fiber electronic systems have progressed from passive structural scaffolds to active platforms capable of dynamically modulating tissue microenvironments. Fiber scaffolds that incorporate electrically, magnetically, thermally, or optically responsive units enable active control of electrophysiological activity, inflammatory responses, and targeted drug delivery. For example, woven fiber networks can recapitulate the spatial architecture of the vasculature and bile ducts within liver lobules or nephron units, supporting coupled structural and metabolic functions. Conductive fiber networks can emulate native electrophysiology to promote the maturation of neural or cardiac tissues. Thermo-responsive or enzyme-sensitive fibers can detect local inflammation and trigger on-demand drug release. Injectable short-fiber systems that exploit shear-thinning rheology provide minimally invasive *in situ*-forming scaffolds with high loading capacity for functional molecules, enabling targeted repair in central nervous system injury or myocardial infarction. For central nervous system disorders such as Alzheimer’s disease and Parkinson’s disease, noninvasive delivery routes, e.g. intranasal administration of surface-functionalized short fibers loaded with neurotrophic factors (brain-derived neurotrophic factor and nerve growth factor), immune modulators, or RNA therapeutics, can exploit the olfactory pathway to bypass the blood–brain barrier and concentrate payloads at diseased sites [409]. Preclinical models have been used to promote neural regeneration and functional recovery.

Although fiber materials and devices have advanced wearable monitoring and regenerative toolkits, substantial hurdles remain before clinical translation. Key gaps include demonstrable long-term implantable reliability (stable device–tissue interfaces, anti-fouling, and failure-mode mitigation), robust multimodal integration at a clinically relevant scale (co-packaging of sensing, stimulation, drug release, and onboard processing), and validated closed-loop therapeutic performance under realistic physiological conditions. Future efforts should prioritize durable bio-integration strategies and accelerated biostability testing (mechanical fatigue, chemical/enzymatic degradation, and sterilization compatibility), scalable manufacturing routes for multifunctional integrated-fiber electronic systems, and rigorous preclinical pipelines that combine chronic large-animal studies, standardized functional end-points, and regulatory-grade safety evaluations to de-risk translation into human trials.

### Industrial translation of fiber devices

The industrial translation of fiber electronics is strategically important because it can reshape the form factor of electronic products and accelerate technology upgrades across smart wearables, healthcare, and advanced manufacturing. By moving electronic functionality out of rigid boxes and chips and into fibers and fabrics [336], fiber electronics exploit inherent textile compatibility and spatial extensibility to integrate electronics seamlessly with the human body and built environments [410]. This morphological shift also aligns electronics with a textile-manufacturing paradigm that supports large-volume, low-cost production and thus the broad diffusion of fiber-enabled products across consumer and industrial markets.

**Figure 19.**
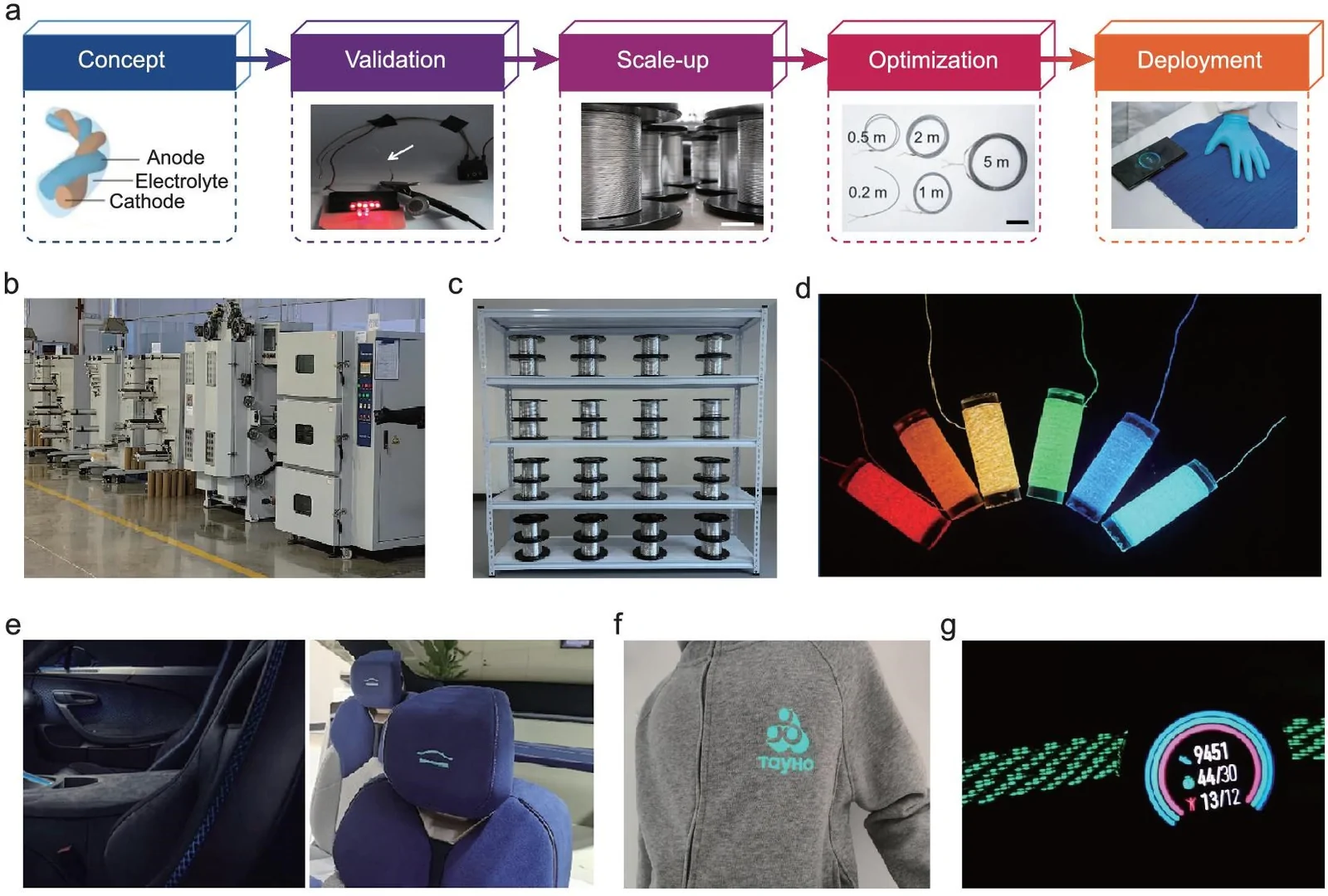
Industrial translation and mass-manufacturing of fiber devices. (a) A systematic roadmap outlining the transition from laboratory-scale fundamental research to pilot production and eventually to full-scale industrial manufacturing of fiber devices. (b and c) Photographs of a pilot production line for fiber lithium-ion batteries, demonstrating the feasibility of kilometer-length manufacturing while maintaining high energy density and safety standards. (d and e) Examples of mass-manufacturable electroluminescent fibers and integrated display-textile products, highlighting their readiness for integration into the global textile and fashion supply-chain.

To move from prototypes to industry, a staged, multi-path strategy is required that follows the logic concept: proof of principle, continuous construction, performance optimization, and industrial application (Fig. 19a). Practical policy and deployment actions should accelerate four linked processes. First, catalyze technology transfer and incubation to shepherd lab innovations into startups and initial scale-ups. Second, form industry–academia–research–user consortia that set roadmaps and coordinate work on shared technical bottlenecks. Third, large textile and electronics manufacturers should be engaged as industrial anchors to leverage mature supply chains and pilot production capabilities. Fourth, public and private capital should be mobilized through targeted grants and industrial funds to de-risk early scaling and create an innovation ecosystem. Together, these measures help convert breakthroughs into capacity and, in turn, into market penetration.

Commercial translation requires reconciling high electrical performance with textile-grade attributes. A commercially viable fiber device must combine reliable performance, manufacturing compatibility, and clear application fit. Reliable performance means stable operation at low voltage and resistance to washing, abrasion, and environmental exposure. Manufacturing compatibility requires deep technical alignment with yarn- and weaving-based processes to achieve low-cost, high-throughput production. Clear application fit means meeting the specific functional and regulatory requirements of target markets, such as medical monitoring or wearable interactions [411]. In practice, successful translation involves integrated materials–process–device solutions. Examples include spinnable conductive materials, intra-fiber series/parallel architectures, and dynamic encapsulation schemes that bridge lab prototypes and industry-grade products.

Several commercialization successes already signal practical viability. In thermal management, graphene/CNT-fiber–based flexible heaters [412,413] have reached scale and penetrated premium-heated outdoor apparel and therapeutic support markets. Market research indicates that the global 12 V heated apparel market will increase from USD 404 million in 2024 to USD 613 million by 2031 [414]. In health and medical applications, conductive-fabric sensors are widely used in fitness and rehabilitation (e.g. heart rate shirts and EMG garments). The global market for flexible fiber sensors is expected to reach USD 5.0 billion by 2025 [415]. Conductive fiber electrodes for continuous glucose monitoring have entered clinical validation, with unit costs falling to approximately CNY 400, accelerating telemedicine and chronic disease management [416]. In terms of energy and photovoltaics, fiber lithium-ion batteries have reached pilot production lines by Tayho Advanced Materials Group Co., Ltd. (Fig. 19b and c) [6,417]. In display textiles, electroluminescent fibers have achieved continuous, ultrafine (0.2–0.5 mm, controllable) [418], highly flexible roll-to-roll production with a luminance of approximately 200 cd·m^−2^ and continuous emission lengths of 100 m, together with robust waterproofing and insulation that meet outdoor safety requirements (Fig. 19d). A few display-textile products have already been deployed in specific segments of the automotive industry (Fig. 19e). Collectively, these industrial outcomes generate direct economic value and create positive feedback among technology maturation, supply-chain development and new application markets.

Despite significant progress, central technical tension remains to preserve long-term device stability and reliability while high-throughput, low-cost textile manufacturing is adopted. First, active device materials still require improvements in flexibility, washability, and environmental robustness to meet end-use durability requirements. Second, conventional electronic manufacturing methods do not yet integrate seamlessly with industrial weaving and knitting, thereby constraining scale and increasing costs. Third, the field lacks harmonized test methods, qualification standards, and cross-sector collaboration mechanisms, which slows iterative improvement and reduces market confidence.

Commercializing fiber electronics, therefore, links frontier science to the broader economy and can create new productivity platforms. If these technical and institutional barriers are addressed, fiber electronics can enable transformative markets in health monitoring, energy, aerospace, and consumer products. Beyond commercial value, successful translation will deepen cross-disciplinary collaboration among materials science, textile engineering, and electronics, reshape value chains, and establish an end-to-end ecosystem from basic research to high-value manufacturing. As a result, industrializing fiber devices is a strategic lever for sustainable development, improved public health, and broad technological and economic growth.

In summary, the application landscape detailed in this section represents only some of the realizations of the technological milestones established in the preceding chapters. These scenarios are not merely future possibilities but are the primary drivers of a fundamental paradigm shift: moving from material-push discovery to application-pull engineering. By aligning molecular- or nanoscale engineering with device and system integration in these high-impact sectors, the roadmap provides a definitive blueprint for the socio-economic translation of fiber electronics. The guiding significance of the discussions on industrialization and translation cannot be overstated. We have identified that the leap from laboratory-scale proof-of-concept to industry-grade reliability is the most critical hurdle for the next decade. The actionable pathways proposed, including the standardization of washability protocols, the development of high-throughput roll-to-roll manufacturing, and the integration of circular economy principles, serve as a prioritized checklist for the research community and industrial stakeholders. These elements can ensure that the field of fiber electronics moves toward a mature, sustainable industrial ecosystem rather than remaining a collection of disparate academic curiosities. Therefore, in this review, we aim to reach the conclusion that, through cross-disciplinary orchestration, bridging polymer science, microelectronics, biomedical engineering, textile engineering, and data science, we can overcome the remaining yield and complexity gaps.

## CONCLUSION AND PERSPECTIVE

### Convergence toward integrated-fiber electronic systems

The advances in fiber materials support a broad device palette, including energy-storage and conversion fibers, light-emitting and display threads, wearable and implantable fiber sensors, and emerging data-processing fibers, and enable integration across scales from single multifunctional filaments to woven, multi-fiber textiles. Early demonstrations in wearable electronic textiles, human–machine interfaces, healthcare monitoring, extreme-environment systems, and pilot industrial deployments illustrate both the versatility of fiber devices and the pressing need to move the field beyond prototype demonstrations. Several unifying insights emerge from the field’s rapid progress. First, the fiber form factor should be treated as an enabling design dimension rather than a constraint. The one-dimensional geometry, namely its curvature, axial anisotropy, and hierarchical porosity, reveals optical, thermal, and transport phenomena that planar formats cannot reproduce, and these features must be exploited intentionally. Second, multifunctionality is not achieved by simple stacking. Naive layering typically results in fragile interfaces and rapid performance degradation. Embedding storage, sensing, actuation, or display functionality into a single filament requires materials and interface designs that operate synergistically. Third, many demonstrations boast impressive single-device metrics, yet few translate to reproducible, continuous production with stable yields or to validated durability under real-world mechanical, chemical, and thermal stresses. Fourth, system-level integration and standardized qualification determine whether devices become products or remain prototypes. Robust, washable interconnects, distributed power and thermal-management schemes, and community-agreed test protocols are essential. Identifying these systemic interdependencies is therefore paramount, as it forms the logical prerequisite for a unified roadmap that translates fundamental breakthroughs into industrial reality.

The technological trajectory established in this roadmap, spanning from molecular- or nano-level engineering of nanomaterials to the hierarchical orchestration of textile systems, confirms that fiber electronics has matured beyond a collection of discrete fiber materials or fiber devices. The field is currently undergoing a fundamental paradigm shift from discrete laboratory curiosities to an integrated fiber-electronic systems paradigm, including multi-fiber assemblies and the system-in-a-fiber platform. This evolution represents a milestone leap in integration density, where every electronic function is natively embedded within a single filamentary substrate. By synergizing the unique electronic properties of 1D architectures with the comfort of traditional textiles, the foundation has been laid for electronics that are not merely flexible but are inherently human-compatible and bio-integrated. This bottom-up convergence ensures that the next decade of research will be governed by functional utility and systemic reliability, moving toward a future of textile intelligence.

**Figure 20.**
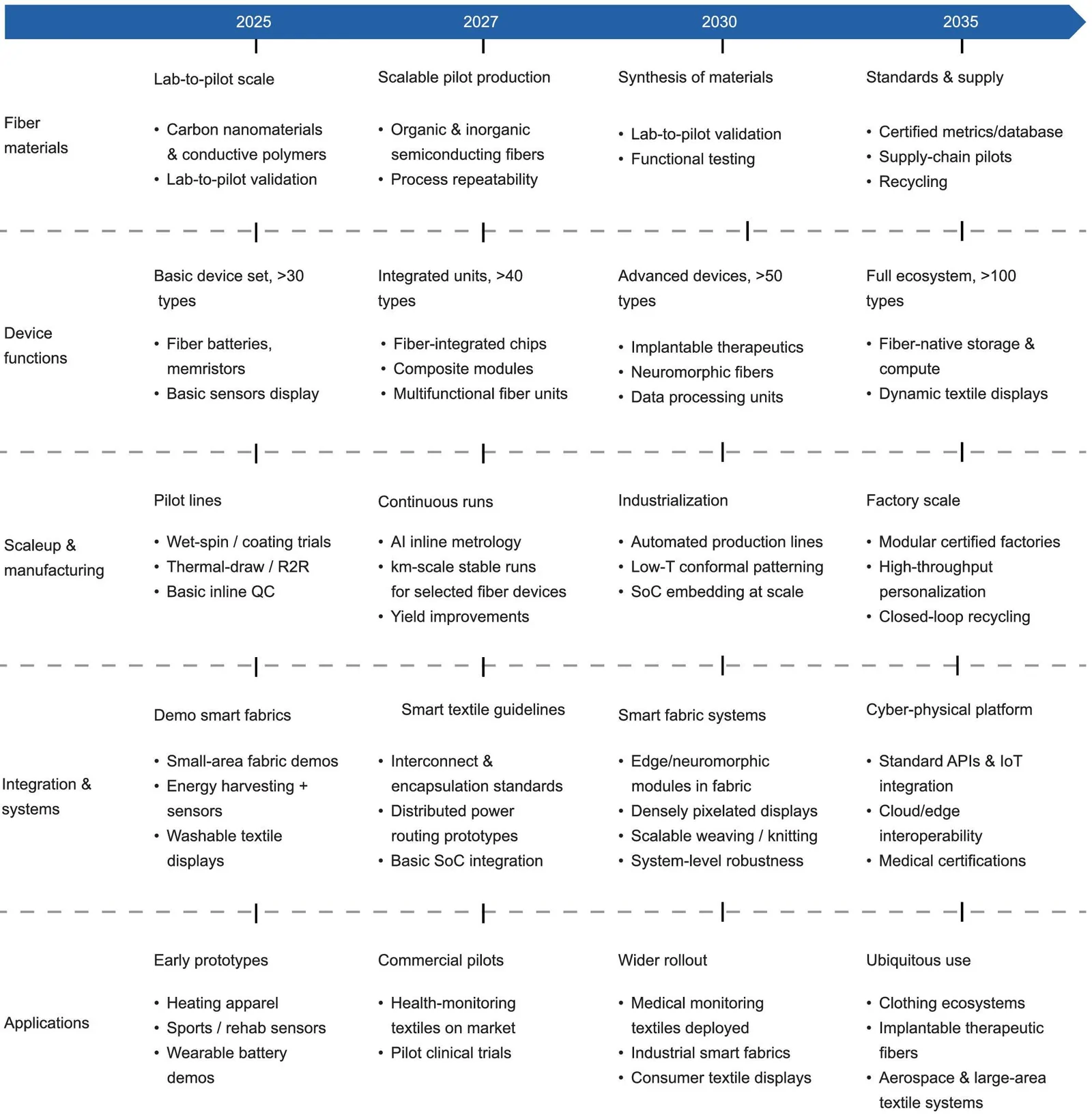
A decadal roadmap (2025–2035) for integrated-fiber electronic systems and textile intelligence. It outlines the coordinated evolution of the field over the next ten years, identifying staged milestones across five critical pillars: material innovation, device functionality, advanced manufacturing, system integration, and end-user applications. The timeline highlights the strategic shift from individual pilot-scale materials and discrete devices in 2025 toward the realization of standardized, scalable, and fully integrated fiber-based systems by 2035. This progression is characterized by the convergence of textile engineering, polymer science, and microelectronics to achieve a new era of textile intelligence.

Looking ahead, realizing fiber devices as a coherent technological platform, not a collection of isolated prototypes, requires coordinated progress along a few convergent frontiers (Fig. 20). Materials must become adaptive and purpose-driven. Beyond their conductivity and flexibility, fibers should embody durability, self-healing, tunable conductivity, and low-toxicity chemistries that are compatible with circular economy principles. Device engineering must be fiber-native rather than planar-minded. One-dimensional and woven morphologies should be exploited through coaxial multilayers, helical and hierarchical twists, and warp–weft pixelation to unlock axial transport, 360° optics, and intrinsically robust multifunctionality. Manufacturing must scale intelligently. Robotic spinning, thermal drawing, roll-to-roll patterning, and co-extrusion combined with low-temperature conformal deposition, inline metrology, and AI-driven process control are needed to achieve kilometer-scale throughput with consistent electrical, chemical, mechanical, and yield performance. System integration must follow. Textiles should be designed as cyber-physical fabrics in which thousands of cooperating fibers interconnect *via* standardized junctions, distributed energy and thermal management schemes, and textile-native driving and communication architectures so that sensing, computation, display, and power coexist natively in a single fiber or textile. Concurrently, application-driven validation, including the longitudinal field trials, clinical studies, aerospace/defense pilots, and early regulatory engagement, is essential for generating real-world reliability data and standardized test protocols (fatigue, washability, biocompatibility, and encapsulation lifetime) that bridge laboratory metrics and product readiness.

### Socio-technical architectures for sustainable and secure deployment

The transition toward integrated fiber electronic systems necessitates a parallel evolution in the socio-technical framework that governs their deployment, moving beyond laboratory feasibility toward industrial accountability. This pursuit of systemic reliability must be underpinned by a comprehensive framework addressing environmental circularity, data sovereignty, and global equity. To resolve the current ambiguity surrounding sustainability, the roadmap advocates for a circular-by-design mandate. Rather than relying on post-consumer sorting, which is notoriously difficult for heterogeneous textiles, actionable innovation should focus on selective disassembly *via* sacrificial interlayers. By engineering fibers with polymer matrices that respond to specific chemical or enzymatic triggers, the high-value active components can be recovered non-destructively from the textile waste stream. This technical pathway transforms the e-waste liability of smart textiles into a closed-loop resource model, ensuring that the vision of fiber electronics is not compromised by environmental degradation.

Equally critical is the establishment of hardware-rooted data sovereignty to protect the intimate physiological data harvested by fiber-native sensors. As these systems become pervasive, the traditional software-based encryption layer is insufficient for the unique multi-node topology of a woven garment. We propose transitioning to physical-layer security by exploiting the intrinsic stochasticity of the fiber manufacturing process. When coupled with decentralized on-fiber edge computing, where raw data is processed into feature tokens locally within the fiber-shaped transistors and chips, the attack surface is minimized, ensuring that high-resolution raw data never leaves the textile substrate. Finally, to prevent the technological divide often associated with advanced electronics, which requires multi-billion-dollar cleanrooms and facilities, an actionable recommendation is the development and standardization of open-source micro-extrusion or fiber-drawing platforms. By decentralizing the production of functional fibers through desktop-scale, high-throughput manufacturing protocols, the benefits of advanced healthcare monitoring and human–machine interfaces can be realized globally. This socio-technical integration ensures that the next generation of electronics is not only high-performing and flexible but also resilient, sustainable, and universally accessible, thereby aligning technical milestones with global socio-economic value.

### Practical bottlenecks and industrial realism

While the vision for 2035 remains ambitious, an honest assessment reveals a significant gap between current laboratory success and future industrial ubiquity. The primary practical bottleneck is the yield and reliability gap. While individual fiber devices often achieve over 90% success in controlled environments, industrial-scale integration, such as the fiber sensing systems required for long-term clinical monitoring, demands Six Sigma reliability. Bridging this gap requires the urgent standardization of chemical or mechanical fatigue protocols, moving from cycles-to-failure metrics to rigorous performance drift within specific standards under real-world dynamic deformation and complex environments. Furthermore, the energy-thermal paradox presents a looming challenge. As the integration density of functional fiber devices (i.e. logic gates, power units, etc.) increases, the traditional textile matrices will inevitably lead to localized overheating, negatively affecting the complex, high-speed computing within the fabric of everyday clothing. Delivering this agenda will require tightly coupled, interdisciplinary teams and new funding modalities that align stakeholders across the entire value chain. Chemists and materials scientists must create robust, recyclable functional fibers, while process engineers industrialize continuous, closed-loop manufacturing. Device physicists and textile engineers are tasked with realizing fiber-native circuits that function reliably within the mechanical constraints of a textile. Simultaneously, systems engineers, clinicians, and regulators must work in concert to validate and certify these end-to-end systems for real-world use. Funding models, translational incubators, and public–private partnerships that de-risk scale-up and align supply chains will be essential to accelerate the path from the laboratory to the market. When material sustainability, manufacturability, safety, and user experience are advanced together, fiber devices will stop being mere carriers of electronics and become the electronics themselves, remaking textiles into an inherently intelligent, interactive, and socially responsible platform for everyday life.

### Grand challenges beyond

Looking beyond the 2035 horizon, several fundamental scientific frontiers are likely to remain unresolved, serving as the ultimate targets for future research. The first is the long-term bio-interfacial paradox, which involves maintaining stable, low-impedance electronic transduction within harsh, ionic biological environments for periods exceeding 10 years. Even as we advance strategic projects, such as carbon nanotube fiber materials as artificial ligaments and fiber sensors for in-body biochemical variation monitoring through 2030, achieving decade-scale stability without resorting to rigid, hermetic encapsulation remains a grand challenge for bio-integrated electronics. The fundamental mismatch between dynamic biotic tissues and abiotic electronic interfaces requires a complete rethink of molecular packaging, moving from passive barriers to active-stealth interfaces that can biochemically and mechanically integrate into the host environment for the long term.

Another profound challenge lies in achieving geometry-independent electronic invariance at the molecular and device levels. Current fiber electronics are often evaluated based on their tolerance to strain or bending. However, a true system-in-a-fiber, or multi-fiber assemblies, must exhibit electronic properties that are fundamentally decoupled from its physical configuration. Achieving a state where carrier mobility, capacitance, or sensing gain remains strictly identical whether a fiber is straight, knotted, or stretched beyond 100% requires the discovery of new classes of topologically protected 1D materials. This necessitates a shift from traditional strain engineering toward the design of molecular architectures in which the electronic density of states is intrinsically invariant under extreme, nonlinear, and multi-axial mechanical deformation or other stimuli.

As we move toward hyper-integrated textiles containing millions of heterogeneous fiber nodes, we face the challenge of orchestrating collective functional emergence within stochastic networks. The current deterministic paradigm of circuit design, where every interconnect is pre-defined, will inevitably collapse under the sheer complexity and dynamic nature of woven systems. The grand challenge is to transition toward neuromorphic textile intelligence, in which the network itself learns to route signals and process information through collective behavior rather than fixed wiring. This involves developing a new theory of stochastic system engineering that can harness the random fiber-to-fiber contacts and parasitic impedances of a dense textile as a functional asset rather than a liability.

In conclusion, fiber devices now stand at a genuine inflection point. The scientific groundwork is laid, and industry pathways are visible, but success will hinge on translating interdisciplinary advances into durable, validated products and socially responsible ecosystems. Achieving that translation requires sustained collaboration across academia, industry, and regulators, along with pragmatic demonstration projects that expose real-world failure modes and de-risk scale-up. Successful translation will deliver textiles that are not mere carriers of electronics but electronics themselves, enabling pervasive, intimate, and equitable technologies woven into the very textile intelligence of everyday life.
